# PyMouseTracks: Flexible Computer Vision and RFID-Based System for Multiple Mouse Tracking and Behavioral Assessment

**DOI:** 10.1523/ENEURO.0127-22.2023

**Published:** 2023-05-12

**Authors:** Tony Fong, Hao Hu, Pankaj Gupta, Braeden Jury, Timothy H. Murphy

**Affiliations:** 1 Department of Psychiatry, University of British Columbia, Vancouver, British Columbia V6T 1Z3, Canada; 2 Djavad Mowafaghian Centre for Brain Health, University of British Columbia, Vancouver, British Columbia Canada V6T 1Z3

**Keywords:** multiple animal tracking, social interaction, stroke

## Abstract

PyMouseTracks (PMT) is a scalable and customizable computer vision and radio frequency identification (RFID)-based system for multiple rodent tracking and behavior assessment that can be set up within minutes in any user-defined arena at minimal cost. PMT is composed of the online Raspberry Pi (RPi)-based video and RFID acquisition with subsequent offline analysis tools. The system is capable of tracking up to six mice in experiments ranging from minutes to days. PMT maintained a minimum of 88% detections tracked with an overall accuracy >85% when compared with manual validation of videos containing one to four mice in a modified home-cage. As expected, chronic recording in home-cage revealed diurnal activity patterns. In open-field, it was observed that novel noncagemate mouse pairs exhibit more similarity in travel trajectory patterns than cagemate pairs over a 10-min period. Therefore, shared features within travel trajectories between animals may be a measure of sociability that has not been previously reported. Moreover, PMT can interface with open-source packages such as DeepLabCut and Traja for pose estimation and travel trajectory analysis, respectively. In combination with Traja, PMT resolved motor deficits exhibited in stroke animals. Overall, we present an affordable, open-sourced, and customizable/scalable mouse behavior recording and analysis system.

## Visual Abstract

**Figure:**
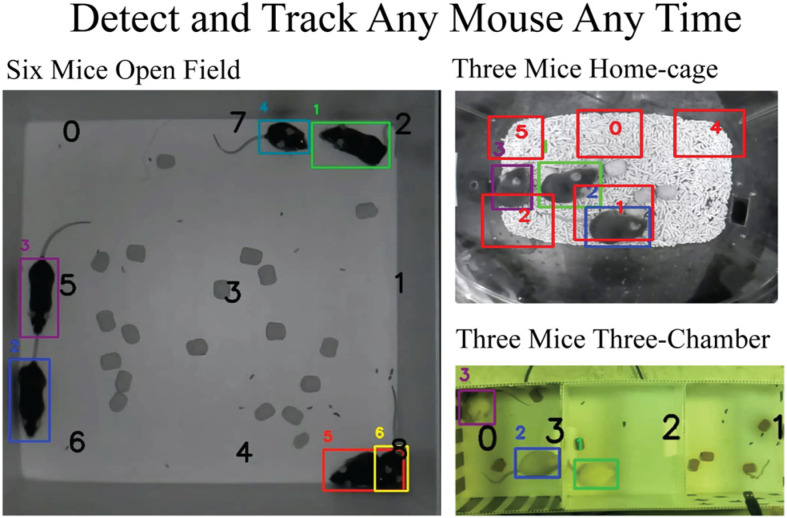

## Significance Statement

An affordable, customizable, and easy-to-use open-source rodent tracking system is described. Most current tools, commercial or otherwise, can only be fully automated to track multiple animals in a single defined environment and are not easily setup within custom arenas or cages. Moreover, many tools require users to have extensive hardware and software knowledge. In contrast, PyMouseTracks (PMT) is easy to install and can be adapted to track rodents of any coat color in any user-defined environment with few restrictions allowing quantification of behavior in multiple animals simultaneously.

## Introduction

Paradigms have been developed for identifying abnormal behavioral phenotypes in animal models of neuropsychiatric disorders (Sourioux et al., 2018). Traditionally, these approaches rely on manual phenotyping which is time, labor, and skill intensive. At the same time, results are not only prone to investigator bias and handling effects on animals (Ohayon et al., 2013), but also random errors dependent on the evaluator. As expected, results from traditional paradigms are usually high in interexperiment variability and can be difficult to reproduce (Kafkafi et al., 2018). Thus, there is an increasing need for behavior assays to be fully automated.

In recent years, a number of tools use technologies including combinations of computer vision, machine learning, neural networks, and physical tagging (de Chaumont et al., 2019; Romero-Ferrero et al., 2019; Singh et al., 2019; Kiryk et al., 2020; Segalin et al., 2021; Walter and Couzin, 2021) to automatically capture rodent behaviors. In particular, video tracking coupled with radio frequency identification (RFID) has become a popular and reliable approach for automatic identification of mice among groups without the use of visible markings (Bains et al., 2016; de Chaumont et al., 2019; Peleh et al., 2019). Physical tagging such as RFID on the animal is necessary for the complete automation of accurate tracking as each identity error (when mice cross or overlap) can propagate throughout the rest of the video without a method of periodic verification (Branson, 2014). Although there are markerless trackers available, these tools require: manual validation/correction of animal identities (Lauer et al., 2022), animals to be of different coat colors (Segalin et al., 2021) or require videos to have uniform lighting and high background contrast (Romero-Ferrero et al., 2019; Walter and Couzin, 2021). Such requirements render most current open-source automatic tracking systems restrictive and in turn have only narrow applications within particular arenas (de Chaumont et al., 2019) and experimental paradigms (Geuther et al., 2019).

We present PyMouseTracks (PMT): an affordable, open-source, easy-to-set-up, and customizable/scalable behavior recording software and hardware system. The system is capable of recording and tracking multiple mice of varied coat colors for extended periods of time in any user-defined environment. Video and RFID recordings use a Raspberry Pi (RPi) microcomputer. In the offline processing, mice are first detected and tracked in videos using the You Only Look Once version 4 (Yolov4) algorithm coupled with a modified version of the Simple Online and Realtime Tracking (SORT), respectively (Bewley et al., 2016; Bochkovskiy et al., 2020).

PMT home-cage recording used in our home-cage study contains six RFID readers and currently costs ∼520 USD per home-cage. The RFID reader numbers and locations can be adjusted to other home-cage variations or recording environments to fit a specific investigator’s need. To demonstrate flexibility and scalability, we also performed tracking of (1) six black coat-colored mice in an open-field, and (2) three white coat color mice in a three chambers arena with a low-contrast video background.

## Materials and Methods

### PMT recording setup

**Figure 1.**
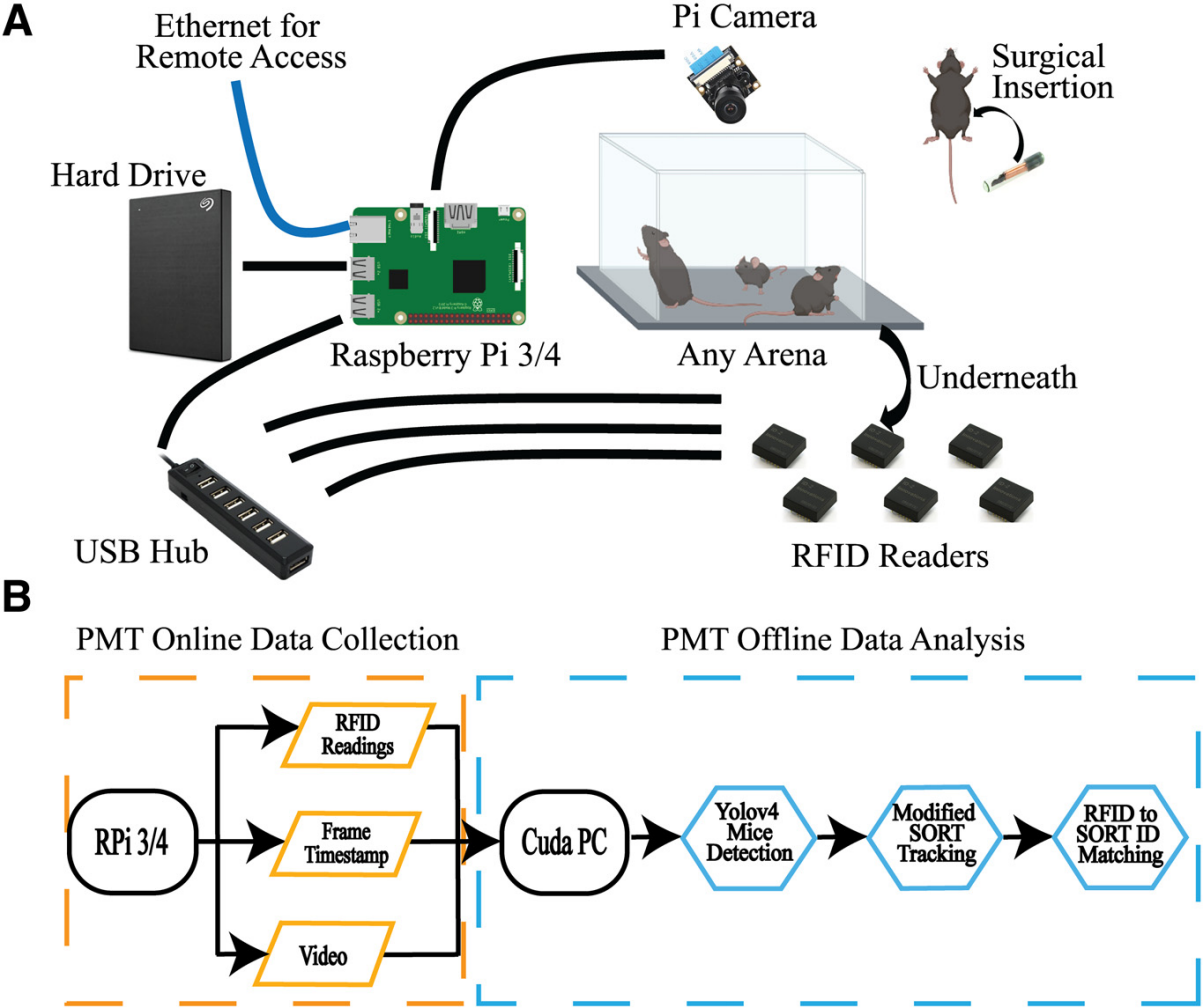
PMT data collection and offline data analysis. ***A***, The essential hardware and general recording setup of PMT. The recording can be done in any arena or cage. Main components include a RPi 3B+/4 connected to an overview Pi Camera of the arena, a powered USB hub to relay the RFID readers to the Pi, a hard drive for storage, and RFID readers underneath the arena. Mice being recorded have been surgically implanted with an RFID tag. When connected to ethernet, the system can be remotely accessed and controlled. ***B***, PMT RPi online data collection and offline analysis pipeline. Data are collected on a RPi 3B+/4, which records to a video file while simultaneously recording the timestamp of each frame and RFID readings from RFID readers. Data collected can be analyzed offline using a Colab notebook or a CUDA-capable PC. PMT: PyMouseTracks; RFID: Radio Frequency Identification.*Figure Contributions*: Tony Fong and Braeden Jury designed the system. Tony Fong wrote the software and composed the figure. This figure is supported by Extended Data 1, 2, and 3.

All components are connected to and controlled by a RPi 3B+/4 microcomputer running Raspbian Buster (https://www.raspberrypi.org) as seen in Figure 1*A*. All essential parts are listed in Table 1. During each recording, frames are written to an H264 video file while the timestamp of each frame was collected in a separate csv file. RFID antenna (Sparkfun, SEN-11828) and reader (Sparkfun, SEN-09963) output are also recorded in a separate file. Hardware installation is plug-and-play with off-the-shelf electronic components having few restrictions on the rodent arena employed (Movie 1, and Movie 2). The software is modular and customizable to control the data quality at various acquisition rates. A maximum video frame rate of 90 frames per second (fps) can be achieved at a resolution of 640 × 480 using a RPi V1 camera or equivalent (Waveshare, SKU10299). For further information on the possible frame rate and resolution please refer to the official picamera documentation (https://picamera.readthedocs.io/en/release-1.13/index.html#).

**Table 1:** Essential components for building the PMT online recording system

Component	Amount required	Supplier	Part number	Notes
Raspberry Pi 3B/4	1	Newark	RASPBERRYPI3-MODB-1GB/ RASPBERRYPI4-MODB-4GB	Either model could be used
RFID reader ID20-LA	Depends on user	Sparkfun	SEN-11828	As many as the user wants as long as they are spaced a minimum of 12 cm apart
RFID reader breakout	Depends on user	Sparkfun	SEN-09963	Matching to the number of RFID reader ID20-LA
USB mini B cable	Depends on user	AmazonBasics	N/A	Or equivalent
RFID glass capsules	1	Sparkfun	SEN-09416	One needed per animal to be tested
16GB micro-SD card	1	Scan	44082	Or equivalent
Pi camera (F/G)	1	Waveshare	10299/10344	Or any other Pi compatible camera
Portable hard drive	1	Seagate	STKC4000400	Or equivalent
RPi cooler	1	Smraza	SW49	Optional but recommended; other equivalent options could be used
Thermal paste	1	Noctua	NT-H1	Optional but recommended; other equivalent options could be used
PLA filament, 1.75 mm black	Depends on user	MakerBot	MP05775	Optional; to print custom 3D parts for home-cage design or RFID reader holder; Other equivalent options could be used

All essential components to build the PMT online recording system. Aside from the RFID tags, antennae, and reader breakout board, all components can be purchased at alternative suppliers. The number of RFID reader modules is decided by the use.

10.1523/ENEURO.0127-22.2023.ext1 Extended Data 1. PMT online data collection module. Complete software package for online data recording. The package should be installed on a RPi microcomputer. *Figure Contributions*: Tony Fong wrote, tested the software, analyzed, and created the video. This extended data file supports Figure 1. Download Extended Data 1, ZIP file.

10.1523/ENEURO.0127-22.2023.ext2 Extended Data 2. PMT offline data analysis module. Complete software package for offline data analysis. The package was installed and ran in an Anaconda environment (https://www.anaconda.com). Code was tested on a Windows 10 PC (AMD Ryzen 7 5800X; 64 GB; RTX 2080Ti) and Linux (Ubuntu 20.04.2 LTS) PC (Intel i7-7800X; 94 GB; GTX Titan X). *Figure Contributions*: Tony Fong wrote and tested the software. This extended data file supports Figure 1. Download Extended Data 2, ZIP file.

10.1523/ENEURO.0127-22.2023.ext3 Extended Data 3. Assembly and installation instructions. A complete guide on setting up the hardware components and installing the software for PMT. Links to video tutorials and demonstrations can also be found in the guide. Download Extended Data 3, DOCX file

Movie 1. PMT software setup on a RPi. A detailed walkthrough of setting up the software of the PMT online recording system on a RPi. This tutorial is also intended for users with a more limited background in coding or use of Linux-based systems. All related commands to clone and install software from the GitHub repository are included. PMT: PyMouseTracks; RPi: Raspberry Pi.*Video Contributions*: Tony Fong analyzed and created the video. 10.1523/ENEURO.0127-22.2023.video.1

Movie 2. PMT demo hardware setup. A demonstration of setting up the PMT in an open-field arena (32 × 32 cm) with nine RFID readers. The total duration of the setup is less than 30 min. Extra tools include M1 screws/nuts and a glue gun. PMT: PyMouseTracks.*Video Contributions*: Tony Fong analyzed and created the video. 10.1523/ENEURO.0127-22.2023.video.2

In brief, a Pi-camera is situated above the rodent arena with an unobstructed view. RFID readers are connected to RPi through a self-powered USB hub and placed underneath the arena. The number of RFID tag readers used can be adjusted to arenas of any dimension as long as they are spaced 12 cm apart to minimize RFID interference. Currently, up to nine readers have been tested in a single setup. The RPi board can thermal throttle leading to decreases in performances such as drops in frame rate and RFID readings. Therefore, we recommend installing a supplemental cooling solution (listed in Table 1).

### PMT offline analysis pipeline

**Figure 2.**
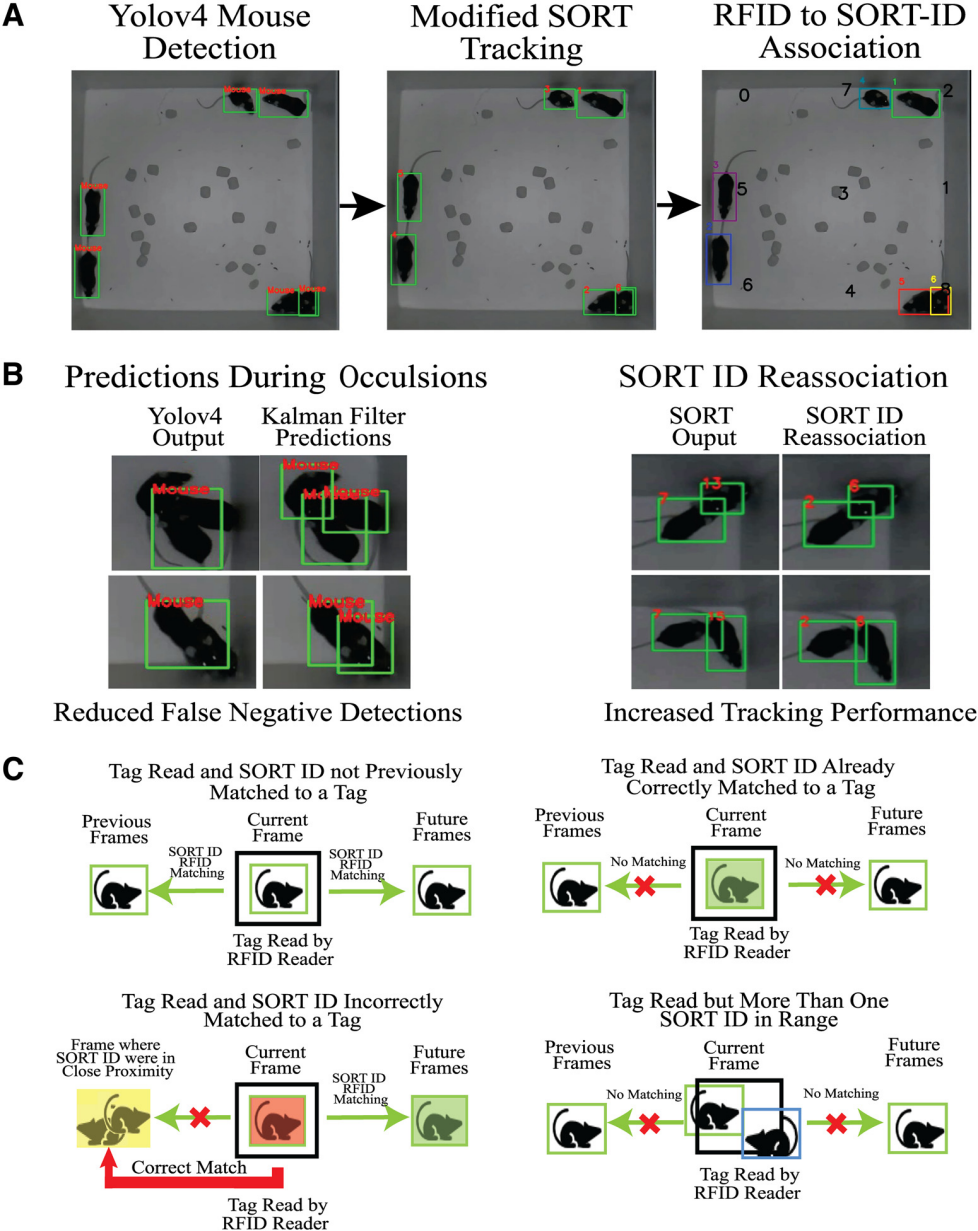
Overview of the PMT offline analysis pipeline. ***A***, Mice are first detected by Yolov4 and tracked using a modified SORT tracker. Then the SORT IDs generated from the SORT tracker are temporally matched to RFID tags read by the RFID reader. Each black bold number represents an RFID reader underneath the arena. ***B***, Main features of the modified SORT tracker. In cases of Yolov4 detection failures such as visual occlusions or close proximity of mice, the Kalman filter will still output predictions of mice. The second feature is the re-association of false-positive SORT-ID to an old SORT-ID that disappeared to increase tracking performance. ***C***, The possible scenarios and SORT-ID to RFID matching outcomes when an RFID tag is read by an RFID tag reader. PMT: PyMouseTracks; Yolov4: You Only Look Once Version 4; SORT: Simple Online and Realtime Tracking; RFID: Radio Frequency Identification; SORT-ID: Simple Online and Realtime Tracking Identification.*Figure Contributions*: Tony Fong designed the pipeline and composed the figure.

The PMT offline analysis pipeline runs on a computer capable of CUDA-processing, using Python3.7, managed in an Anaconda environment (https://www.anaconda.com) and has been tested on Windows 10 and Ubuntu 20.04 operating systems. The pipeline contains three components as shown in Figures 1*B* and 2*A*: (1) deep-learning-based Yolov4 for mouse detection (Bochkovskiy et al., 2020), (2) modified SORT for mouse tracking (Bewley et al., 2016), and (3) RFID to SORT ID matching for identity assignments.

First, bounding boxes around mice are detected by Yolov4 (Bochkovskiy et al., 2020). All such bounding boxes are then assigned an ID by a modified SORT algorithm, which uses a Kalman filter to predict and track each mouse to preserve the identities. As shown in Figure 2*B*, two features were added to the SORT algorithm: (1) the Kalman filter predictions for lost tracks and (2) SORT-ID reassociation to enhance tracking performance. The Kalman filter can predict mouse position based on its previous positions, and is useful when tracking by Yolov4 fails, for example, because of visual occlusion or overlapping detections. Similarly, new false positive IDs can be assigned to a previous bounding-box that disappeared. In other words, occasionally, new SORT IDs can be generated for the same mouse, discarding a previously tracked ID. The generation of these new false SORT-IDs is because of the sudden changes in travel trajectories of mice and are difficult to be fully predicted by SORT’s underlying Kalman filter. Regardless, the modified SORT algorithm provides highly accurate tracking of individual animals during clustering scenarios (Movie 3).

Movie 3. Modified SORT algorithm tracking during high occlusion and clustering situations. A demonstration of the modified SORT algorithm tracking mice during scenarios of occlusions and mice clumping/clustering together. With Yolov4 and the original SORT algorithm, many detections are lost. SORT: Simple Online and Realtime Tracking; Yolov4: You Only Look Once Version 4.*Video Contributions*: Tony Fong analyzed and created the video. 10.1523/ENEURO.0127-22.2023.video.3

In the last stage of PMT offline analysis, the SORT IDs are matched to RFID readings. The general overview of the matching process is illustrated in Figure 2*C*. RFID reader locations are user defined regions of interest in the video. When a tag is read by an RFID reader, there are four possible scenarios which then, in turn, can lead to three possible outcomes: (1) ID to RFID matching in all previous and future frames, (2) no matching, or (3) matching of future frames and correction of previous frames to a point of occlusion. Both centroid distance and intersection over union (IOU) were used to determine whether an ID detection is in the range of an RFID reader. In the first scenario, the ID has not previously been matched to a tag and therefore, it will be matched with the tag in all previous and future frames. In the second scenario, the ID has already been correctly matched to the tag being read, so no matching would occur. In the third scenario, there is more than one ID (i.e., more than one mouse) in range of the RFID reader. To ensure clean RFID matching, again no matching would occur. In the final scenario, the ID is incorrectly matched to a tag. Therefore, all ID to RFID matches in future frames and previous frames are corrected. Corrections occur up to a point where the ID was in proximity with another, a situation where identity swaps are likely to occur due the use of IOU.

### Yolov4 training and weight generation

Weights for Yolov4 detection were trained in its original Darknet (Bochkovskiy et al., 2020) implementation through transfer learning using pretrained weights as the performance has not been fully reproduced on the TensorFlow 2 framework (Hùng, 2021). Trained weights were then converted to Tensorflow weights for the detection to run natively in Python. For the home-cage experiments using dark C57BL/6 black mice, weights were trained using over 2500 random, distinctive images from multiple experiment sessions observing three to four mice for 1–2 h at 25 fps. Similarly, weights for the open-field with six dark C57BL/6 black mice and three-chamber experiments with three FVB/N mice were trained on 300 random, distinctive images recorded at 40 and 60 fps, respectively, for 10 min. During Yolov4 training, training and validation split were set to 80 and 20%, respectively All testing of system reliability were conducted with videos collected independent of training data. A minimum mean average precision (mAP) value >99.5% was achieved on the test dataset for all weights (Extended Data Fig. 3-1).

**Figure 3.**
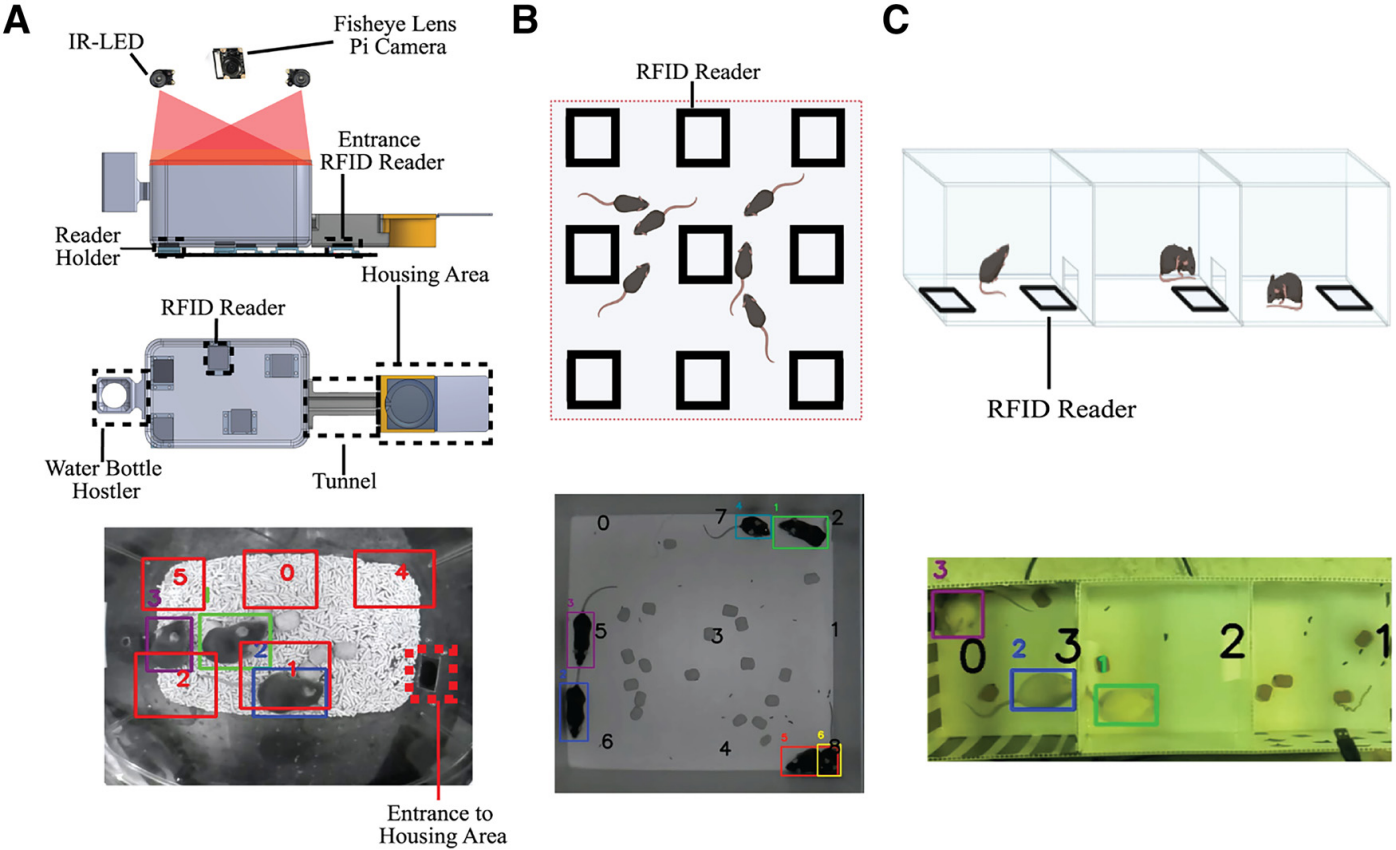
PMT Demonstration Setups and Variants. ***A***, The custom home-cage for chronic behavior recording. A fish-eye lens Pi camera and two IR lights are situated at the top of the cage separated by an acrylic sheet. The cage sits on top of the RFID readers which are held in place by holders. A tunnel is connected to the main cage from one end to the housing area, while the other end holds a water bottle hostler. All home-cage videos were recorded at a resolution of 512 × 400 that was still sufficient to resolve the animals. RFID reader locations are denoted by numbers 0–5 with red rectangles. ***B***, PMT recording under IR light and analysis in an open-field arena with six black coat color-coated mice. Videos was recorded at 960 × 960 at 40 fps. ***C***, PMT recording under natural light in a three-chamber sociability arena with 3 white coat color-coated mice. The Video was recorded at 640 × 480 at 60 fps. Each color and corresponding ID represent RFID tracking of an individual mouse. The bold black numbers represent the RFID reader ID recognized by the system. RFID: Radio Frequency Identification; PMT: PyMouseTracks; fps: Frames per Second; ID: Identification. *Figure Contributions*: Tony Fong designed the system and composed the figure. This figure is supported by Extended Data Figures 3-1*A–C* and 3-2.

10.1523/ENEURO.0127-22.2023.f3-1 Extended Data Figure 3-1 Yolov4 Training Loss and mAP. ***A***, Training loss and mAP on home-cage weights using 2500 images with 6000 iterations. ***B***, Training loss and mAP on the open-field arena using 300 images with 6000 iterations. The training was manually stopped when mAP > 99.5%. ***C***, Training loss and mAP on the sociability chamber arena using 300 images with 6000 iterations. The training was manually stopped when mAP > 99.5. *Figure Contributions*: Tony Fong labeled the images and trained the weights. This extended data figure supports Figure 3. Download Figure 3-1, TIF file.

10.1523/ENEURO.0127-22.2023.f3-2 Extended Data Figure 3-2 Evaluation of PMT performance on the open-field arena with six mice. ***A***, PMT detection Performance was measured by total detection, the appearance of false negative detections, and false positive detections. False positive and false negative detections were expressed as a % of total ground truth mice in videos. ***B***, Tracking performance of PMT was measured by coverage, MOTA, and % errors in detections with RFID matched. Coverage represents % of detections being matched with an RFID tag. A total of two videos were evaluated. *Figure Contributions*: Tony Fong and Hao Hu analyzed the data. Tony Fong created the figure. This extended data figure supports Figure 3. Download Figure 3-2, TIF file.

### Motion detection and trajectory analysis

To identify and segregate keyframes of interest, i.e., frames containing mice with active movements, a simple motion detector is built into the PMT pipeline. Background subtraction against an accumulated average between current and previous frames was used to detect consecutive frames of motion. Specifically, a Gaussian filter was applied to average pixel intensities across a region of 21 × 21. An absolute value between the current frame and the accumulated weights of previous frames was calculated to yield contours representing regions of motion. Specific settings of the motion detector can also be adjusted within the offline tracking pipeline. Travel trajectories are first smoothed using the Ramer–Douglas–Peucker algorithm (ε = 10; Sharmila and Sabarish, 2021) and analyzed using Traja (Shenk et al., 2020) by the input of center coordinates outputted by PMT.

### Manual validation of videos

To evaluate the performance of PMT, 10-min videos were recorded at 15 fps at 512 × 400 from the modified home-cage containing one to four animals. Two videos were recorded at 30 fps at 960 × 960 in the open-field arena and one video at 30 fps at 640 × 480 in the three-chamber arena. Videos were then evaluated frame for frame by students for appearances of false positive detections (FP), false negative detections (FN), incorrect ID and RFID matches. In each video, the total number of mice in ground truth (all frames) was calculated by the addition of false negative detections and subtraction of false positive detections found. FP and FN are expressed as a percentage of ground truth mice calculated, whereas incorrect ID and RFID matches are expressed as a percentage of total matches made. To objectively compare PMT to existing object trackers, the Multiple Object Tracking Accuracy (MOTA) index proposed by Bernardin et al. (2006) was used. Specifically, MOTA was calculated by the following equation:

MOTA = 1 − sum(f) (FN (f) + FP(f) + identity error(f))/sum(f) (number of mice in the ground truth),

f is defined as a frame. FN is the total number of mice not detected (but should be there); FP is the total number of mice detected (but not actually there). An identity error is the total number of mice labeled with an incorrect RFID tag; and the number of mice in ground truth is the number of mice that should be tracked in each frame. Total false negatives, total false positives, and total identity errors were determined by student evaluators examining each videos frame by frame.

### Home-cage recording setup and PMT pipeline line adjustments

To perform 3-d chronic recordings in a home-cage, a shoebox-sized mouse home-cage (19 × 29 × 12.7 cm) was modified to hold a water bottle holder, a housing area, and its connecting tunnel as seen in Figure 3*A*. For video capture, a single fisheye lens Pi-camera [RPi Camera (I), fisheye lens; SKU:10 703; angle of view: 160°] and IR lights were fitted to the top of the cage. A RFID reader is attached underneath the tunnel leading to the mouse housing area. A custom-cut acrylic sheet is also placed between the Pi camera with its related wirings and the cage main body. A total of 30 ml of pellet bedding and food pellets were distributed on the floor of the main cage. A pellet-type bedding was used for home-cage recording as less clumping would occur and therefore minimize animal RFID tag distance to readers, however other forms of bedding should work, provided it is not too deep; deep bedding would increase the distance from tags to readers. Recordings were done in 12-h intervals and were temporarily interrupted for 15 min every 2 d for cage cleaning. For performance validation, 15 10-min videos were recorded at 25 fps containing one to four mice.

Modifications to the offline tracking pipeline were made so that all matching processes from cage RFID readers stop at a certain distance to the housing area entrance as seen in Figure 3*C*. The final modification was the use of an entrance RFID reader (underneath the tunnel to the housing area) for ID to RFID matching. The reasons for these modifications are that the entrance will provide scenarios where a new mouse may appear, a tracked mouse may disappear, or both may occur.

### Open-field and three chamber recording

Nine RFID readers were placed underneath an open-field (32 × 32 cm) as illustrated in Figure 3*B*. When recording, the open-field arena was placed in a chamber covered by black-out curtains. Two videos were recorded at a resolution of 960 × 960 under IR light at 40 fps using a regular lens Pi camera for analysis.

For three chamber testing, four RFID readers were placed underneath an arena (20 × 20 cm for each chamber) as illustrated in Figure 3*C*. Videos were recorded at a resolution of 640 × 480 under natural background lights using a regular lens Pi camera. One 10-min video recorded at 60 fps was used for analysis.

### Animals

Male C57BL/6 mice three to four months old (unless indicated) of varied genotypes were used for the behavior recording in the custom home-cage and the open-field arena. We are not reporting the genotype as the experiments were not powered to make comparisons between different animals. Instead, our goal was to evaluate tracking accuracy using surplus animals and will reserve cross-genotype work for future studies. FVB/N mice that were six months old and of varied genotypes were used for behavior recording in the three-chamber arena. Mice were group-housed in a conventional facility in plastic cages similar to the home-cage setup and kept under a normal 12/12 h light/dark cycle (7:00 A.M lights on). All procedures were conducted with approval from the University of British Columbia and in accordance with national guidelines.

### RFID capsule implantation

To enable the identification of mice, animals were implanted with glass RFID capsules (Sparkfun, SEN-09416) before recording. RFID capsules were sterilized with ethanol before each implantation. Animals were anesthetized with isoflurane and given buprenorphine via subcutaneous injection (0.05 mg/kg) for analgesia. Betadine was applied to disinfect the incision site and a small incision was made below the nape of the neck or in the lower abdomen, depending on RFID placement. A sterile injector (Fofia, ZS006) was then used to insert the RFID capsule subcutaneously below the nape of the neck or at the abdomen (abdominal will yield better performance). RFID capsules were sterilized with ethanol before each use. Only animals in the three-chamber test received RFID implants through the neck, the rest received abdominal implants. The incision was sutured, and the animal was removed from anesthesia, allowed to recover, and then returned to its home-cage. Animals were closely monitored for a minimum of one week to ensure healthy recovery and proper placement of the RFID capsule postsurgery.

### Stroke induction

A photothrombotic occlusion was introduced at a target area between the sensory and motor cortex, stereotactic coordinates (1.5; 0.5) mm from bregma. Mice were first fitted with a chronic transcranial window. In brief, animals were anesthetized with isoflurane (2% in pure O_2_) and the skin covering the skull was removed and replaced with a cranial window fixed with dental cement. For photothrombotic occlusion, mice were injected intraperitoneally (0.1 ml/10 g body weight) with a photosensitive dye solution Rose Bengal (RB; R3877-5G, Sigma-Aldrich). Two minutes after the injection, a 40-mW diode pump solid-state 532-nm laser attenuated to 11 mW through a polarizer was turned on at the target area to induce focal ischemia. The final beam diameter measures 1.2 mm at full width at half max amplitude. Previous studies show that tissue damage is limited to the targeted area of the green laser irradiation when combined with Rose Bengal administration (Schaffer et al., 2006). Ten-minute behavior recordings of individual mice were performed at 1-h prestroke, 1 d poststroke, and 7 d poststroke in the open field illustrated in Figure 3*B*. Travel trajectories are first smoothed using the Ramer–Douglas–Peucker algorithm (ε = 10) and analyzed using Traja. The center area of the arena is defined as 0.5*width and 0.5*length around the center of the open-field arena.

### Social stimulus test

A test mouse is left in the open-field arena, as described above, with a stimulus mouse cagemate (mouse from the same cage) or a noncagemate (mouse from a different cage) for 10 min. For all trials, the test mouse was first tested with a cagemate and later with a noncagemate with a 20-min gap between trials. As the same mouse within a cage was used as the cagemate mouse, tests with the cagemate first prevented any crossover in odor from a noncagemate mouse. The cage was cleaned with 70% ethanol between trials.

Track pattern difference score is calculated using the dynamic time warping algorithm published by the DTAI Research Group (Shokoohi-Yekta et al., 2017). Both trajectories were z-normalized before dynamic time warping alignment to calculate a track difference score expressed in Euclidean distance. Spatial proximity (SP) is calculated by the average of all minimal distances of each point on a trajectory to the other and is also expressed in Euclidean distance. Distal trajectory pairs were defined as trajectories segments with an SP >300, whereas proximal trajectory pairs were defined as trajectories segments with an SP <300. Track difference pattern score was expressed as an average for each trial per test animal.

### Traditional social interaction detection

Social interaction (ITC) was calculated by the sum duration of overlap of enlarged (25% area) bounding boxes of detected mice. An ITC episode was defined as the duration between onset and offset of the overlap between mice bounding boxes. Number of ITC episodes were calculated by the count of ITC episodes and the average duration of ITC episodes were also calculated.

### Statistical analysis

Data are all presented as mean ± SEM. Statistical significance was determined using either a multivariate regression analysis (MANOVA) followed by a *post hoc* univariate ANOVA or repeated measures ANOVA (RM-ANOVA) followed by paired Student’s *t* tests (with Bonferroni correction) as appropriate using R. The level of significance is denoted on the figures as follows: *p <* *0.05, **p <* *0.01, and ***p <* *0.001.

### Code accessibility

The code/software for PMT online recording and offline analysis is available at https://github.com/tf4ong/tracker_rpi and https://github.com/tf4ong/PMT, respectively. The code is also available as Extended Data 1 and 2, respectively. All data are available at the open science framework https://osf.io/78akz/.

A full guide to setup and use the online and offline system can be found in the https://github.com/tf4ong/PMT page and as Extended Data 3. Video tutorials and demonstrations can be found at the following link https://youtube.com/playlist?list=PLmcjDqLt_Xk6AAlll3ztvgNI9P3yQxPc2.

### Custom home-cage parts

All related CAD files can be found at our GitHub: https://github.com/tf4ong/PMT.

### 3D-printed parts (black PLA)

RFID_reader_base.stl

Nest_tunnel.stl

Nest_body.stl

Nest_lid.stl

Camera_LED_mount.stl

Cage_Lid.stl

### Data availability

All data will be uploaded to the OSF data repository: https://osf.io/78akz/.

## Results

### PMT detection and tracking performance in home-cage

**Figure 4.**
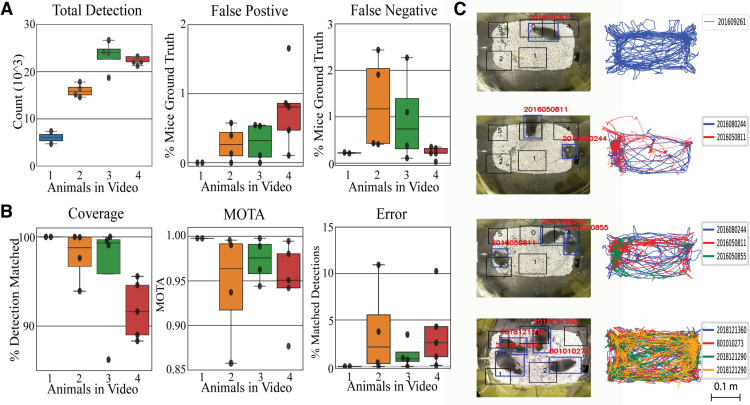
Evaluation of PMT performance in the modified home-cage. ***A***, PMT detection performance as measured by total detection, appearances of false negative detections, and false positive detections which were scored by student evaluators. False positive and false negative detections were expressed as a % of total number of ground truth mice in each video. The total number of ground truth mice was calculated by the subtraction of false positive and addition of false negative mice to the total number of detections made by Yolov4 in each video. Each individual data point represents a single 10-min video. ***B***, Tracking the performance of PMT was measured by coverage, MOTA, and % errors in detections with RFID matched. Coverage represents % of detections being matched with an RFID tag in each video. Identity error is % of matched detection with an incorrect RFID tag. The number of identity errors were determined by student evaluators on a frame-to-frame basis. Each individual data point represents a single 10-min video recorded in the home-cage. ***C***, Sample image and travel trajectory of mice in the modified home-cage for cases with one to four mice. MOTA: Multiple Object Tracking Accuracy; PMT: PyMouseTracks; Yolov4: You Only Look Once Version 4; MOTA: Multiple Object; Tracking Accuracy; RFID: Radio Frequency Identification.*Figure Contributions*: Tony Fong and Hao Hu analyzed the data. Tony Fong composed the figure.

In Materials and Methods, we describe the setup and implementation of PMT for both standard “shoebox-sized” mouse cages and more complex arenas (Fig. 3). To evaluate PMT’s performance, one to four mice were placed in the modified home-cage for 10 min recordings at 15 frames per second (Fig. 4). Results were compared with manual human frame-by-frame validation. Figure 4*A* shows the detection reliability of the pipeline. Both the FP and FN detections remain extremely low in all 14 videos analyzed, never exceeding 3% of the total number of mice in manually labeled ground truth. On average, the FP rate were 0, 4.3, 10.5, and 19.9 per minute for videos containing one to four animals, respectively. Whereas, FN rate were 1.7, 20, 22.1, and 3.6 per minute. In regard to tracking, performance was mainly evaluated by coverage, MOTA value, and identity error rate as seen in Figure 4*B*. Coverage is represented as the percentage of mice detected that are matched with an RFID tag and remained above 90% in most videos analyzed. Specifically, the average coverage for one to four animals was 100.0, 97.9, 96.2, and 91.8%, respectively. Among the detections matched with an RFID tag, the identity error rate, i.e., the percent of detections with incorrectly matched RFID tags, was on average 0, 4, 1, and 4% of all matched detections in videos of one to four animals. Each 10-min video would contain on average 6 episodes where detections were matched with an incorrect RFID tag (∼45 frames in length). In our validation set, the minimal value achieved for detection coverage and identity accuracy were 88% and 85%, respectively.

The Multiple Object Tracking Accuracy (MOTA) index proposed by Bernardin et al. (2006) is currently one of the main metrics for determining the effectiveness of a multiple object trackers (de Chaumont et al., 2019; Lauer et al., 2022). MOTA evaluates a tracker’s overall performance by calculating metrics which represent errors in both detection and tracking including false positive/negative detections and identity mismatches, respectively. Therefore, a tracker with no false positive/negative detection and no identity tracking would yield a MOTA value of 1. In the current study, the average MOTA value for videos containing one, two, three, and four animals were calculated to be 0.998, 0.945, 0.973, and 0.949, respectively. As a point of reference, the MOTA values for live mouse tracker (LMT; de Chaumont et al., 2019) on videos with one, two, three, and four animals are 0.993, 0.991, 0.984, and 0.970, respectively. However, LMT only used one video to conduct MOTA calculations. Sample images and travel trajectories of mice can be observed in Figure 4*C* and in Movie 4, 5, 6, 7.

Movie 4. PMT Tracking of one mouse in custom-built home-cage. Sample video recording of one mouse in the modified home cage (19 × 29 × 12.7 cm). In cage, RFID reader locations are denoted by numbered bounding boxes. The entrance RFID reader underneath the tunnel connecting the housing area to the main cage. Video recorded at resolution of 512 × 400 at 15 frames per second under IR illumination. PMT: PyMouseTracks; RFID: Radio Frequency Identification; IR: Infrared. *Video Contributions*: Tony Fong analyzed and created the video. 10.1523/ENEURO.0127-22.2023.video.4

Movie 5. PMT Tracking of two mice in custom-built home-cage. Sample video recording of two mice in the modified home cage (19 × 29 × 12.7 cm). In cage, RFID reader locations are denoted by numbered bounding boxes. The entrance RFID reader underneath the tunnel connecting the housing area to the main cage. Video recorded at resolution of 512 × 400 at 15 frames per second under IR illumination. PMT: PyMouseTracks; RFID: Radio Frequency Identification; IR: Infrared.*Video Contributions*: Tony Fong analyzed and created the video. 10.1523/ENEURO.0127-22.2023.video.5

Movie 6. PMT Tracking of three mice in custom-built home-cage. Sample video recording of three mice in the modified home cage (19 × 29 × 12.7 cm). In cage, RFID reader locations are denoted by numbered bounding boxes. The entrance RFID reader underneath the tunnel connecting the housing area to the main cage. Video recorded at resolution of 512 × 400 at 15 frames per second under IR illumination. PMT: PyMouseTracks; RFID: Radio Frequency Identification; IR: Infrared. *Video Contributions*: Tony Fong analyzed and created the video. 10.1523/ENEURO.0127-22.2023.video.6

Movie 7. PMT Tracking of four mice in custom-built home-cage. Sample video recording of four mice in the modified home cage (19 × 29 × 12.7 cm). In cage, RFID reader locations are denoted by numbered bounding boxes. The entrance RFID reader underneath the tunnel connecting the housing area to the main cage. Video recorded at resolution of 512 × 400 at 15 frames per second under IR illumination. PMT: PyMouseTracks; RFID: Radio Frequency Identification; IR: Infrared. *Video Contributions*: Tony Fong analyzed and created the video. 10.1523/ENEURO.0127-22.2023.video.7

### PMT detection and tracking performance in open-field and three chamber arena

To evaluate the scalability and customizability of the PMT tracker, additional behavior recordings were performed in an open field arena with dark C57BL/6 black mice and in a three-chamber sociability arena with lighter coat color FVB/N mice. In total, two videos were analyzed to evaluate the performance of PMT in an open field arena in a similar fashion illustrated in Figure 3*B*. In the two videos, FP and FN detections remained under 1% of total mice in ground truth (Extended Data Fig. 3-2; Movie 8). In these larger arenas, virtually all detections can be matched with an RFID tag, with an average coverage of 99.99% between the two videos. Moreover, MOTA values were also very high in the two videos averaging at 0.97. Surprisingly, PMT achieved a very low identity error rate with that of both videos being <4%.

Movie 8. PMT Tracking of six mice in an open-field with nine RFID readers. Sample video recording of six mice in the modified home cage (32 × 32 cm). RFID reader locations is denoted by a numbered bounding box. Video recorded at resolution of 960 × 960 at 40 frames per second under IR illumination. PMT: PyMouseTracks; RFID: Radio Frequency Identification; IR: Infrared. *Video Contributions*: Tony Fong analyzed and created the video. 10.1523/ENEURO.0127-22.2023.video.8

For the three-chamber arena, performance was similar as in the open-field arena. After retraining Yolov4 on white coat color mice detection, FN and FP were 0.03 and 0.14% respectively. MOTA index value was 0.965 and the identity error rate was 3.33% of all RFID matched detections. Sample illustrations of PMT tracking in a three-chamber arena can be observed in Figure 3*C* and in Movie 9 based on a single video example.

Movie 9. PMT Tracking of three white color-coated mice in a three-chamber arena. Sample video recording of three white color-coated mice in a three-chamber arena, each chamber is 20 × 20 cm in area. RFID reader locations are denoted by numbered bounding boxes. Video recorded at resolution of 640 × 480 at 40 frames per second under room light. PMT: PyMouseTracks; RFID: Radio Frequency Identification; IR: Infrared. *Video Contributions*: Tony Fong analyzed and created the video. 10.1523/ENEURO.0127-22.2023.video.9

### Stroke induced changes in open-field behavior

**Figure 5.**
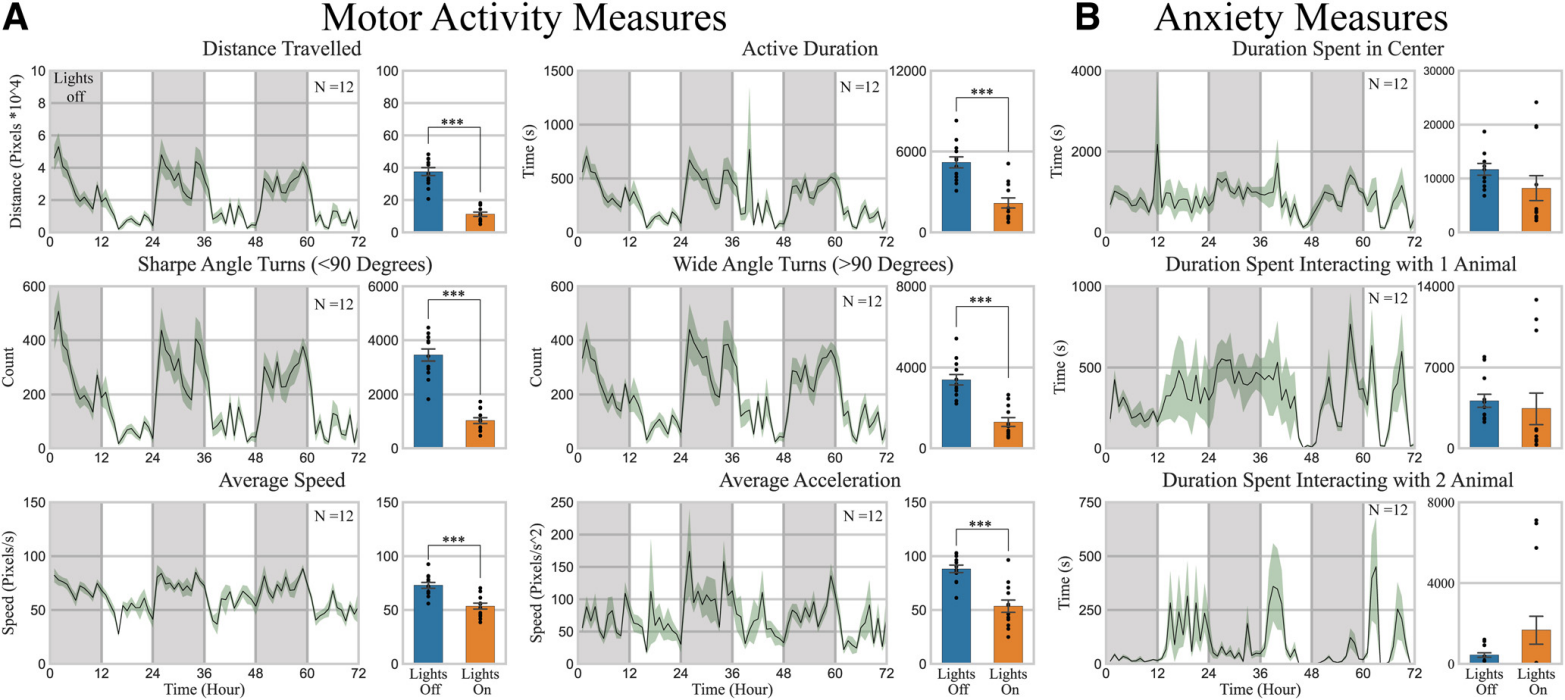
Ten-minute open-field of mice prestroke, 1 and 7 d poststroke induction. ***A***, Sample travel trajectory of a mouse before, 1 d, and 7 d poststroke induction. ***B***, Turn angle distribution of all stroke induced mice in open-field: prestroke versus 1 d poststroke and prestroke versus 7 d poststroke. ***C***, Significant changes in travel trajectory parameters detected by a one-way repeated ANOVA followed by a pared student’s test with Bonferroni correction. *p < 0.05 and **p < 0.01 (*N* = 4 mice). Data are presented as the mean ± SEM. *Figure Contributions*: Tony Fong and Pankaj Gupta performed the experiment and analyzed the data. Tony Fong composed the figure. This figure is supported by Extended Data Figure 5-1.

A total of four mice were recorded and used for analysis to pilot whether PMT can resolve measurable changes because of stroke induction. Figure 5*A* illustrates a sample travel trajectory of a mouse prestroke, 1 d poststroke, and 7 d poststroke to the sensory-motor cortex. Consistent with the literature, stroke acutely induces motor impairments in mice observable in the open field (Bains et al., 2016). Turn angle distributions of prestroke versus 1 d after stroke showed a consistent pattern of decreased number of sharp angle turns (<45°) as shown in Figure 5*B*. Further analysis revealed statistically significant changes in mean speed (df = 2, *F* = 8.018, *p* < 0.05) and number of sharp angle turns (df = 2; *F* = 10.16, *p* < 0.05) between prestroke and 1 d poststroke. as shown in Figure 5*C*. Surprisingly, no difference was found between distance traveled between prestroke to post 1 or 7 d after stroke. Duration spent in center (df = 2, *F* = 11.13, *p* < 0.01) was also different between prestroke and 1 d poststroke in animals.

10.1523/ENEURO.0127-22.2023.f5-1 Extended Data Figure 5-1 Open-field travel trajectories of mice prestroke, post 1 and 7 d after stroke. Ten-minute open-field recording 24 h before stroke (green), 1 d poststroke (red), and 7 d poststroke (blue). Trajectories were smoother using Ramer–Douglas–Peucker algorithm (ε = 10). *Figure Contributions*: Tony Fong and Pankaj Gupta analyzed the data. Tony Fong created the figure. This extended data figure supports Figure 5. Download Figure 5-1, TIF file.

### Chronic tracking in custom home-cage

**Figure 6.**
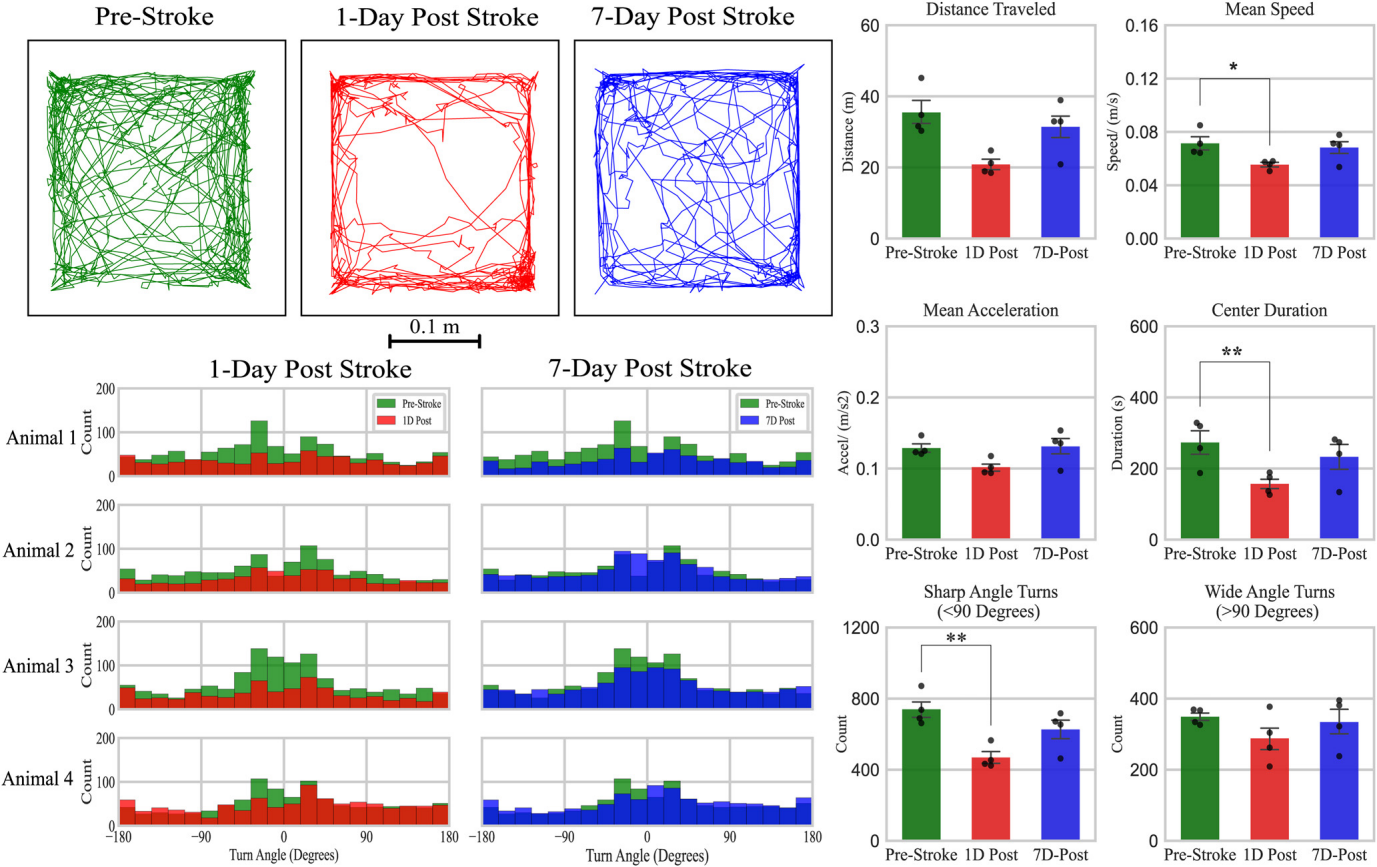
Analysis of 3-d behavior patterns in home-cage during day/night cycle (lights on and lights off). ***A***, Analysis of 3-d activity patterns. Total distance traveled by mice. *p* < 0.001; effect size = 0.69. Total duration of mice being active as detected by the motion detector. *p* < 0.001; effect size = 0.54. Number of sharp angle turns (<90°) made by mice. *p* < 0.001; effect size = 0.60. Number of wide angle turns (>90°) made by mice. *p* < 0.05; effect size = 0.20. Average speed of mice. *p* < 0.01; effect size = 0.29 vi) Average acceleration of mice detected. *p* > 0.05. ***B***, Analysis of 3-d activity patterns. Total duration spent in the center area of the cage, as defined by 0.5*width and 0.5*length around the center of the cage. No statistical significance was found in duration spent in the center, interacting with 1 mouse, nor interacting with two mice in daylight cycle. MANOVA was used with an ANOVA as *post hoc*. Data = mean ± SEM (*N* = 12). *p < 0.05, **p < 0.01, and ***p < 0.001. Each individual point represents a data point generated from a single mouse. *Figure Contributions*: Tony Fong performed the experiment, analyzed the data, and composed the figure.

A total of four cages each containing three mice were recorded. The effects of day/night cycle (lights on and lights off) on dependent outcomes such as distance traveled, active duration, average speed, wide angle turn count, sharp angle turn count, and average acceleration along with anxiety measures such as duration spent in the center, interacting with one or two mice were determined using an MANOVA (Seibenhener and Wooten, 2015). As expected, mouse activity levels follow a reverse circadian rhythm (Ananthasubramaniam and Meijer, 2020). Specifically, animals show higher levels of motor measures such as distance traveled (*p* < 0.001; effect size = 0.79), active duration (detected by the underlying motion detector; *p* < 0.001, effect size = 0.54), average speed (*p* < 0.01; effect size = 0.51), number of wide (>90°; *p* < 0.001; effect size = 0.62), and sharp (<90°; *p* < 0.001; effect size = 0.79) angle turns during periods of lights off as opposed to periods of lights on as shown in Figure 6*A*. However, no changes were observed in traditional anxiety related parameters such as duration spent in the center area, interacting with one or two mice as seen in Figure 6*B*.

### Social stimulus test

**Figure 7.**
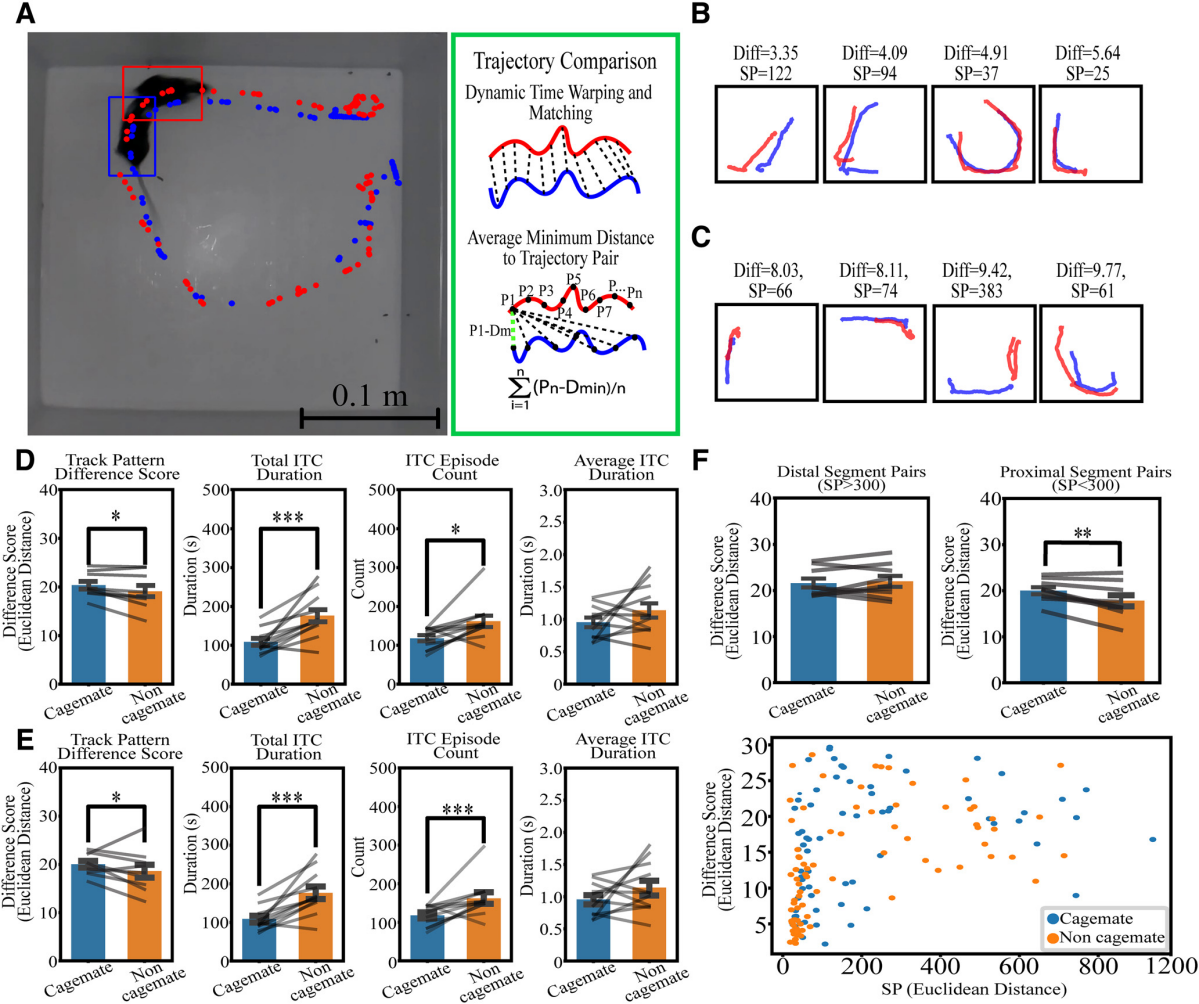
Travel trajectory analysis in an open-field social stimulus test. ***A***, The overall analysis of travel trajectory comparison. Travel trajectories from two mice were segmented into 5-s fragments. Segment pairs generated need to both have start-end displacement of >1 cm and to be similar in length in which one trajectory is not 35% longer or shorter than the other. If both criteria are met, the track pattern difference score and SP are generated. ***B***, Sample segment pair (4 lowest track pattern difference scores) comparison from noncagemates in a subject mouse. ***C***, Sample segment pair (4 lowest track pattern difference scores) comparison from cagemates in a subject mouse. ***D***, Social stimulus test result for 10 min. Average track pattern difference score, *p* < 0.05. Total interaction duration, *p* < 0.001. Number of social interaction episodes, *p* < 0.05. Average duration of each social interaction episode, *p* > 0.05 (*N* = 11 mice). ***E***, Social stimulus test result for the first 5 min of recording. Track pattern difference score (*p* < 0.05), total interaction duration (*p* < 0.001), number of social interaction episodes (*p* < 0.001), and average duration of each social interaction episode (*p* > 0.05) were found significant (*N* = 11 mice). ***F***, Track pattern difference score segregated by proximal (SP < 300) and distal (SP > 300) pairs. Track pattern difference score of distal segment pairs (*p* > 0.05) was not significant whereas, score of proximal segment pairs were (*p* < 0.01; *N* = 11 mice). Sample data in one test animal of all track pattern difference scores and SP values between segment pairs. Paired *t* test , *p < 0.05, **p < 0.01, and ***p < 0.001; data = mean ± SEM. Each individual point represents a data point generated from a single subject mouse. SP: Spatial Proximity. *Figure Contributions*: Tony Fong performed the experiment, analyzed the data, and composed the figure.

An advantage of PMT is the ability to track multiple animals simultaneously and in doing so can provide a report of potential social interactions. Analysis of mouse interactions could be important for assessing phenotypes related to mouse models of autism. Accordingly, we have evaluated tracks of multiple interacting mice to provide a method to approach these questions. Track pattern difference score, as its name implies, represents the similarity in pattern of the segmented pairs of travel trajectories regardless of length and spatial location. The larger the score would indicate a more dissimilarity in overall pattern between the trajectories. Therefore, comparison of identical trajectories would yield a value of 0. Spatial proximity is a measure of the distance/proximity between two trajectories; the lower the values, the closer the tracks are. Figure 7*B*,*C* illustrates sample trajectory comparisons of a single test mouse against a noncagemate and a cagemate, respectively. During the 10 min of recording, track pattern difference scores (*p* < 0.05), total ITC duration (*p* < 0.001), and number of ITC episodes (*p* < 0.05) were found to be statistically significant whereas average duration per ITC was not (*p* > 0.05) as seen in Figure 7*C*. For comparison to the more common 5 min of video recording used in the literature, the first 5 min was also separately analyzed as seen in Figure 7*D*, which resulted in similar results. Interestingly, the track pattern difference score is only statistically significant (*p* < 0.05) when comparing proximal segment pairs (SP < 300, proximal are potentially more similar) and not distal segment pairs (SP > 300) as shown in Figure 7*F*, segment pair comparison of an illustrative test animal to a cagemate and noncagemate stimulus animal.

## Discussion

**Figure 8.**
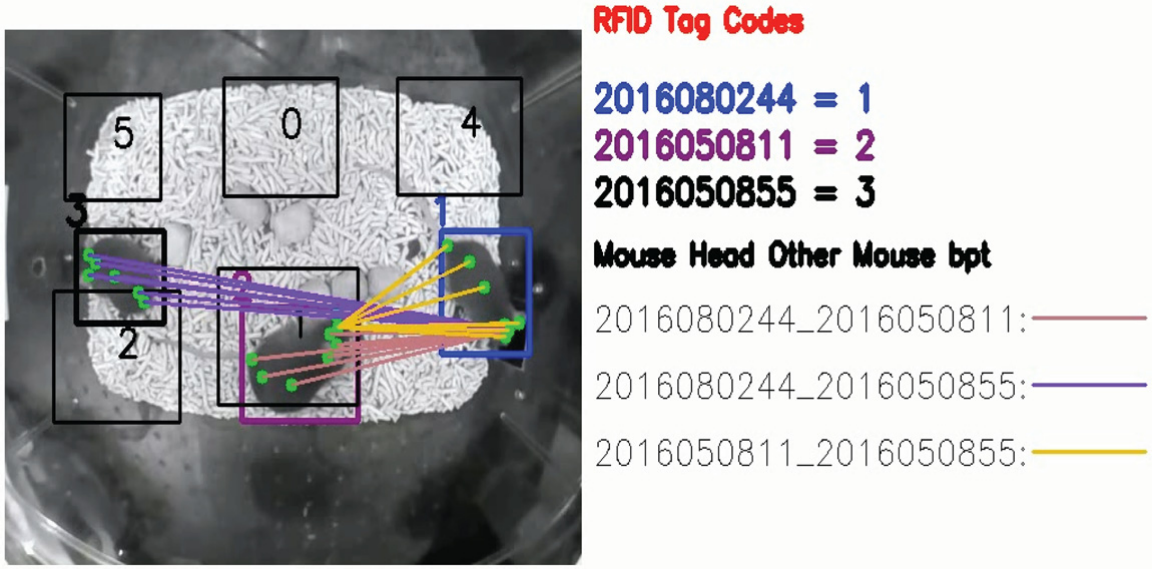
Incorporation of DeepLabCut posture estimation with PMT identity tracking. Body parts for each animal tracked include snout, head center, left ear, right ear, neck, mid body, lower mid body, and tail base. Distance of each mouse’s mouse head to other animal’s body parts are also shown. PMT: PyMouseTracks. *Figure Contributions*: Tony Fong performed the experiment, analyzed the data, and composed the figure.

Most automated animal assessment tools, while desirable, remain difficult to access for the broader neuroscience community because of costs, setup time, and ease of use. As of writing, live mouse tracker (LMT) is the only open-source RFID and video tracking system available (de Chaumont et al., 2019). PMT offers some advantages over LMT: (1) affordability, (2) scalability, (3) ease of setup/use, and (4) customizability. A PMT recording system with 6 RFID readers costs ∼520 USD. Affordability combined with PMT’s small footprint would enable investigators to implement and use at larger scales, for example, monitoring racks of mice in an animal facility. Most importantly, the key distinction of PMT is the ability to be customized for tracking different rodents (varied coat color or species) in varied housing environments. As more in-depth questions are asked, the complexity of experimental designs have also increased (Basso et al., 2021; Slivkoff and Gallant, 2021). Investigators need readily adaptable tools for their experiments involving rodents of different coat colors in different environments. As shown, PMT can be adapted to record and track mice in a variety of environments, other than our home-cage configuration. Most importantly, PMT can be retrained to recognize mice of different coat colors against different backgrounds. For more details on when and how to retrain, please to our documentation guide (Extended Data 1, 2, 3). At the same time, PMT tracking accuracy of darker coat-color mice is lower to that shown previously (de Chaumont et al., 2019) and currently unable to perform real-time tracking in exchange for the benefits mentioned above. Indeed, LMT can also further extrapolate postures and types of social interaction behaviors using depth information using depth sensing cameras (de Chaumont et al., 2019). However, PMT also has the capability to incorporate output of posture estimation from multianimal DeepLabCut (Lauer et al., 2022), which may enable similar features in the future (Fig. 8; Movie 10).

Movie 10. PMT Tracking with DeepLabCut posture estimation. PMT Tracking of mice in the modified home-cage with posture estimation input from multianimal DeepLabCut. Body parts for each animal tracked include snout, head center, left ear, right ear, neck, mid body, lower mid body, and tail base. Distance of animal head center to other mice’s body parts are also show. PMT: PyMouseTracks. *Video Contributions*: Tony Fong analyzed and created the video. 10.1523/ENEURO.0127-22.2023.video.10

Differences in PMT tracking performance were observed between our custom home-cage and open arena settings. The differences observed were likely related to two factors: bedding and the existence of an entrance area. In the home-cage, bedding acted as a physical barrier increasing the distance between the RFID tags and RFID readers, in turn, decreasing the likelihood of an RFID tag being read. Given that bedding tends to clump (as with the common aspen wood shavings), this can further exacerbate reader range error from our experience. When using a home-cage setting similar to ours, we recommend a pellet type bedding which is less likely to clump and easier to shift around. Regarding the entrance area, it can pose a source of false negatives, false positives, and identity errors, as it provides a space for mice to cluster and can complicate detection when mice enter or exit the cage with new IDs. As evident, better performance of tracking were observed in the open-field and three-chamber arena. Therefore, when using PMT to design their own experimental paradigms, investigators should take these two factors into account.

PMT has not only demonstrated capabilities to chronically track multiple mice, but also detect underlying animal phenotypes (stroke). Consistent with other studies, chronic recordings in the modified home-cage revealed diurnal locomotor activity patterns in mice (Wang et al., 2019; Ananthasubramaniam and Meijer, 2020). In an open-field arena, mice showed locomotor deficits immediately after stroke induction similar to other studies (Shenk et al., 2020; Shvedova et al., 2021). Similar to previous studies (Vahid-Ansari et al., 2016; Larpthaveesarp et al., 2021), stroke induced anxiety phenotype was also observed in mice as shown by less time spent in the center zone of the open-field. Interestingly, mice made a lower number of sharp angle turns (<90°) after stroke. A possible explanation for this is that high motor coordination between limbs is required to make precise turn angles. However, motor coordination is impaired because of stroke (Pollak et al., 2012; Wang et al., 2019), which lead to unsuccessful sharp angle turn attempts. Further validation of turn angle parameters may yield an assessor of motor coordination in open-field paradigms.

Social function deficits are key symptoms in many psychiatric disorders including autism, depression, and schizophrenia (Benekareddy et al., 2018; Fujikawa et al., 2022). Currently, the most widely used test in rodents is the three-chamber sociability and social preference assay (Golden et al., 2011; Kim et al., 2019).The main outcome of interest is the duration spent by the subject mouse in a chamber containing a stranger (noncagemate) mouse compared with that in a chamber of a familiar (cagemate) mouse, both of which are confined by a wired cage. In general, mice would have a higher preference in investigating a stranger mouse compared with a familiar mouse as measured by the duration spent together and number of approaches (Golden et al., 2011; Kim et al., 2019; Beery and Shambaugh, 2021). However, mouse social interactions are complex and affected by the states of both participating parties presenting the possibility that confinement of a mouse may risk reducing ecological validity. PMT would allow the investigation of social preference in freely moving mice pairs. Indeed, mice still tend to have an increased duration of total interaction time and number of interactions with a stranger mouse compared with a familiar mouse. Moreover, we applied dynamic time warping, a method common in travel trajectory pattern comparisons (Brankovic et al., 2020), to compare similarities of trajectory patterns of a subject animal to that of a stranger or familiar mouse. Interestingly, it was found that travel trajectories of noncagemate pairs tend to be similar when in proximity compared with that of cagemate pairs. Therefore, the comparison of travel trajectory patterns may offer a novel method in determining sociability of mice pairs that could provide an adjunct to the 3-chamber sociability test.

Overall, we have demonstrated the effectiveness of the PMT for tracking visually different rodents in a range of experimental settings and have shown the system’s ability to detect pathologic changes in motor kinetics induced by stroke. We hope that this tool can enable the use of more complex experimental paradigms. In our online repository and guide, we provide detailed instructions to setup and use PMT’s online data collection and offline analysis software. In the future, we hope to further improve PMT based on the community’s feedback.

## Synthesis

Reviewing Editor: William Stacey, University of Michigan Decisions are customarily a result of the Reviewing Editor and the peer reviewers coming together and discussing their recommendations until a consensus is reached. When revisions are invited, a fact-based synthesis statement explaining their decision and outlining what is needed to prepare a revision will be listed below. The following reviewer(s) agreed to reveal their identity: Alex Legaria, vivek kumar.

The reviewers and I agree that this open source software is an important addition, and one of the reviewers was able to verify that the code functions. You have responded to most of the previous comments. However, there remain several significant concerns about the quality of the writing and presentation. Reviewers commented that the revision focused on the specifics of the previous review, but did not attempt to make the writing quality better. At present this is not of sufficient quality for presentation. I suggest not only responding to the specifics below, but have the entire paper reviewed by a colleague not involved with the project to provide feedback on presentation and organization.

1. In the manuscript there are four supplemental figures mentioned in the figure legends (3-1, 3-1, 3-3, 6-1) but only one is shown (supplemental 3).

2. The previous comment about y-axis in figure 4, while it was reportedly addressed in the methods, still stands. The y-label of figure 4 still says that it is a percentage of mice (% Mice ground truth), however no percentage is being calculated and it may be misleading if kept as is. While this is a small detail, it is concerning that it was not fixed.

3. The sample_us1.ipynb is not properly commented and not very readable.

4. The RFID capsule (SEN-09416) is not designed to be implanted. this is clearly stated by the manufacturer. It is unclear how this managed to get by IACUC.

5. This is a methods paper but the methods are split between results and methods. It makes the narration difficult to follow. Figures 1 and 3 are interrupted by figure 2. These are the data acquisition system (computational) and mouse apparatus. They should be together.

6. Figures 5 and 6 are misnumbered or incorrectly referred to in the text.

7. For Neural Network Training, please confirm that videos were held out for validation, not individual frames. Its important to train and test on different videos for generalizability. This is important!

8. The methods are vague, in the results it states that a new network was trained for FVB (white mice). However, in the methods this is not clearly stated. The reader show not have to search for these metrics.

9.. Please clearly state the number of images for each strain, training images should also be made available for others to use. They can be shared on Zenodo for instance.

10. The number of videos and length are often not clearly stated -

11. Figure 4 - the metrics are poorly explained. What does it mean in A - total deletion Count ^3?

12. False positive % mice ground truth - I’m not clear on what is meant. The text is very hard to understand.

13. Figure 5 - please present behavioral patterns in the following way. These are routine in the circadian field. 1. Continuous data for 3 days. 2. Wave form data over 24 hours

14. What is the rationale for looking at angle of turns in stroke and 24hr monitoring?

15. How is the degree of turn defined?

16. Figure 6B - are there really 12,000 sharp angle turns in a 10 minute open field video?

17. The tracking data in 6A shows a dramatic difference between pre and 1d post stroke (red/blue). However, the total distance does not show any mouse with a dramatic difference.

18. Why switch colors from red/blue (tracking) to blue/yellow (bar plot), to green/brown (angle detain B). It just makes for a hard to read figure.

19. The text in figure 3 (numbers in the arena) is hard to read and the legend does not explain what they are

20. Similarly figure 4C has a lot of unnecessary text not the images.

21. Figure 2A (right most figure) has numbers for RFID sensor, bit these are never explained in the legend.

22. Figure 7 - what is the rationale for this figure? What is it meant to add to the paper describing this tracking system? The data is simply presented without any rationale.

21. The reference for pattern matching is missing Meert 2020.

22. Please refer to the source of your mice C57BL/6J for JAX as an example, add stock number.

21. FVB/N is misspelled on line 290

22. The order of the methods should be fixed - for instance stroke induction is followed Manual validation if followed by social stimulus test. Suggest all ML/stats methods in one section followed by animal tests. Clearly state the number of animals, length of videos, training/test splits, Frame rate etc.

23. Line 266 “Fig 4A shows reliability of the pipeline” - how exactly does it show this? See point above.

24. Stats in lines 311-312 - please state pvalue after anova F stats.

25. Chronic tracking section is very poorly described. the figure has 9 panels but only two labels (A and B) this make it hard to follow and it’s very sparsely described in the text.

## Author Response

Reviewer 1

1. Starting from the abstract, it was stated that “PRT maintained a minimum of 88% detections tracked with an overall accuracy >85% when compared to manual validation of videos containing 1-4 mice in a modified home-cage.” However, 88% or 85% were never mentioned in the rest of the manuscript

Response: We have added additional descriptions in the results indicating that the 88% coverage and 85% accuracy were the minimal achieved among our validation set.

We added the following: ’In our validation set, the minimal value achieved for detection coverage and identity accuracy were 88% and 85%, respectively.’

2. Page 5. Line 72. Key component RFID reader SparkFun part number SEN-00963 is incorrect (this part number does not exist).

Response: We have made the correction from SEN-00963 to SEN-09963 (line 71-72)

3. Line 75, the waveshare SKU is a OV5647 camera. It is not the official Pi V1 camera. The documentation link provided in the manuscript may apply but these two cameras may also be different.

Response: Yes, we agree that the cameras are not exactly same despite that the documentation would be the same when used with a raspberry pi. We have added ’a Rpi V1 camera equivalent’ to the description.

4. Line 91. A tensorflow 2 framework of Yolov4 recognized by ... The sentence does not make sense. The citation needs to be fixed.

Response: We have made the changes accordingly and corrected the citation.

‘The pipeline contains three components as seen in Fig 1B and Fig 2A: 1) Yolov4 for mouse detections (Bochkovskiy et al., 2020), 2) modified SORT for mice tracking (Bewley et al., 2016), 3) RFID to SORT ID matching for identity confirmation. Mice are first detected by an official TensorFlow 2 framework of Yolov4 (Bochkovskiy et al., 2020; Hùng, 2021)’.

5. Line 89. Modified SORT: please describe what modification was done.

Response: We have emphasized the changes made to SORT on line 92-97.

‘Two features were added to the SORT algorithm including the Kalman filter predictions during track lost and SORT-ID reassociation to enhance tracking performance, forming the modified SORT. As the maximum number of mice is known, the Kalman filter can therefore predict mouse position when Yolov4 fails, such as in cases of visual occlusions or detections in proximity. Similarly, new false positive IDs can be associated with a previous ID that disappeared. At times, new IDs will be generated for the same mouse, discarding a previous tracked ID. The generation of these new false SORT-IDs is due to the irregular travel patterns taken by mice which remains difficult to completely be predicted by SORT’s underlying Kalman filter.’

6. Line 120. Training data and training procedure should be provided so that users who need to train their own weights (as suggested by the authors) can follow the steps.

Response: All data has been uploaded to Open Science Framework (OSF) including data analyzed and used to train the network for mouse detection. We have also included a manual 2 for using PRT which includes steps to train new weights for detecting mice. Moreover, we have created a docker file and google colab notebook for users to train their weights in. Both the docker file and colab notebook is in our GitHub.

7. Line 132. Three-day chronic recording is misleading. It was several 10-minute recordings and not a continuous 72-hour recording. The use of Fisheye camera is interesting but has the effect of image distortion caused by fisheye been tested on YOLO? What is the angle of view of the fisheye?

Response: Although the recording was not continuous, the three-day chronic recording is not several 10-minute recording. Recordings were on average 12 hours with a 1 second restart delay. Although we have not tested the effects of the fisheye lens on Yolo, we did not see an effect of the fisheye lens onYolo accuracy as the network was still able to achieve >99% mean average prevision (mAP) in training on the validation set. The angle of view is 160 degree and has been added to the descriptions.

8. The title is PyrodentTracks but the system is only tested in mice. Rats are much larger and there might be difficulties the authors did not encounter. (e.g. with longer legs, the RFID, even embedded in the abdomen, maybe too far away to be detected by the RFID reader, especially when there is bedding).

Response: Based on your comments we are decided to change the title to PyMouseTracks.

9. Line 182. Small incisions were made in the lower abdomen but RFID was injected below the nape of the neck? This is critical description. Distance is critical for RFID sensitivity.

Response: Correction and clarifications were made. In all cases RFID implants were made to in the lower abdomen for all Bl6 mice, in 3 white mice we injected the tag in the nape of neck.

‘...,a small incision was made below the nape of the neck or in the lower abdomen, depending on RFID placement. A sterile injector (Fofia, ZS006) was then used to insert the RFID capsule subcutaneously below the nape of the neck or at the abdomen (abdominal will yield better performance). ’

10. Why are different video size chosen? 512x400, 960x960, 640x480, etc. These seem to be chosen at random. How does this affect performance?

Response: Yes, they were indeed random because we want to demonstrate that different resolution input images can still be used for inference. For this range of resolution there was no effect of video size on performance was noticed. Generally speaking, increasing the resolution parameter will increase accuracy.

11. FN(t). Should this be FN(f)? What is (t)? It was never explained. If I can’t trust the formula (actually the only formula), I will have doubts on the rest of the data.

Response: The corrections have been made. F represents frame. 3 MOTA = 1 - sum(f) (FN (f) + FP(f) + identity error(f)) / sum(f) (number of mice in the ground truth)

12. Line 243. Stats. RM-ANOVA should be followed by post-hoc those controls family-wise error rates. Paired- Students t-tests is not one of them. Also, when reporting ANOVA results, please include F and df.

Response: We have updated the description of the Student’s t-test used which included Bonferroni correction. We have also included Df and F for the RM-ANOVA used. (Line p15-16;304-331)

13. Video on setup software. After cloning, “chmod 777” is not the best thing to do. All you need is “chmod 711”, not need to give everyone write access to your code on the production box.

Response: We have made changes to the video to include the option of chmod 777 and chmod 711.

14. Line 249: why name your code in the extended data “video”?

Response: We did not observe the mistake mentioned

15. Line 275: LMT only uses one video to conduct MOTA. PRT uses video and RFID for tracking, but LMT archives better performance? BTW, the MOTA value for 1-3 mice going from 0.998, 0.945 and 0.973 appears to be strange. Although the difference is small, this should scale more linearly.

Response: Normally, indeed it should be scaling more linearly, if in a complete open field. However, because of the small size of the cage and the existence of the entrance area as explained in discussion, the abnormal scaling depending on rodent activity, i.e. access to the entrance area, could be expected as described in the discussion or just reflecting noise. At the same time, this might mean that the number of mice is not a significant factor for MOTA in the demonstrated scenarios.

16. Line 280 bl6 black mice. Please report the official name of the mice.

Response: Changes were made to C57BL/6.

17. Why retraining? Please describe when retraining is needed and how to train.

Response: In general, retraining should be done if the environment (and mouse coat color) differs a lot from our use case. We have described a method in our documentation to check for the accuracy (expressed in mAP, false positives, false negatives, and true positives) of detections produced by Yolov4 weights against user defined labels. From our observations, we recommend retraining if MOTA values for a given weight falls below 0.95 against user defined labels.

18. The stroke experiment is puzzling. This does not appear to need tracking of multiple mice.

Any conventional open field software should be able to achieve the same results.

Response: Here, we wanted to demonstrate the effectiveness of PRT in detecting motor impairments. In the future, we will apply the use case to home-cage environments with multiple mice in a more longitudinal framework. 19. Line 317: wide and sharp: missing the word angle 4

Response: We have made the corrections

20. Line 320. Social interaction remains similar between lights on and off? It is strange. Doe this indicate something could be wrong with your experiment or your algorithm.

Response: Statistically, we did not observe a difference and we believe that small size of the cage would be the main reason for as there is limited space for movement. However, we did observe a trend for a mouse to have longer durations of interaction with two animals during lights on periods which may be indicative of clumping behavior during sleep.

21. During in center of arena is a measure of anxiety when the arena is novel, and the arena is sufficiently large. The home cage setup is not such an environment

Response: We agree that the main center area may not reflect anxiety in a homecage setting.

The if the center as an region of interest is mainly to demonstrate the capabilities of the offline analysis package. In the future, we will employ a larger and more interconnected cage environment for testing taking advantage of PRT flexibility.

22. Coat color is not a criterion used in PRT.

Response: We have indeed used a lighter coat-colored mice for tracking the three chamber and describe how to re-train models to accommodate this in our manual and GitHub page.

23. Why not put an RFID directly under the entrance to capture the enter and exiting events?

Response: We have put an RFID reader under the entrance, perhaps this was not clear.

‘A reader is attached underneath the tunnel leading to the mouse housing area.’

24. Table includes the RFID breakout board, not the actual reader with USB connection.

Response: We have made a correction to the naming of the part to breakout board (Sparkfun; SEN-1303) in text as well. The component does contain a (micro)USB connection. Together with the antenna (IDL20A), it forms the complete RFID reader component.

25. Figure 2: B. Yolo should be able to recognize multiple mice in the two or three mice images if you trained it well.

Response: The accuracy of Yolo to detect multiple mice in proximity depends on a user defined setting of intersection over union (IOU) between detections. Similar to other detection algorithms, Yolov4 will create multiple detections, in the form of bounding boxes, for the same object in an image. Based on the IOU of the detections generated a non-mx suppression algorithm is used to determine the true detection for an object by merging bounding boxes with the set IOU limit. If multiple mice are too close in proximity, their bounding boxes will have a high IOU value leading to only one detection. Hence, we have implemented our modified SORT algorithm to recognize multiple mice in proximity.

Reviewer 2

1. The y-axis in the False Positive and False Negative rates is “% Mice Ground Truth.”

However, it is unclear what this means. Is it a percentage of all the detected counts (Figure 1A)? Or is it really the percentage of mice. It is unlikely that it is the latter, given the values of the y-axis. While there is a short description of False Positive and False Negatives in the

“Manual Validation of Videos” section of the methods, we find this explanation insufficient.

Thus, it is critical for publication to have a better description of the validation methods for multiple-mice tracking. 5

Response: We have updated our manuscript to include a more detailed description of validation methods. The “Mice Ground Truth.” is the total sum of mice in each frame of a video. This is calculated by the total number of detections in Figure 1A plus total false negatives and then subtracted by total false positives. Total false negatives and total false positives were determined by student evaluators looking each videos frame by frame.

2. Lack of documentation: While one of the strengths of PyRodentTracks is supposed to be that it is plug-and-play, the lack of documentation of both how to put together the device and how to install and use the offline data analysis pipeline is a little concerning. The GitHub and associated Youtube videos though helpful, are by no means sufficient to understand the data analysis pipeline process.

Response: We have now included a detailed written manual for the offline data analysis, including installation and use. Video guides are also included. Moreover, we have also included a docker for user to retrain the weights for Yolov4. For less technical users, we have included google colab notebooks to use and train the offline analysis in the cloud.

3. Confusing methods: The “PRT Offline Analysis Pipeline”, “Home-Cage Recording Setup AND PRT Pipeline Line Adjustments”, “Motion Detection and Trajectory Analysis” sections of the methods are confusing. More specifically, the description of the ID to Tag matching could be described more clearly, or at least a better visualization is needed (the schematics in Figure 2C are not very helpful.

Response: We have made edits and color adjustments to Figure 2C to better explain the offline analysis pipeline.

4. Supplemental Figure 1 needs better labels, bigger font, and a better description of the data.

Response: Supplemental figure 1 has been split in to multiple figure for better visual visualization. The raw images of the charts cannot be changed as they were generated as png files directly from darknet when training.

5. Explain why there is a drift in Figure 4C bottom (yellow tracking) if all the mice were being tracked at the same time?

Response: We have corrected the drift. The drift was induced when compiling and editing the figure in adobe premium.
