# Supplementary figures and images for: PyMouseTracks: Flexible Computer Vision and RFID-Based System for Multiple Mouse Tracking and Behavioral Assessment
Source: eNeuro. 2023 May 12;10(5):ENEURO.0127-22.2023. doi: 10.1523/ENEURO.0127-22.2023 (PMC10198609; doi:10.1523/ENEURO.0127-22.2023)

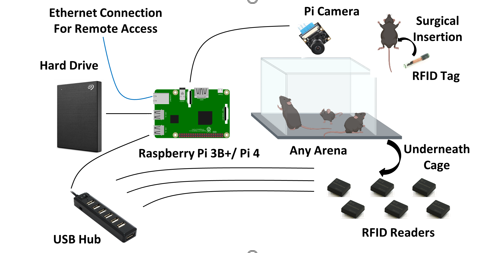

Supplement: Extended Data 1. — PMT online data collection module. Complete software package for online data recording. The package should be installed on a RPi microcomputer. Figure Contributions: Tony Fong wrote, tested the software, analyzed, and created the video. This extended data file supports Figure 1. Download Extended Data 1, ZIP file. [file enu-eN-MNT-0127-22-s16.zip › tracker_rpi-main/concept.png]

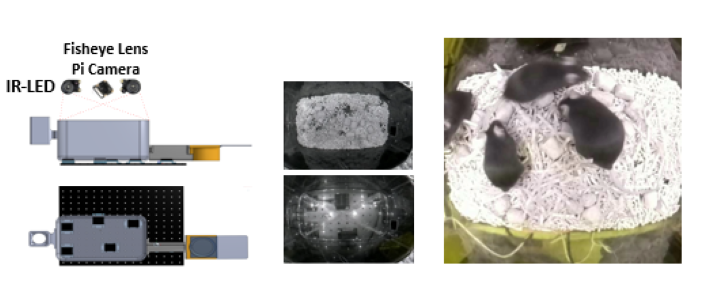

Supplement: Extended Data 1. — PMT online data collection module. Complete software package for online data recording. The package should be installed on a RPi microcomputer. Figure Contributions: Tony Fong wrote, tested the software, analyzed, and created the video. This extended data file supports Figure 1. Download Extended Data 1, ZIP file. [file enu-eN-MNT-0127-22-s16.zip › tracker_rpi-main/home_cage_example.PNG]

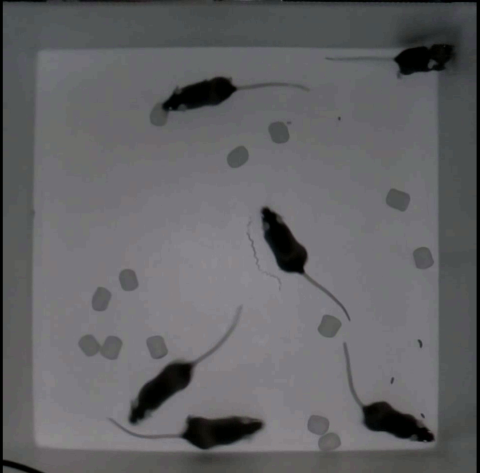

Supplement: Extended Data 1. — PMT online data collection module. Complete software package for online data recording. The package should be installed on a RPi microcomputer. Figure Contributions: Tony Fong wrote, tested the software, analyzed, and created the video. This extended data file supports Figure 1. Download Extended Data 1, ZIP file. [file enu-eN-MNT-0127-22-s16.zip › tracker_rpi-main/open_field.PNG]

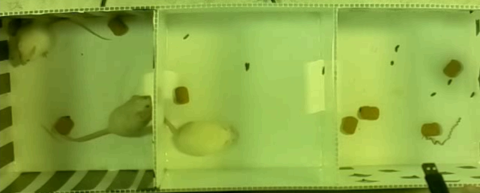

Supplement: Extended Data 1. — PMT online data collection module. Complete software package for online data recording. The package should be installed on a RPi microcomputer. Figure Contributions: Tony Fong wrote, tested the software, analyzed, and created the video. This extended data file supports Figure 1. Download Extended Data 1, ZIP file. [file enu-eN-MNT-0127-22-s16.zip › tracker_rpi-main/three_chamber.PNG]

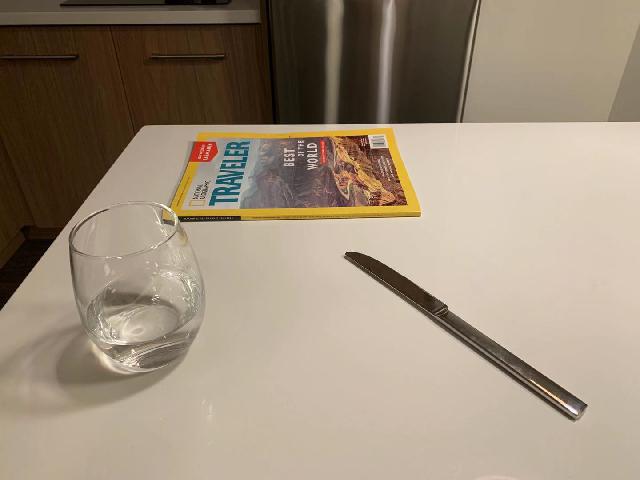

Supplement: Extended Data 2. — PMT offline data analysis module. Complete software package for offline data analysis. The package was installed and ran in an Anaconda environment (https://www.anaconda.com). Code was tested on a Windows 10 PC (AMD Ryzen 7 5800X; 64 GB; RTX 2080Ti) and Linux (Ubuntu 20.04.2 LTS) PC (Intel i7-7800X; 94 GB; GTX Titan X). Figure Contributions: Tony Fong wrote and tested the software. This extended data file supports Figure 1. Download Extended Data 2, ZIP file. [file enu-eN-MNT-0127-22-s17.zip › ED2 PMT Offline Data Analysis Module/PRT/android/app/src/androidTest/assets/table.jpg]

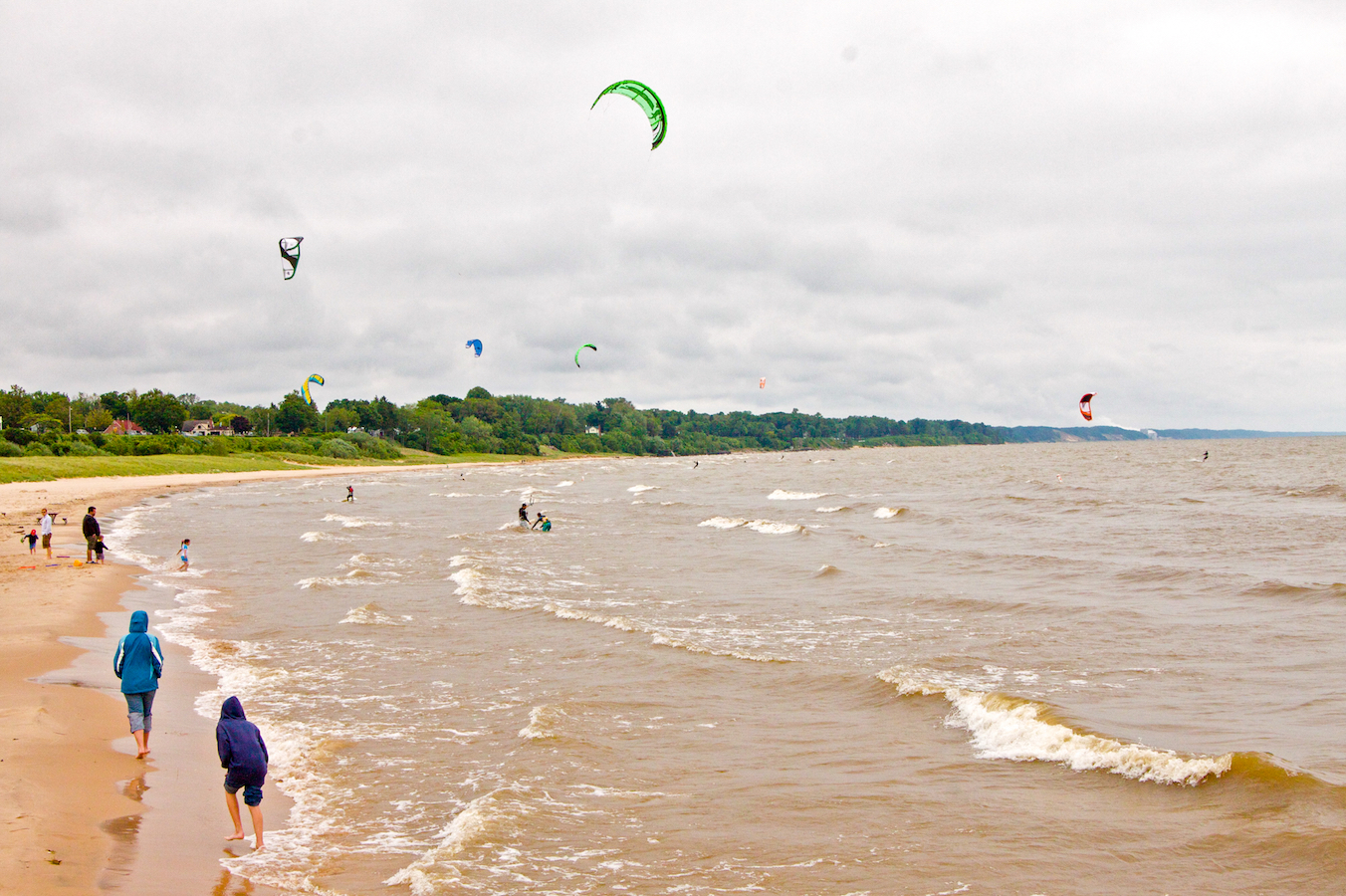

Supplement: Extended Data 2. — PMT offline data analysis module. Complete software package for offline data analysis. The package was installed and ran in an Anaconda environment (https://www.anaconda.com). Code was tested on a Windows 10 PC (AMD Ryzen 7 5800X; 64 GB; RTX 2080Ti) and Linux (Ubuntu 20.04.2 LTS) PC (Intel i7-7800X; 94 GB; GTX Titan X). Figure Contributions: Tony Fong wrote and tested the software. This extended data file supports Figure 1. Download Extended Data 2, ZIP file. [file enu-eN-MNT-0127-22-s17.zip › ED2 PMT Offline Data Analysis Module/PRT/android/app/src/main/assets/kite.jpg]

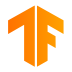

Supplement: Extended Data 2. — PMT offline data analysis module. Complete software package for offline data analysis. The package was installed and ran in an Anaconda environment (https://www.anaconda.com). Code was tested on a Windows 10 PC (AMD Ryzen 7 5800X; 64 GB; RTX 2080Ti) and Linux (Ubuntu 20.04.2 LTS) PC (Intel i7-7800X; 94 GB; GTX Titan X). Figure Contributions: Tony Fong wrote and tested the software. This extended data file supports Figure 1. Download Extended Data 2, ZIP file. [file enu-eN-MNT-0127-22-s17.zip › ED2 PMT Offline Data Analysis Module/PRT/android/app/src/main/res/drawable-hdpi/ic_launcher.png]

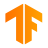

Supplement: Extended Data 2. — PMT offline data analysis module. Complete software package for offline data analysis. The package was installed and ran in an Anaconda environment (https://www.anaconda.com). Code was tested on a Windows 10 PC (AMD Ryzen 7 5800X; 64 GB; RTX 2080Ti) and Linux (Ubuntu 20.04.2 LTS) PC (Intel i7-7800X; 94 GB; GTX Titan X). Figure Contributions: Tony Fong wrote and tested the software. This extended data file supports Figure 1. Download Extended Data 2, ZIP file. [file enu-eN-MNT-0127-22-s17.zip › ED2 PMT Offline Data Analysis Module/PRT/android/app/src/main/res/drawable-mdpi/ic_launcher.png]

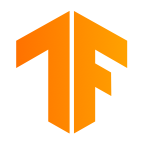

Supplement: Extended Data 2. — PMT offline data analysis module. Complete software package for offline data analysis. The package was installed and ran in an Anaconda environment (https://www.anaconda.com). Code was tested on a Windows 10 PC (AMD Ryzen 7 5800X; 64 GB; RTX 2080Ti) and Linux (Ubuntu 20.04.2 LTS) PC (Intel i7-7800X; 94 GB; GTX Titan X). Figure Contributions: Tony Fong wrote and tested the software. This extended data file supports Figure 1. Download Extended Data 2, ZIP file. [file enu-eN-MNT-0127-22-s17.zip › ED2 PMT Offline Data Analysis Module/PRT/android/app/src/main/res/drawable-xxhdpi/ic_launcher.png]

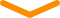

Supplement: Extended Data 2. — PMT offline data analysis module. Complete software package for offline data analysis. The package was installed and ran in an Anaconda environment (https://www.anaconda.com). Code was tested on a Windows 10 PC (AMD Ryzen 7 5800X; 64 GB; RTX 2080Ti) and Linux (Ubuntu 20.04.2 LTS) PC (Intel i7-7800X; 94 GB; GTX Titan X). Figure Contributions: Tony Fong wrote and tested the software. This extended data file supports Figure 1. Download Extended Data 2, ZIP file. [file enu-eN-MNT-0127-22-s17.zip › ED2 PMT Offline Data Analysis Module/PRT/android/app/src/main/res/drawable-xxhdpi/icn_chevron_down.png]

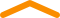

Supplement: Extended Data 2. — PMT offline data analysis module. Complete software package for offline data analysis. The package was installed and ran in an Anaconda environment (https://www.anaconda.com). Code was tested on a Windows 10 PC (AMD Ryzen 7 5800X; 64 GB; RTX 2080Ti) and Linux (Ubuntu 20.04.2 LTS) PC (Intel i7-7800X; 94 GB; GTX Titan X). Figure Contributions: Tony Fong wrote and tested the software. This extended data file supports Figure 1. Download Extended Data 2, ZIP file. [file enu-eN-MNT-0127-22-s17.zip › ED2 PMT Offline Data Analysis Module/PRT/android/app/src/main/res/drawable-xxhdpi/icn_chevron_up.png]

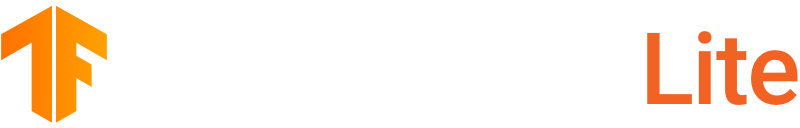

Supplement: Extended Data 2. — PMT offline data analysis module. Complete software package for offline data analysis. The package was installed and ran in an Anaconda environment (https://www.anaconda.com). Code was tested on a Windows 10 PC (AMD Ryzen 7 5800X; 64 GB; RTX 2080Ti) and Linux (Ubuntu 20.04.2 LTS) PC (Intel i7-7800X; 94 GB; GTX Titan X). Figure Contributions: Tony Fong wrote and tested the software. This extended data file supports Figure 1. Download Extended Data 2, ZIP file. [file enu-eN-MNT-0127-22-s17.zip › ED2 PMT Offline Data Analysis Module/PRT/android/app/src/main/res/drawable-xxhdpi/tfl2_logo.png]

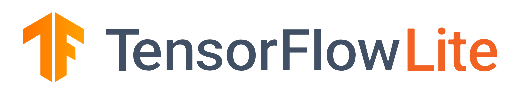

Supplement: Extended Data 2. — PMT offline data analysis module. Complete software package for offline data analysis. The package was installed and ran in an Anaconda environment (https://www.anaconda.com). Code was tested on a Windows 10 PC (AMD Ryzen 7 5800X; 64 GB; RTX 2080Ti) and Linux (Ubuntu 20.04.2 LTS) PC (Intel i7-7800X; 94 GB; GTX Titan X). Figure Contributions: Tony Fong wrote and tested the software. This extended data file supports Figure 1. Download Extended Data 2, ZIP file. [file enu-eN-MNT-0127-22-s17.zip › ED2 PMT Offline Data Analysis Module/PRT/android/app/src/main/res/drawable-xxhdpi/tfl2_logo_dark.png]

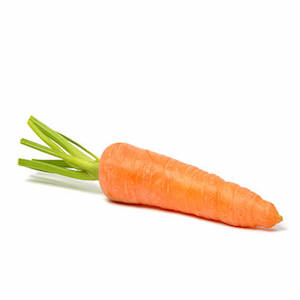

Supplement: Extended Data 2. — PMT offline data analysis module. Complete software package for offline data analysis. The package was installed and ran in an Anaconda environment (https://www.anaconda.com). Code was tested on a Windows 10 PC (AMD Ryzen 7 5800X; 64 GB; RTX 2080Ti) and Linux (Ubuntu 20.04.2 LTS) PC (Intel i7-7800X; 94 GB; GTX Titan X). Figure Contributions: Tony Fong wrote and tested the software. This extended data file supports Figure 1. Download Extended Data 2, ZIP file. [file enu-eN-MNT-0127-22-s17.zip › ED2 PMT Offline Data Analysis Module/PRT/android/app/src/main/res/drawable-xxxhdpi/caret.jpg]

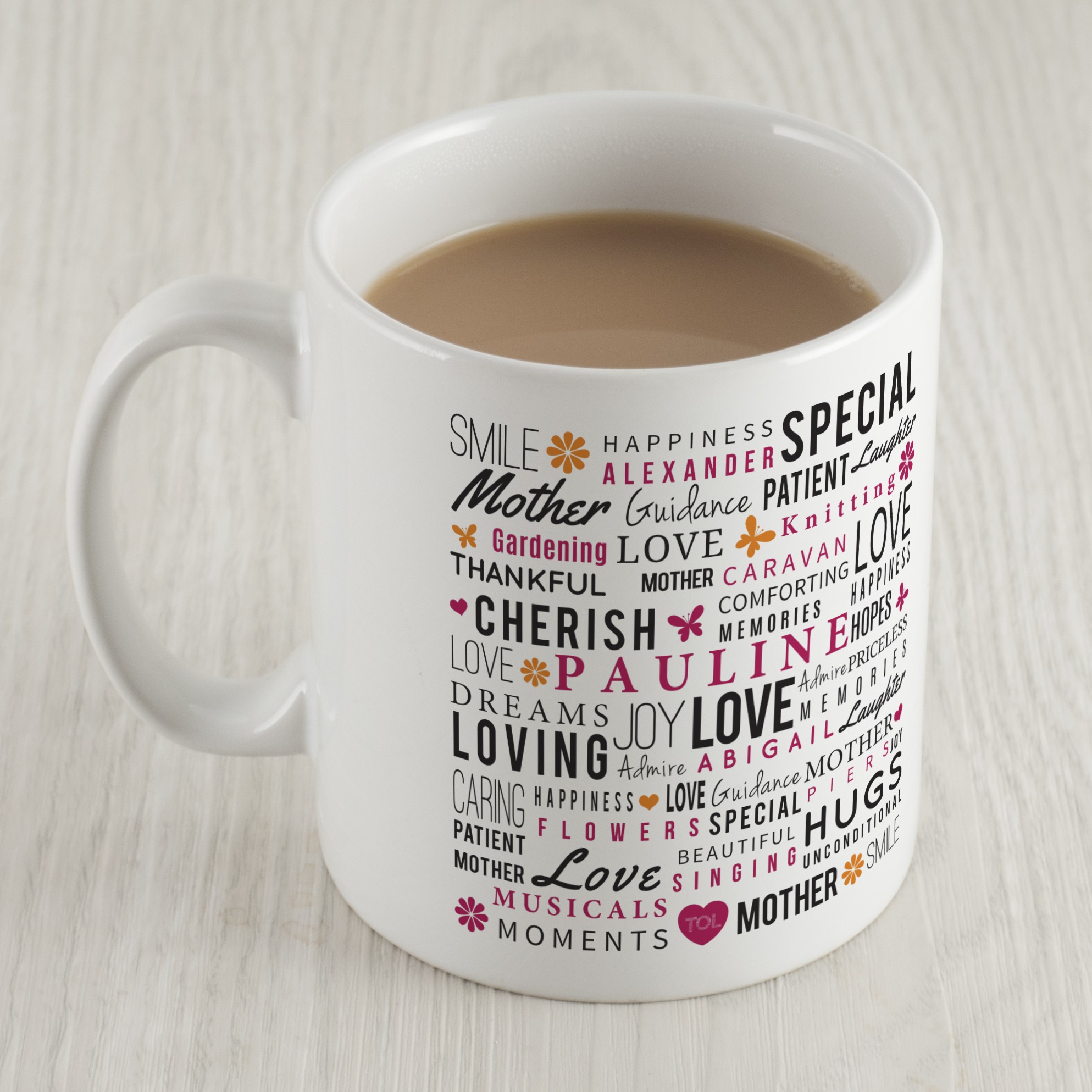

Supplement: Extended Data 2. — PMT offline data analysis module. Complete software package for offline data analysis. The package was installed and ran in an Anaconda environment (https://www.anaconda.com). Code was tested on a Windows 10 PC (AMD Ryzen 7 5800X; 64 GB; RTX 2080Ti) and Linux (Ubuntu 20.04.2 LTS) PC (Intel i7-7800X; 94 GB; GTX Titan X). Figure Contributions: Tony Fong wrote and tested the software. This extended data file supports Figure 1. Download Extended Data 2, ZIP file. [file enu-eN-MNT-0127-22-s17.zip › ED2 PMT Offline Data Analysis Module/PRT/android/app/src/main/res/drawable-xxxhdpi/sample_image.jpg]

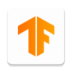

Supplement: Extended Data 2. — PMT offline data analysis module. Complete software package for offline data analysis. The package was installed and ran in an Anaconda environment (https://www.anaconda.com). Code was tested on a Windows 10 PC (AMD Ryzen 7 5800X; 64 GB; RTX 2080Ti) and Linux (Ubuntu 20.04.2 LTS) PC (Intel i7-7800X; 94 GB; GTX Titan X). Figure Contributions: Tony Fong wrote and tested the software. This extended data file supports Figure 1. Download Extended Data 2, ZIP file. [file enu-eN-MNT-0127-22-s17.zip › ED2 PMT Offline Data Analysis Module/PRT/android/app/src/main/res/mipmap-hdpi/ic_launcher.png]

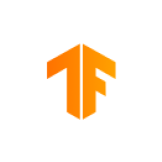

Supplement: Extended Data 2. — PMT offline data analysis module. Complete software package for offline data analysis. The package was installed and ran in an Anaconda environment (https://www.anaconda.com). Code was tested on a Windows 10 PC (AMD Ryzen 7 5800X; 64 GB; RTX 2080Ti) and Linux (Ubuntu 20.04.2 LTS) PC (Intel i7-7800X; 94 GB; GTX Titan X). Figure Contributions: Tony Fong wrote and tested the software. This extended data file supports Figure 1. Download Extended Data 2, ZIP file. [file enu-eN-MNT-0127-22-s17.zip › ED2 PMT Offline Data Analysis Module/PRT/android/app/src/main/res/mipmap-hdpi/ic_launcher_foreground.png]

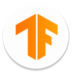

Supplement: Extended Data 2. — PMT offline data analysis module. Complete software package for offline data analysis. The package was installed and ran in an Anaconda environment (https://www.anaconda.com). Code was tested on a Windows 10 PC (AMD Ryzen 7 5800X; 64 GB; RTX 2080Ti) and Linux (Ubuntu 20.04.2 LTS) PC (Intel i7-7800X; 94 GB; GTX Titan X). Figure Contributions: Tony Fong wrote and tested the software. This extended data file supports Figure 1. Download Extended Data 2, ZIP file. [file enu-eN-MNT-0127-22-s17.zip › ED2 PMT Offline Data Analysis Module/PRT/android/app/src/main/res/mipmap-hdpi/ic_launcher_round.png]

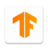

Supplement: Extended Data 2. — PMT offline data analysis module. Complete software package for offline data analysis. The package was installed and ran in an Anaconda environment (https://www.anaconda.com). Code was tested on a Windows 10 PC (AMD Ryzen 7 5800X; 64 GB; RTX 2080Ti) and Linux (Ubuntu 20.04.2 LTS) PC (Intel i7-7800X; 94 GB; GTX Titan X). Figure Contributions: Tony Fong wrote and tested the software. This extended data file supports Figure 1. Download Extended Data 2, ZIP file. [file enu-eN-MNT-0127-22-s17.zip › ED2 PMT Offline Data Analysis Module/PRT/android/app/src/main/res/mipmap-mdpi/ic_launcher.png]

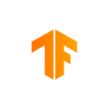

Supplement: Extended Data 2. — PMT offline data analysis module. Complete software package for offline data analysis. The package was installed and ran in an Anaconda environment (https://www.anaconda.com). Code was tested on a Windows 10 PC (AMD Ryzen 7 5800X; 64 GB; RTX 2080Ti) and Linux (Ubuntu 20.04.2 LTS) PC (Intel i7-7800X; 94 GB; GTX Titan X). Figure Contributions: Tony Fong wrote and tested the software. This extended data file supports Figure 1. Download Extended Data 2, ZIP file. [file enu-eN-MNT-0127-22-s17.zip › ED2 PMT Offline Data Analysis Module/PRT/android/app/src/main/res/mipmap-mdpi/ic_launcher_foreground.png]

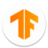

Supplement: Extended Data 2. — PMT offline data analysis module. Complete software package for offline data analysis. The package was installed and ran in an Anaconda environment (https://www.anaconda.com). Code was tested on a Windows 10 PC (AMD Ryzen 7 5800X; 64 GB; RTX 2080Ti) and Linux (Ubuntu 20.04.2 LTS) PC (Intel i7-7800X; 94 GB; GTX Titan X). Figure Contributions: Tony Fong wrote and tested the software. This extended data file supports Figure 1. Download Extended Data 2, ZIP file. [file enu-eN-MNT-0127-22-s17.zip › ED2 PMT Offline Data Analysis Module/PRT/android/app/src/main/res/mipmap-mdpi/ic_launcher_round.png]

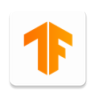

Supplement: Extended Data 2. — PMT offline data analysis module. Complete software package for offline data analysis. The package was installed and ran in an Anaconda environment (https://www.anaconda.com). Code was tested on a Windows 10 PC (AMD Ryzen 7 5800X; 64 GB; RTX 2080Ti) and Linux (Ubuntu 20.04.2 LTS) PC (Intel i7-7800X; 94 GB; GTX Titan X). Figure Contributions: Tony Fong wrote and tested the software. This extended data file supports Figure 1. Download Extended Data 2, ZIP file. [file enu-eN-MNT-0127-22-s17.zip › ED2 PMT Offline Data Analysis Module/PRT/android/app/src/main/res/mipmap-xhdpi/ic_launcher.png]

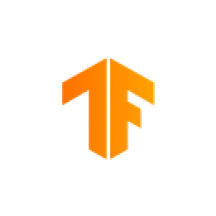

Supplement: Extended Data 2. — PMT offline data analysis module. Complete software package for offline data analysis. The package was installed and ran in an Anaconda environment (https://www.anaconda.com). Code was tested on a Windows 10 PC (AMD Ryzen 7 5800X; 64 GB; RTX 2080Ti) and Linux (Ubuntu 20.04.2 LTS) PC (Intel i7-7800X; 94 GB; GTX Titan X). Figure Contributions: Tony Fong wrote and tested the software. This extended data file supports Figure 1. Download Extended Data 2, ZIP file. [file enu-eN-MNT-0127-22-s17.zip › ED2 PMT Offline Data Analysis Module/PRT/android/app/src/main/res/mipmap-xhdpi/ic_launcher_foreground.png]

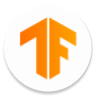

Supplement: Extended Data 2. — PMT offline data analysis module. Complete software package for offline data analysis. The package was installed and ran in an Anaconda environment (https://www.anaconda.com). Code was tested on a Windows 10 PC (AMD Ryzen 7 5800X; 64 GB; RTX 2080Ti) and Linux (Ubuntu 20.04.2 LTS) PC (Intel i7-7800X; 94 GB; GTX Titan X). Figure Contributions: Tony Fong wrote and tested the software. This extended data file supports Figure 1. Download Extended Data 2, ZIP file. [file enu-eN-MNT-0127-22-s17.zip › ED2 PMT Offline Data Analysis Module/PRT/android/app/src/main/res/mipmap-xhdpi/ic_launcher_round.png]

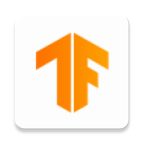

Supplement: Extended Data 2. — PMT offline data analysis module. Complete software package for offline data analysis. The package was installed and ran in an Anaconda environment (https://www.anaconda.com). Code was tested on a Windows 10 PC (AMD Ryzen 7 5800X; 64 GB; RTX 2080Ti) and Linux (Ubuntu 20.04.2 LTS) PC (Intel i7-7800X; 94 GB; GTX Titan X). Figure Contributions: Tony Fong wrote and tested the software. This extended data file supports Figure 1. Download Extended Data 2, ZIP file. [file enu-eN-MNT-0127-22-s17.zip › ED2 PMT Offline Data Analysis Module/PRT/android/app/src/main/res/mipmap-xxhdpi/ic_launcher.png]

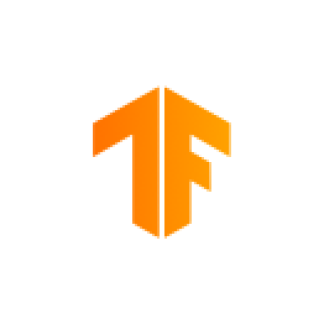

Supplement: Extended Data 2. — PMT offline data analysis module. Complete software package for offline data analysis. The package was installed and ran in an Anaconda environment (https://www.anaconda.com). Code was tested on a Windows 10 PC (AMD Ryzen 7 5800X; 64 GB; RTX 2080Ti) and Linux (Ubuntu 20.04.2 LTS) PC (Intel i7-7800X; 94 GB; GTX Titan X). Figure Contributions: Tony Fong wrote and tested the software. This extended data file supports Figure 1. Download Extended Data 2, ZIP file. [file enu-eN-MNT-0127-22-s17.zip › ED2 PMT Offline Data Analysis Module/PRT/android/app/src/main/res/mipmap-xxhdpi/ic_launcher_foreground.png]

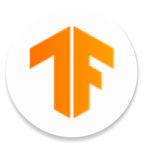

Supplement: Extended Data 2. — PMT offline data analysis module. Complete software package for offline data analysis. The package was installed and ran in an Anaconda environment (https://www.anaconda.com). Code was tested on a Windows 10 PC (AMD Ryzen 7 5800X; 64 GB; RTX 2080Ti) and Linux (Ubuntu 20.04.2 LTS) PC (Intel i7-7800X; 94 GB; GTX Titan X). Figure Contributions: Tony Fong wrote and tested the software. This extended data file supports Figure 1. Download Extended Data 2, ZIP file. [file enu-eN-MNT-0127-22-s17.zip › ED2 PMT Offline Data Analysis Module/PRT/android/app/src/main/res/mipmap-xxhdpi/ic_launcher_round.png]

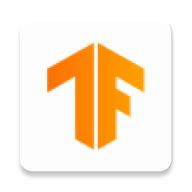

Supplement: Extended Data 2. — PMT offline data analysis module. Complete software package for offline data analysis. The package was installed and ran in an Anaconda environment (https://www.anaconda.com). Code was tested on a Windows 10 PC (AMD Ryzen 7 5800X; 64 GB; RTX 2080Ti) and Linux (Ubuntu 20.04.2 LTS) PC (Intel i7-7800X; 94 GB; GTX Titan X). Figure Contributions: Tony Fong wrote and tested the software. This extended data file supports Figure 1. Download Extended Data 2, ZIP file. [file enu-eN-MNT-0127-22-s17.zip › ED2 PMT Offline Data Analysis Module/PRT/android/app/src/main/res/mipmap-xxxhdpi/ic_launcher.png]

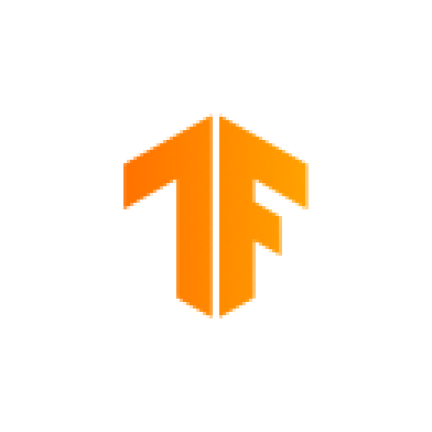

Supplement: Extended Data 2. — PMT offline data analysis module. Complete software package for offline data analysis. The package was installed and ran in an Anaconda environment (https://www.anaconda.com). Code was tested on a Windows 10 PC (AMD Ryzen 7 5800X; 64 GB; RTX 2080Ti) and Linux (Ubuntu 20.04.2 LTS) PC (Intel i7-7800X; 94 GB; GTX Titan X). Figure Contributions: Tony Fong wrote and tested the software. This extended data file supports Figure 1. Download Extended Data 2, ZIP file. [file enu-eN-MNT-0127-22-s17.zip › ED2 PMT Offline Data Analysis Module/PRT/android/app/src/main/res/mipmap-xxxhdpi/ic_launcher_foreground.png]

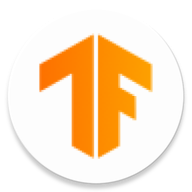

Supplement: Extended Data 2. — PMT offline data analysis module. Complete software package for offline data analysis. The package was installed and ran in an Anaconda environment (https://www.anaconda.com). Code was tested on a Windows 10 PC (AMD Ryzen 7 5800X; 64 GB; RTX 2080Ti) and Linux (Ubuntu 20.04.2 LTS) PC (Intel i7-7800X; 94 GB; GTX Titan X). Figure Contributions: Tony Fong wrote and tested the software. This extended data file supports Figure 1. Download Extended Data 2, ZIP file. [file enu-eN-MNT-0127-22-s17.zip › ED2 PMT Offline Data Analysis Module/PRT/android/app/src/main/res/mipmap-xxxhdpi/ic_launcher_round.png]

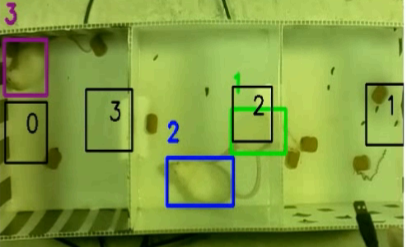

Supplement: Extended Data 2. — PMT offline data analysis module. Complete software package for offline data analysis. The package was installed and ran in an Anaconda environment (https://www.anaconda.com). Code was tested on a Windows 10 PC (AMD Ryzen 7 5800X; 64 GB; RTX 2080Ti) and Linux (Ubuntu 20.04.2 LTS) PC (Intel i7-7800X; 94 GB; GTX Titan X). Figure Contributions: Tony Fong wrote and tested the software. This extended data file supports Figure 1. Download Extended Data 2, ZIP file. [file enu-eN-MNT-0127-22-s17.zip › ED2 PMT Offline Data Analysis Module/PRT/imgs/3chamber.PNG]

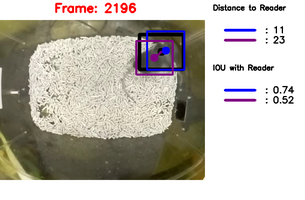

Supplement: Extended Data 2. — PMT offline data analysis module. Complete software package for offline data analysis. The package was installed and ran in an Anaconda environment (https://www.anaconda.com). Code was tested on a Windows 10 PC (AMD Ryzen 7 5800X; 64 GB; RTX 2080Ti) and Linux (Ubuntu 20.04.2 LTS) PC (Intel i7-7800X; 94 GB; GTX Titan X). Figure Contributions: Tony Fong wrote and tested the software. This extended data file supports Figure 1. Download Extended Data 2, ZIP file. [file enu-eN-MNT-0127-22-s17.zip › ED2 PMT Offline Data Analysis Module/PRT/imgs/failed_match_example.png]

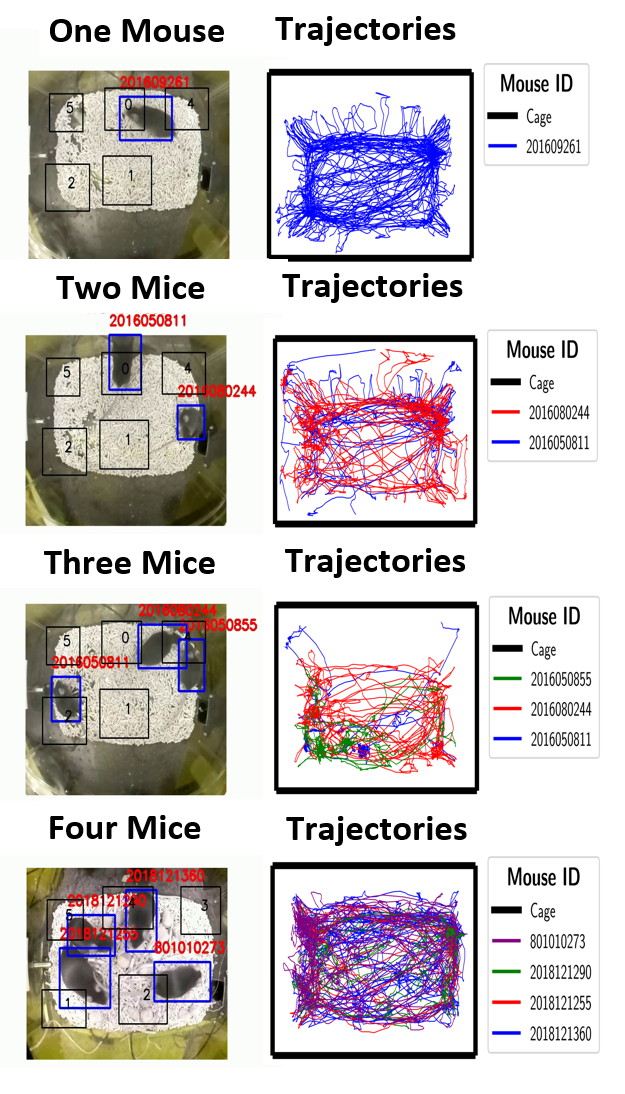

Supplement: Extended Data 2. — PMT offline data analysis module. Complete software package for offline data analysis. The package was installed and ran in an Anaconda environment (https://www.anaconda.com). Code was tested on a Windows 10 PC (AMD Ryzen 7 5800X; 64 GB; RTX 2080Ti) and Linux (Ubuntu 20.04.2 LTS) PC (Intel i7-7800X; 94 GB; GTX Titan X). Figure Contributions: Tony Fong wrote and tested the software. This extended data file supports Figure 1. Download Extended Data 2, ZIP file. [file enu-eN-MNT-0127-22-s17.zip › ED2 PMT Offline Data Analysis Module/PRT/imgs/Home_cage_tracks.PNG]

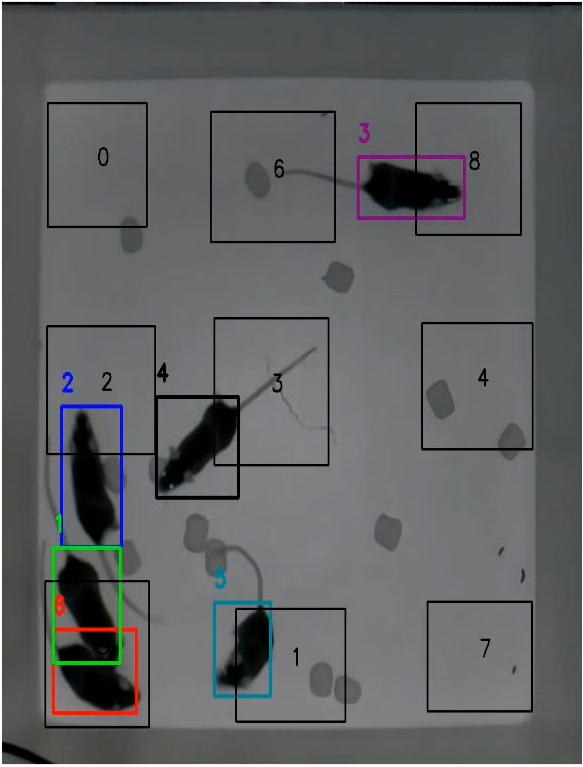

Supplement: Extended Data 2. — PMT offline data analysis module. Complete software package for offline data analysis. The package was installed and ran in an Anaconda environment (https://www.anaconda.com). Code was tested on a Windows 10 PC (AMD Ryzen 7 5800X; 64 GB; RTX 2080Ti) and Linux (Ubuntu 20.04.2 LTS) PC (Intel i7-7800X; 94 GB; GTX Titan X). Figure Contributions: Tony Fong wrote and tested the software. This extended data file supports Figure 1. Download Extended Data 2, ZIP file. [file enu-eN-MNT-0127-22-s17.zip › ED2 PMT Offline Data Analysis Module/PRT/imgs/open_field.PNG]

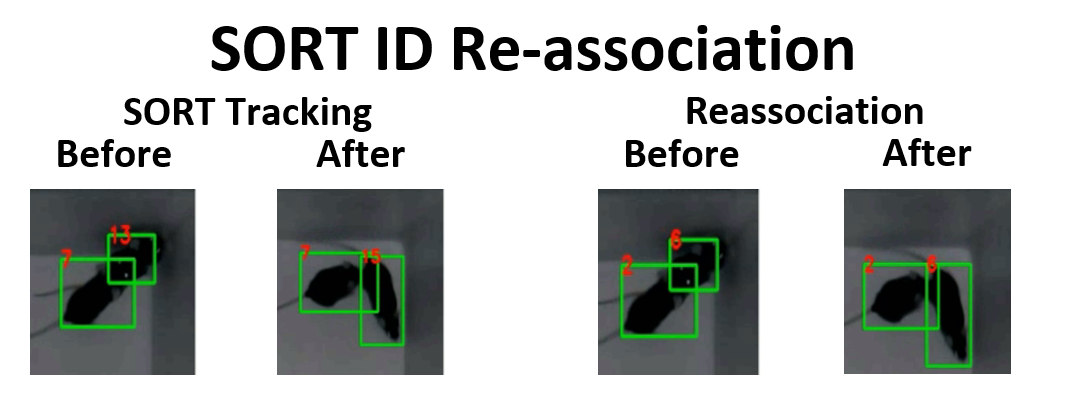

Supplement: Extended Data 2. — PMT offline data analysis module. Complete software package for offline data analysis. The package was installed and ran in an Anaconda environment (https://www.anaconda.com). Code was tested on a Windows 10 PC (AMD Ryzen 7 5800X; 64 GB; RTX 2080Ti) and Linux (Ubuntu 20.04.2 LTS) PC (Intel i7-7800X; 94 GB; GTX Titan X). Figure Contributions: Tony Fong wrote and tested the software. This extended data file supports Figure 1. Download Extended Data 2, ZIP file. [file enu-eN-MNT-0127-22-s17.zip › ED2 PMT Offline Data Analysis Module/PRT/imgs/SORT_re.PNG]

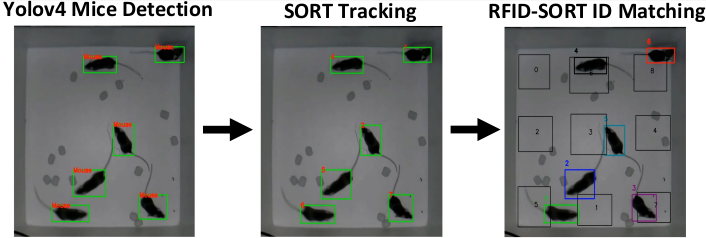

Supplement: Extended Data 2. — PMT offline data analysis module. Complete software package for offline data analysis. The package was installed and ran in an Anaconda environment (https://www.anaconda.com). Code was tested on a Windows 10 PC (AMD Ryzen 7 5800X; 64 GB; RTX 2080Ti) and Linux (Ubuntu 20.04.2 LTS) PC (Intel i7-7800X; 94 GB; GTX Titan X). Figure Contributions: Tony Fong wrote and tested the software. This extended data file supports Figure 1. Download Extended Data 2, ZIP file. [file enu-eN-MNT-0127-22-s17.zip › ED2 PMT Offline Data Analysis Module/PRT/imgs/Track_flow.png]

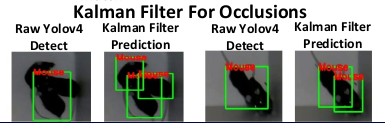

Supplement: Extended Data 2. — PMT offline data analysis module. Complete software package for offline data analysis. The package was installed and ran in an Anaconda environment (https://www.anaconda.com). Code was tested on a Windows 10 PC (AMD Ryzen 7 5800X; 64 GB; RTX 2080Ti) and Linux (Ubuntu 20.04.2 LTS) PC (Intel i7-7800X; 94 GB; GTX Titan X). Figure Contributions: Tony Fong wrote and tested the software. This extended data file supports Figure 1. Download Extended Data 2, ZIP file. [file enu-eN-MNT-0127-22-s17.zip › ED2 PMT Offline Data Analysis Module/PRT/kalmen_predic.png]

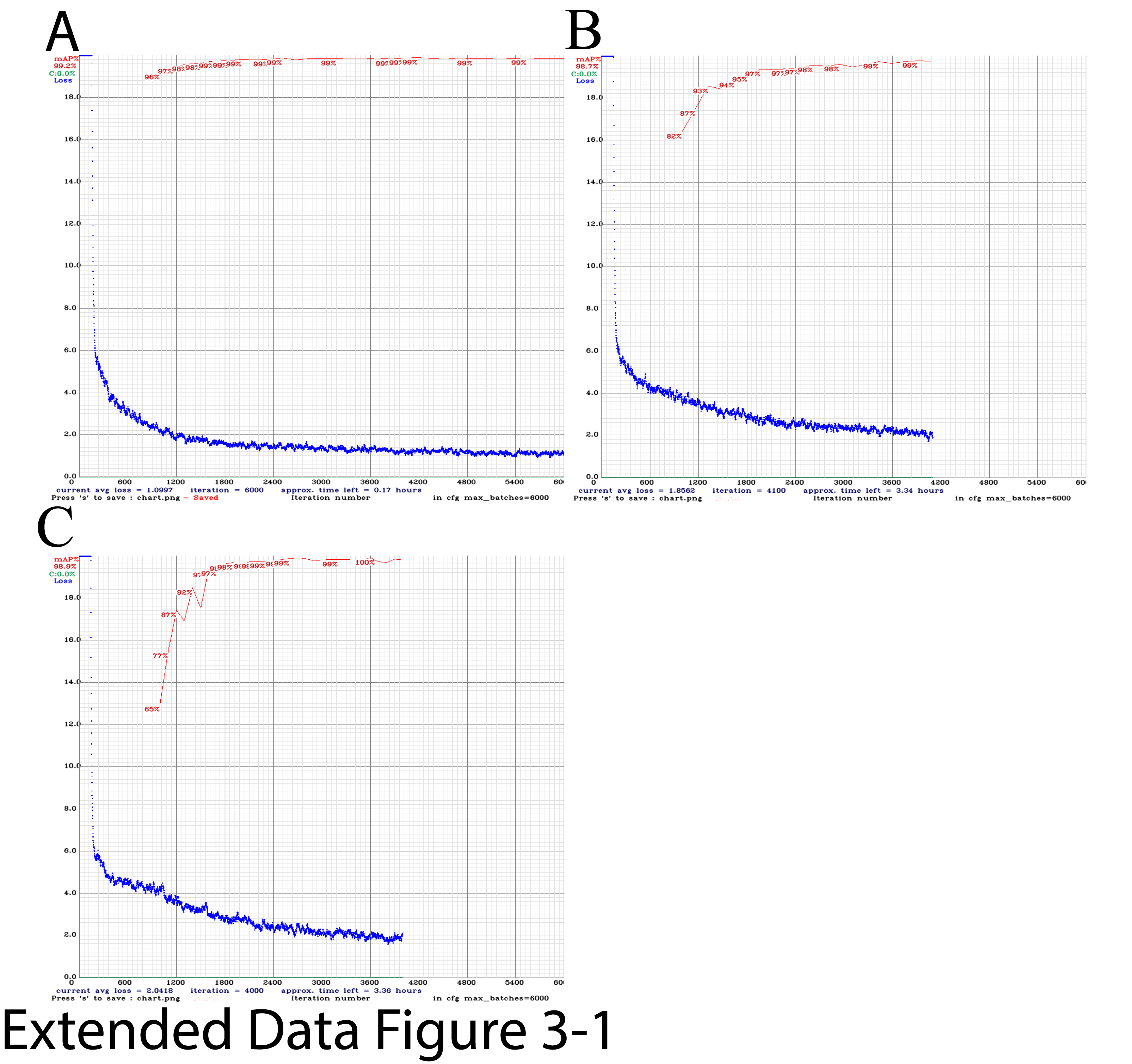

Supplement: Extended Data Figure 3-1 — Yolov4 Training Loss and mAP. A, Training loss and mAP on home-cage weights using 2500 images with 6000 iterations. B, Training loss and mAP on the open-field arena using 300 images with 6000 iterations. The training was manually stopped when mAP > 99.5%. C, Training loss and mAP on the sociability chamber arena using 300 images with 6000 iterations. The training was manually stopped when mAP > 99.5. Figure Contributions: Tony Fong labeled the images and trained the weights. This extended data figure supports Figure 3. Download Figure 3-1, TIF file. [file enu-eN-MNT-0127-22-s13.tif]

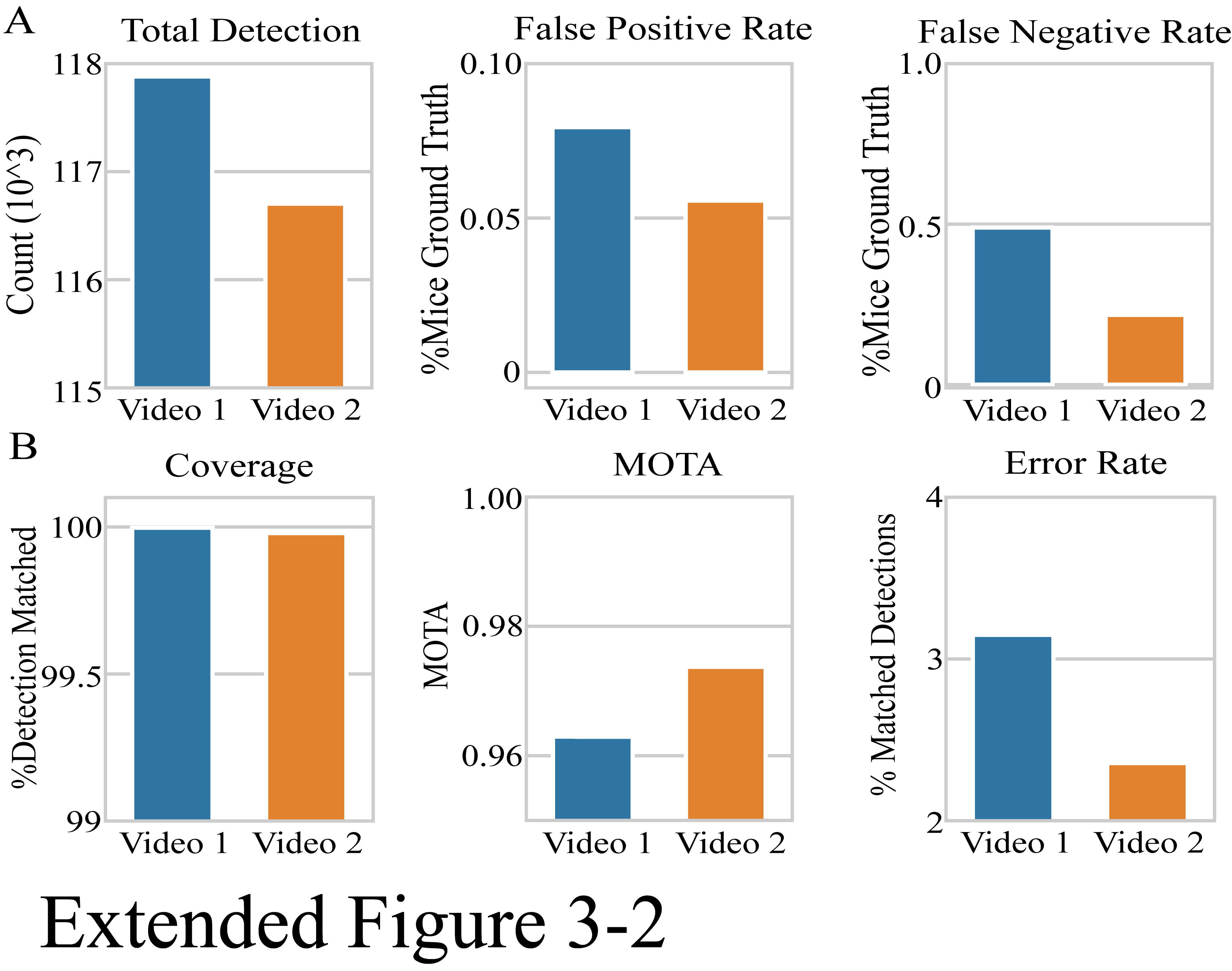

Supplement: Extended Data Figure 3-2 — Evaluation of PMT performance on the open-field arena with six mice. A, PMT detection Performance was measured by total detection, the appearance of false negative detections, and false positive detections. False positive and false negative detections were expressed as a % of total ground truth mice in videos. B, Tracking performance of PMT was measured by coverage, MOTA, and % errors in detections with RFID matched. Coverage represents % of detections being matched with an RFID tag. A total of two videos were evaluated. Figure Contributions: Tony Fong and Hao Hu analyzed the data. Tony Fong created the figure. This extended data figure supports Figure 3. Download Figure 3-2, TIF file. [file enu-eN-MNT-0127-22-s14.tif]

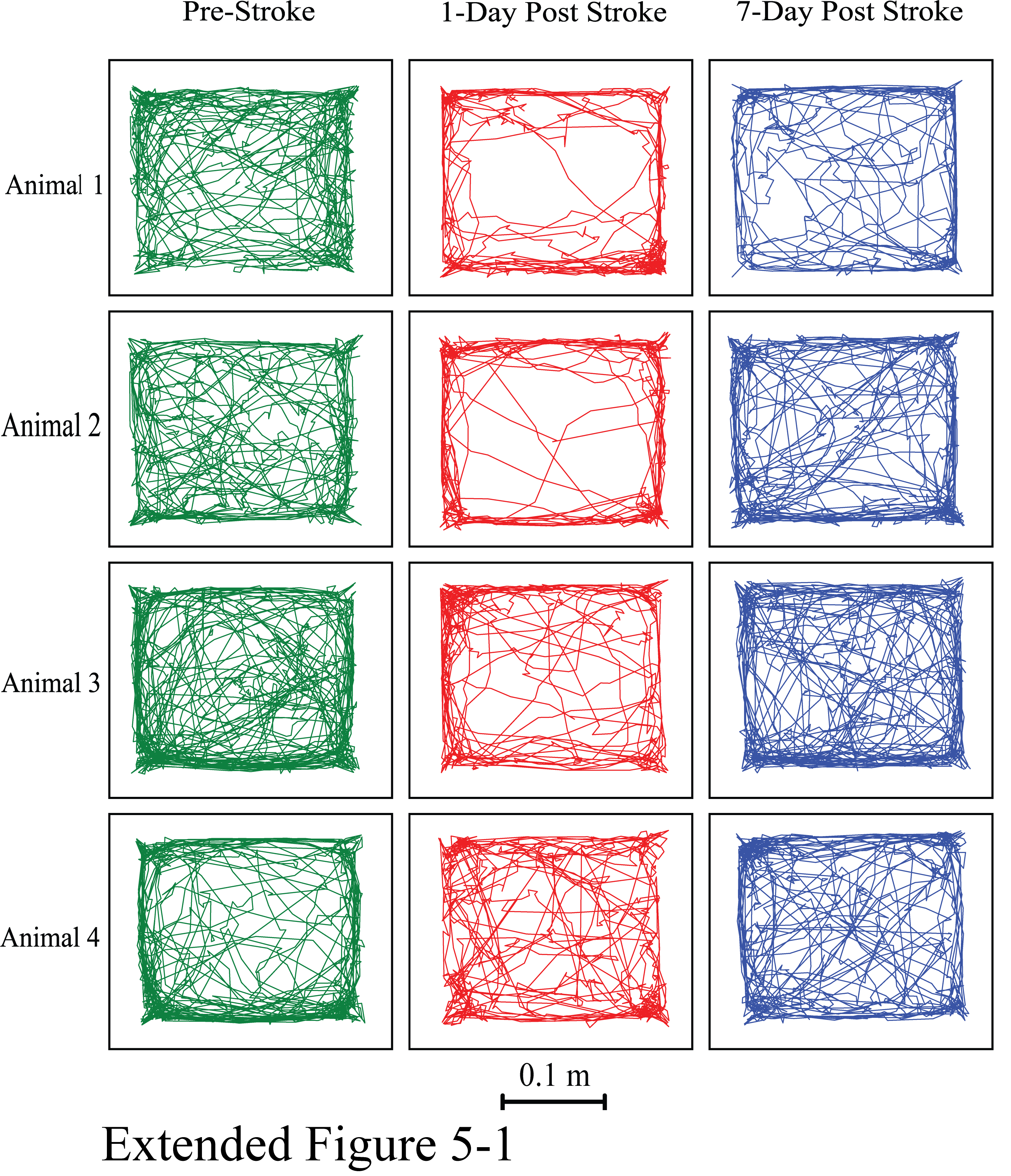

Supplement: Extended Data Figure 5-1 — Open-field travel trajectories of mice prestroke, post 1 and 7 d after stroke. Ten-minute open-field recording 24 h before stroke (green), 1 d poststroke (red), and 7 d poststroke (blue). Trajectories were smoother using Ramer–Douglas–Peucker algorithm (ε = 10). Figure Contributions: Tony Fong and Pankaj Gupta analyzed the data. Tony Fong created the figure. This extended data figure supports Figure 5. Download Figure 5-1, TIF file. [file enu-eN-MNT-0127-22-s15.tif]
