# Supplementary material for: PyMouseTracks: Flexible Computer Vision and RFID-Based System for Multiple Mouse Tracking and Behavioral Assessment
Source: eNeuro. 2023 May 12;10(5):ENEURO.0127-22.2023. doi: 10.1523/ENEURO.0127-22.2023 (PMC10198609; doi:10.1523/ENEURO.0127-22.2023)
Supplement: Extended Data 3. — Assembly and installation instructions. A complete guide on setting up the hardware components and installing the software for PMT. Links to video tutorials and demonstrations can also be found in the guide. Download Extended Data 3, DOCX file [file enu-eN-MNT-0127-22-s18.docx]

**PyMouseTracks Supplementary Material**

**User Guide**

**PiMouseTracks Build Guide**

**1. Online Raspberry Pi Data Collection**

**1.1 Parts Required**
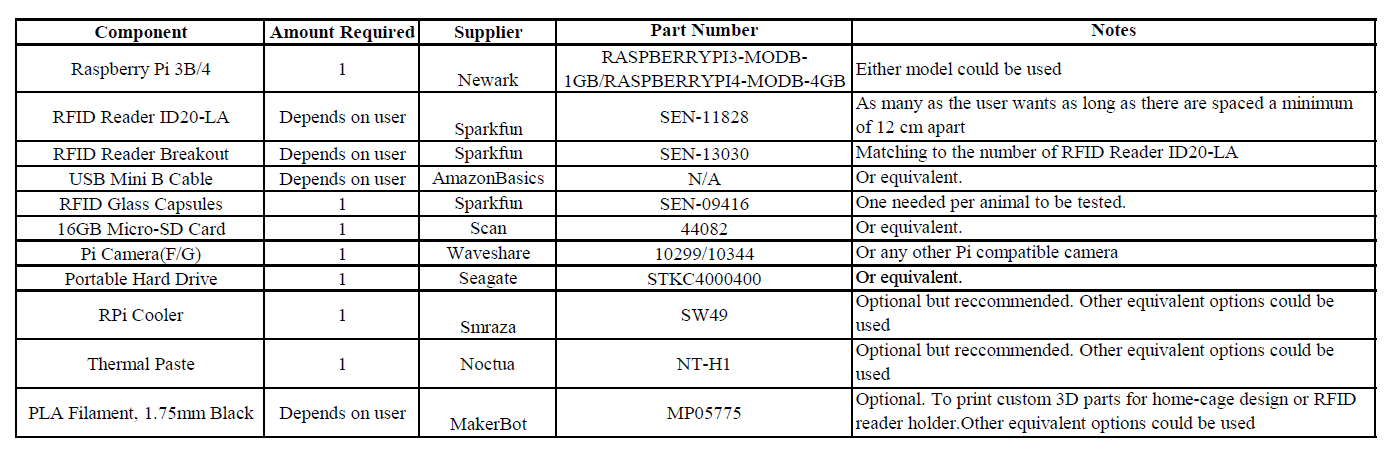


- All components required for the online raspberry pi data collection are listed in the table above and can also be found in our publication. All optional STL files for 3D printing are also provided on the PyMouseTracks OSF and Hackaday page.
- Any rodent arena can be used, but it is advised to use arenas formed of a thin material not exceeding 5 mm in thickness to obtain sensitive RFID readings throughout the recording sessions
- Any raspberry pi camera with a CSI camera port will work.
- Any pi camera compatible with the python module Picamera v1.10 can be used as long as the camera can capture an overhead view of the arena. Currently, supported camera sensors include the OV5647 or V1 camera, the IMX219 or V2 camera and the IMX477 or HQ camera.[^1^](https://www.zotero.org/google-docs/?mP4l8q)
- As the RFID readers are powered by the USB port connection, it is advised to use a USB hub with its own stand-alone power supply
- Any adhesive agents such as glue or tape can be used to fix the readers to the arena and the camera position

**1.2 Hardware Installation**
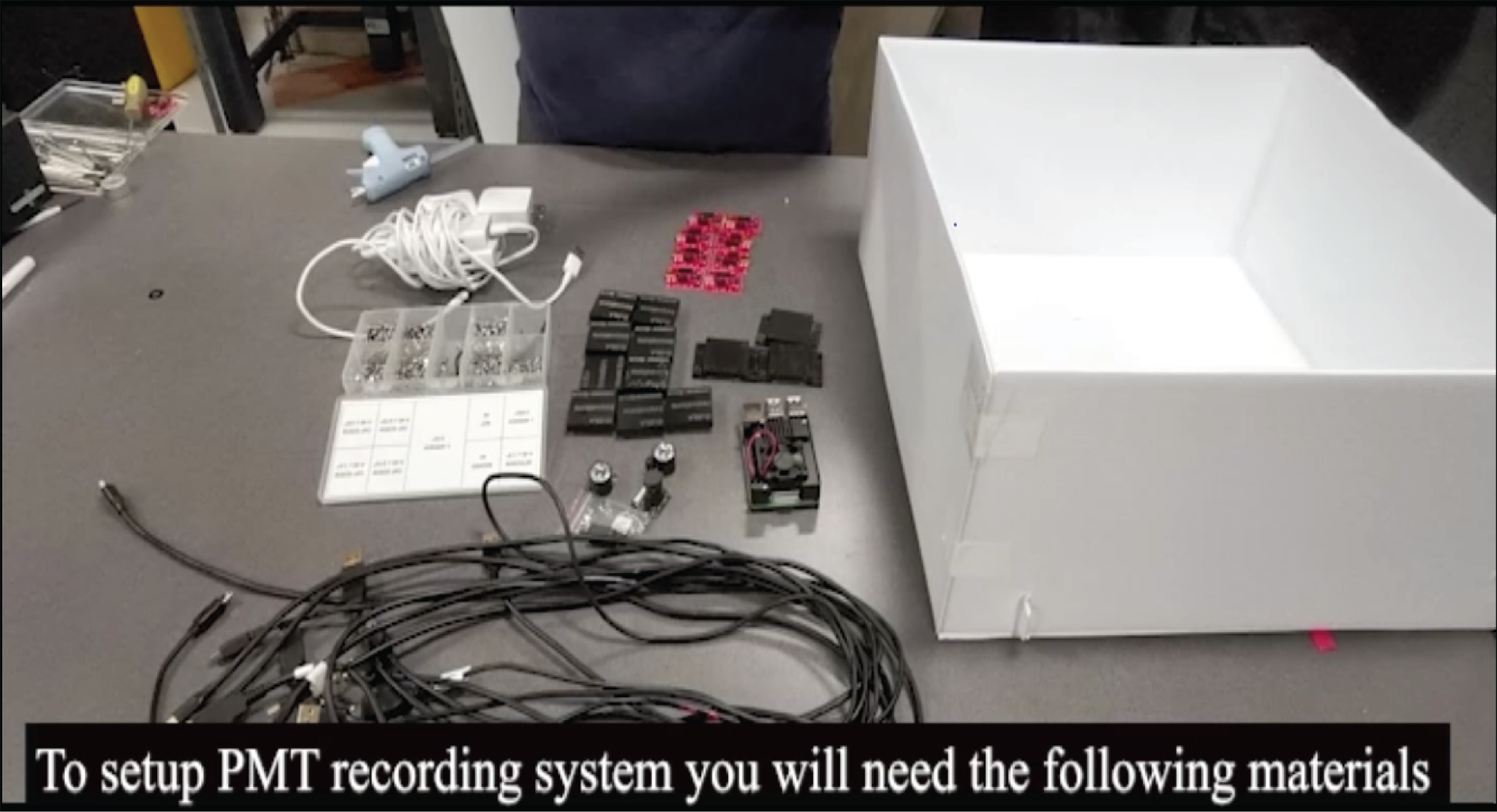


Video link: <https://youtu.be/zepiC6SK2Fk>

A tutorial video on setting up the online data collection system can be found in the above link. Overall, the installation process follows a simple plug-and-play principle with minimal hardware knowledge or altercations needed.

1. Desolder the buzz pin on all Sparkfun RFID reader bases.


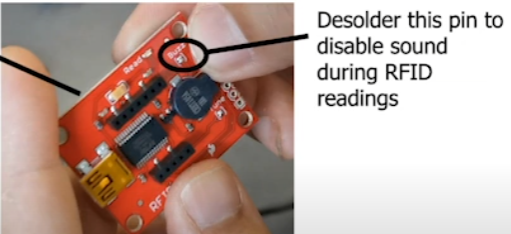


1. (Optional) Attach the reader base to the RFID reader holder modules with M2/M2.5 bolts.
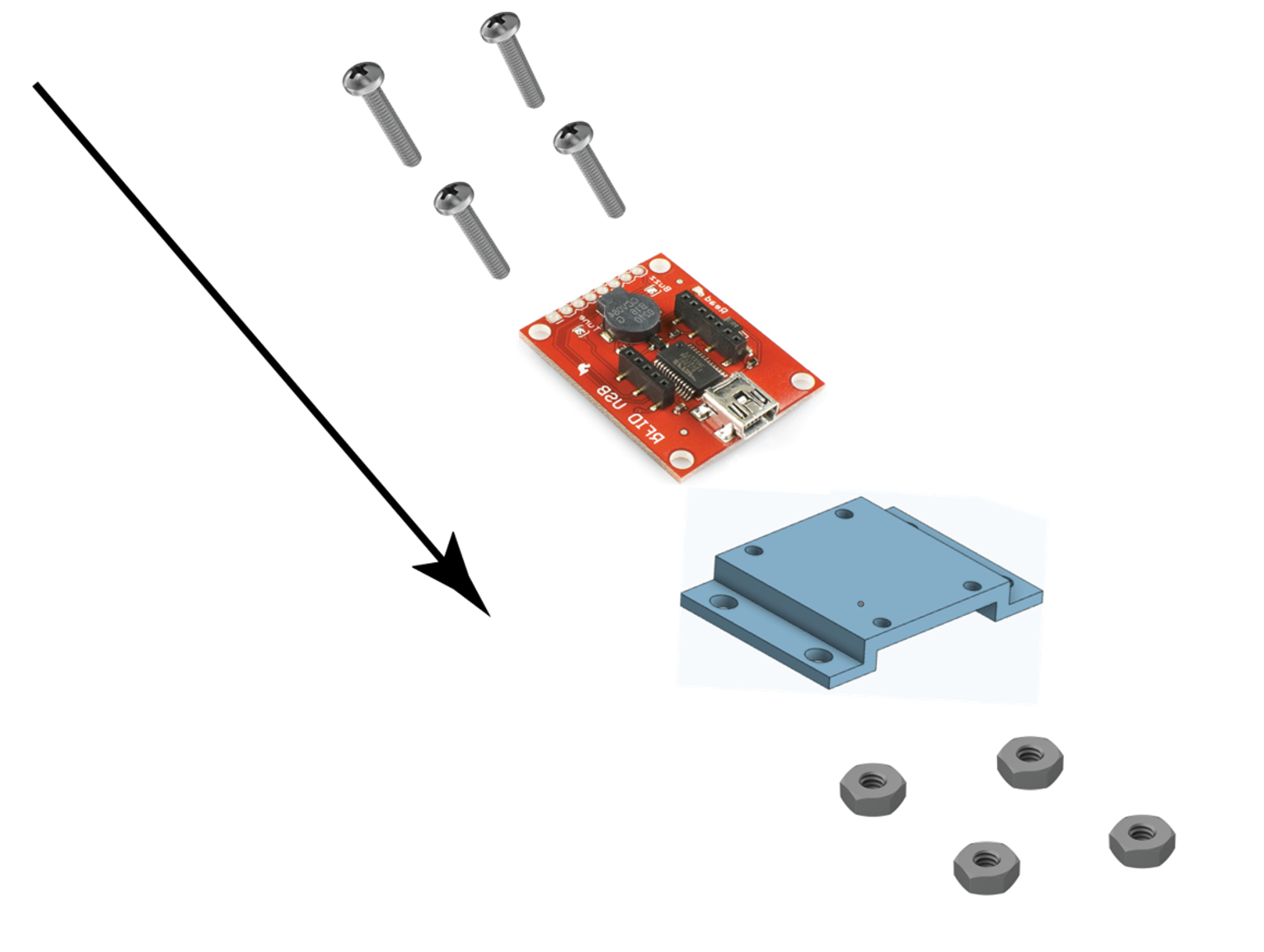

2. Attach the RFID reader ID20-LA modules to the RFID reader base. (Assemble as many as you need)
3. Place the RFID reader assembly under the arena of choice at desirable locations with adhesive. Ensure that each reader is at least from each other 12 cm apart to minimize RFID interference. Most importantly, ensure to mark down/take note of the placements from images generated from the overhead camera.
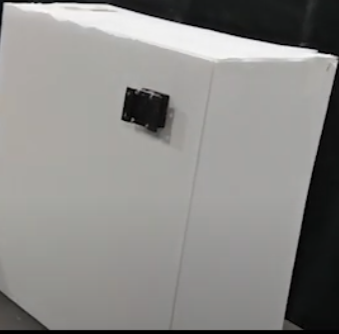

4. Connect the RFID reader bases to the USB hub(s) with the mini-USB cables. Then connect the USB hub(s) to the raspberry pi.


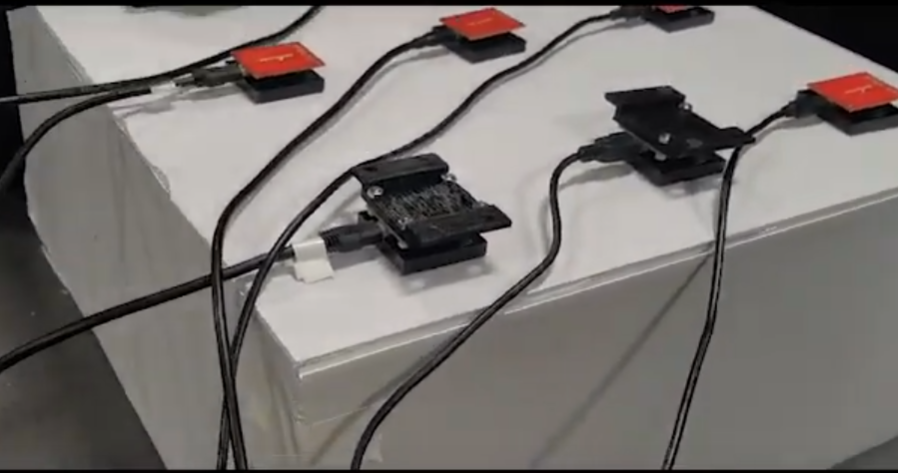

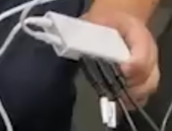


1. Connect the portable hard drive to the Raspberry Pi
2. Connect the picamera to the Raspberry pi through the CSI ribbon cable.
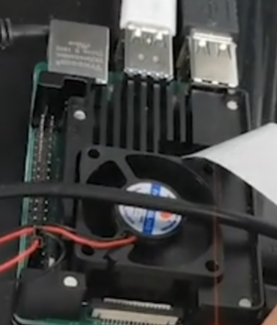

3. (Optional) Attach IR illumination to the picamera
4. Use adhesive to place the picamera at a position with an overhead view of the arena.
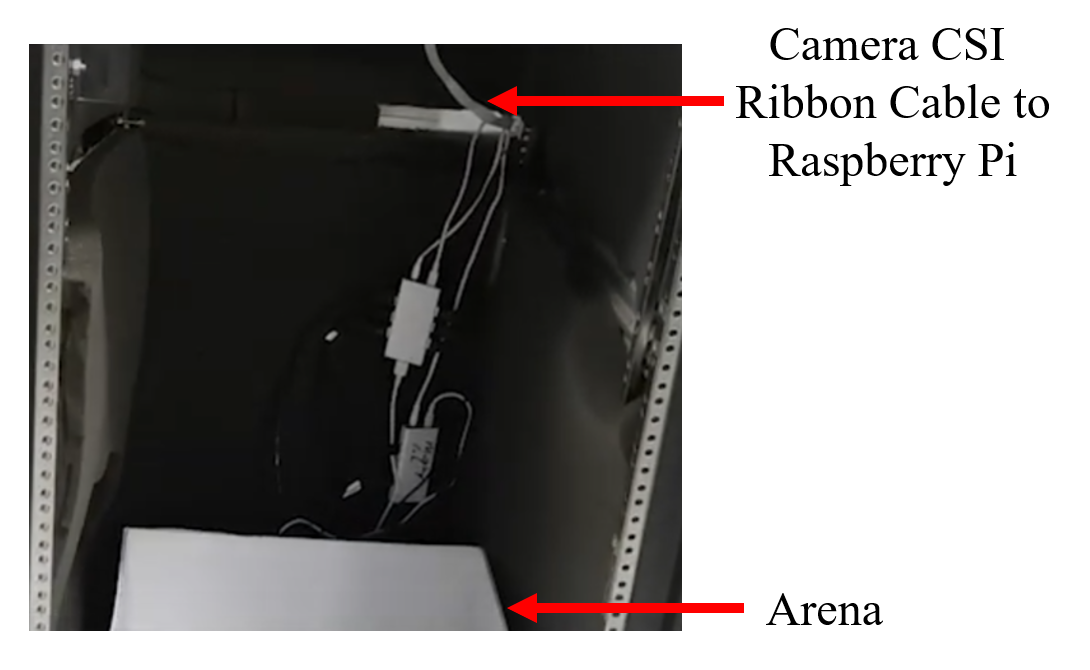


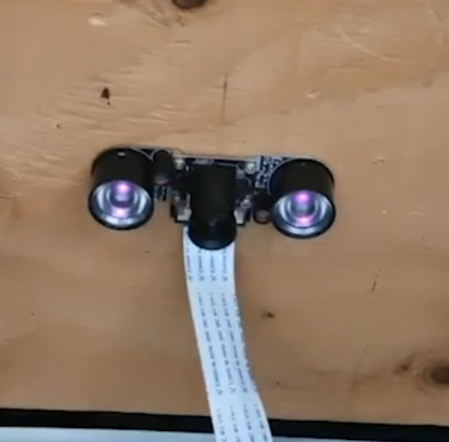


**1.3 Software Installation**

**
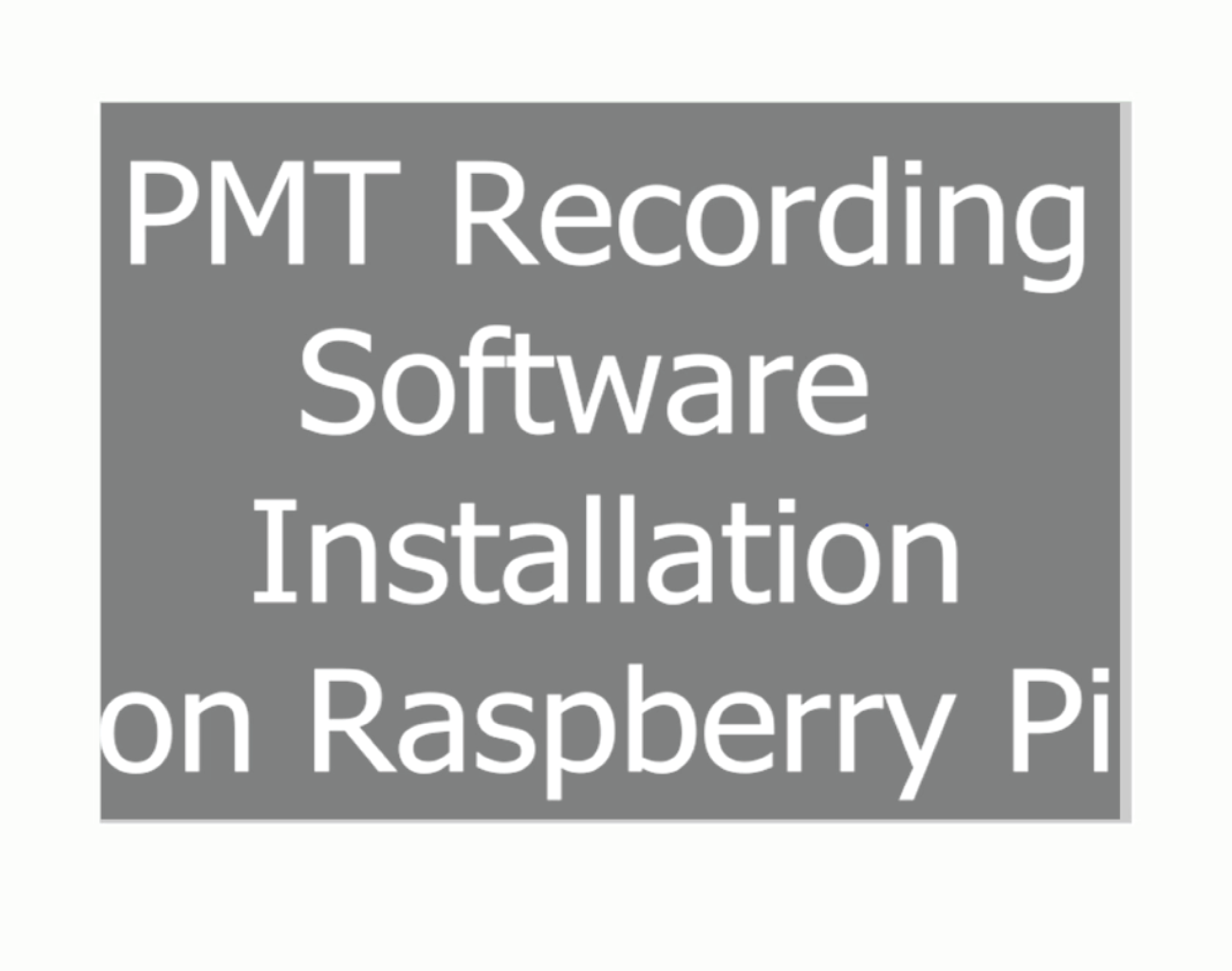
**

A tutorial video on configuring the Raspberry Pi and installing the online data acquisition software can be found in the above link. The software has been tested out on Debian 9 (“stretch”) and Debian 10 (‘buster’).

**Video link:** <https://youtu.be/aL_f5Q7G6nw>

**Important: PMT online recording only works on buster/legacy or legacy camera enabled Bullseye OS. It does not work with libcamera or picamera2.**

1. Flash Debian 9 (“stretch”) and Debian 10 (‘buster’) on the SD card. For more detailed information on setting up a Raspberry Pi, please refer to the official documentation on the Raspberry Pi Foundation webpage.[^2^](https://www.zotero.org/google-docs/?Mqqh32)
2. Insert the SD card into the Raspberry Pi and power up the system. Ensure the Raspberry Pi has an internet connection via wifi or the ethernet port.
3. Once the system is booted up, clone the PiMouseTracks files into the desired location on to the Raspberry Pi. Files could be found on the PiMouseTracks Github page. Alternatively, open the terminal and enter the following command:

git clone https://github.com/tf4ong/tracker_rpi

For our example, we will clone the folder into the ‘/home/pi/’ directory.

1. In the terminal enter the PyMouseTracks directory, by entering the following command:

cd tracker_rpi

1. Initialize the setup script by entering the following command:

sudo chmod 777 setup.sh

Or

sudo chmod 711 setup.sh

1. To install the software, enter:

sudo ./setup.sh

1. After the installation, initialize the picamera by accessing the interface menu in Raspberry Pi configurations:
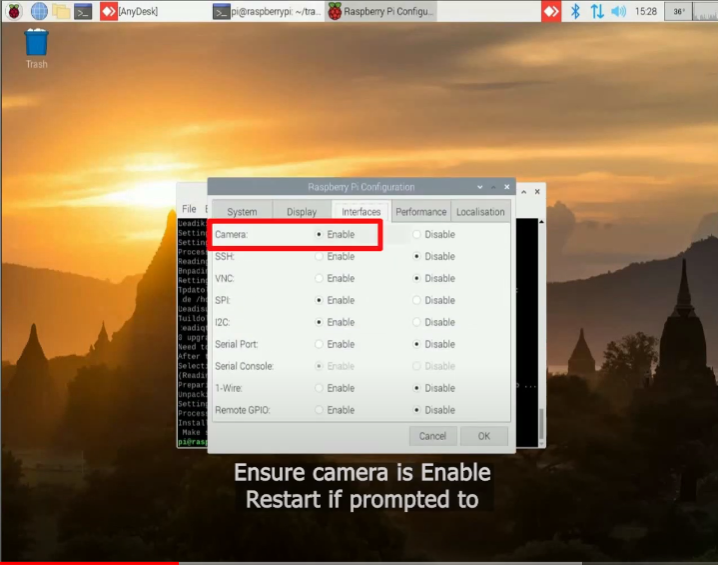

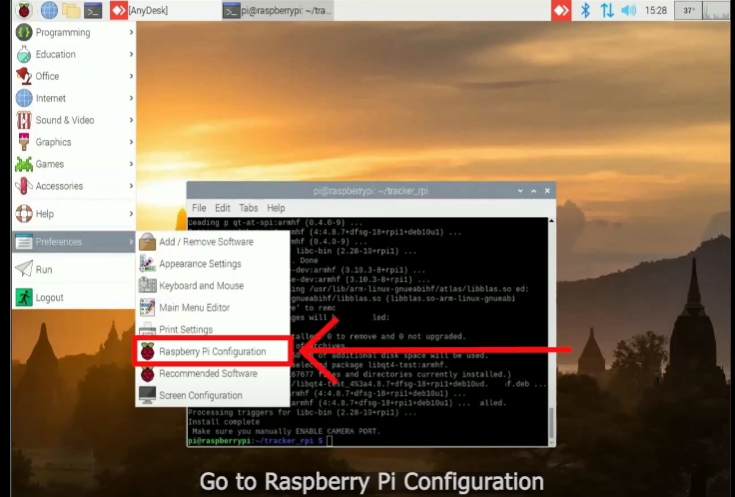


**1.4 Data Collection Settings**

All related settings can be adjusted by accessing the config.ini file in the PyMouseTracks directory. Using file explorer, navigate to the PyMouseTracks directory and edit the config.ini file using the text editor to achieve desired recording settings.


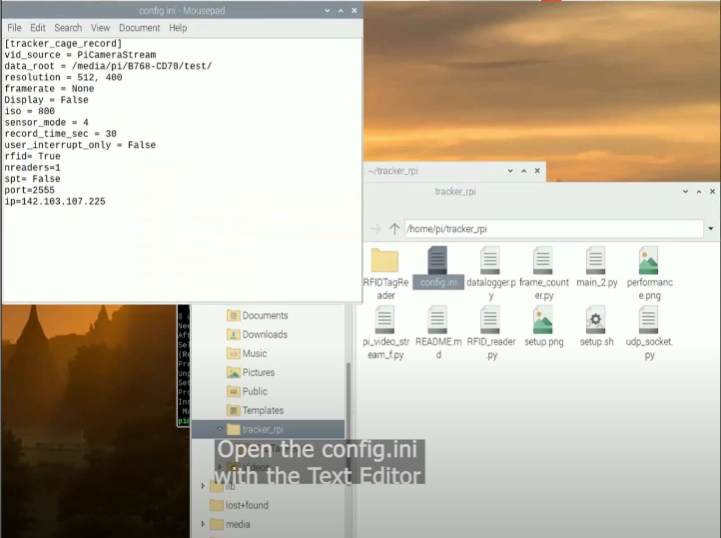


1. **data_root:** full path to the data output, where your data would be saved
2. **resolution:** resolution (WxH; separated by a comma) of the video recording
3. **framerate:** the framerate of the recording. Please refer to the official picamera documentation for the framerates supported by V1 and V2 cameras. (<https://picamera.readthedocs.io/en/release-1.13/fov.html>)
4. **iso:** signal and sensitivity gain of the camera, sets the exposure mode. Valid values include 100, 200, 320, 400, 500, 640, 800. Lower values indicate lower sensitivities to light. If recording only under IR illumination, we recommend setting iso to 800.
5. **sensor mode:** dictate the camera data feed into the onboard GPU. Hence, specific modes will determine the frame rate at a given resolution. Valid values 0-7. For further information, please refer to the official Raspberry Pi camera documentation[^3^](https://www.zotero.org/google-docs/?ZXcFeG). If set to 0, the most suitable mode for the resolution and framerate combination would be used.
6. **record_time_sec:** sets the length of recording in seconds. Only active if user_interrupt_only = False.
7. **user_interrupt_only**: valid values are True and False. When set to True, recording is stopped by ctrl + c. When set to False, recording duration is defined by the **record_time_sec** setting.
8. **rfid**: valid values are True and False. When set to True, RFID readers are used.
9. **nreaders:** number of RFID readers attached and used in the online data collection. There is no limit on the number of RFID readers that can be attached. In theory, the limit is up to the bandwidth of the 3 USB ports (very large compared to that what the RFID readers can generate) available on the Raspberry Pi as one will be used by the storage device. Currently, up to 9 readers (connected through 2 USB hubs) have been tested.

**1.5 Usage**

1. To begin a recording, open the terminal and navigate to the PyMouseTracks directory.
2. After setting the desired settings (Read section 1.3 for more details) enter the following command to begin a recording.

sudo python3 main_2.py

1. After the recording is done, approximate framerate and elapsed time will be displayed.
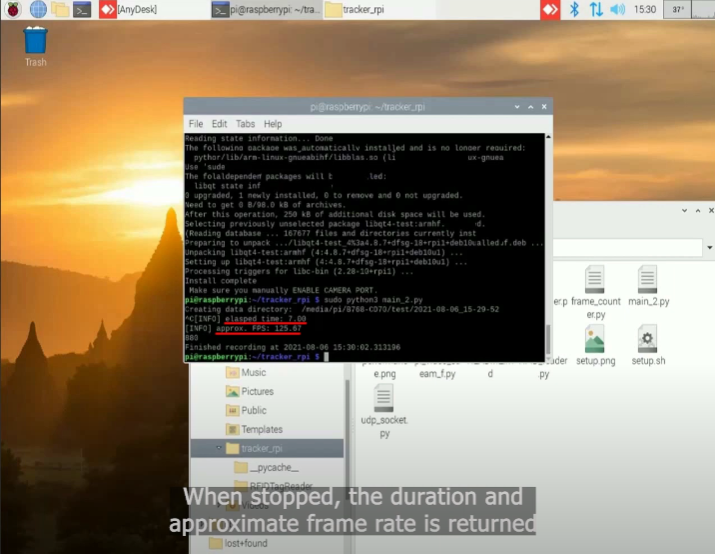

2. Data will be stored at the location path set in data_root in the config.ini.
3. The folder containing the recording will be named by the start timestamp of the recording. Files include the h264 video, frame timestamp, and RFID readings.


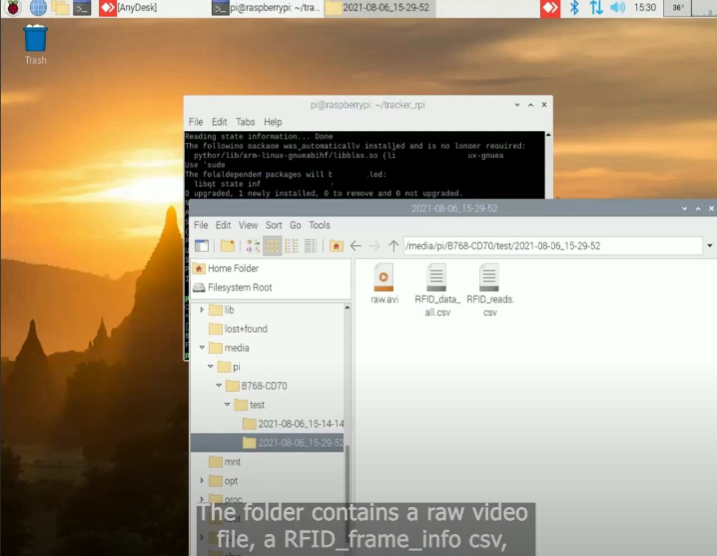


**1.6 Document Reader Location for Offline analysis**

For the offline analysis, RFID reader ID and their location on the output video image are needed. As long as the Raspberry Pi is not restarted and that no USB devices are removed/added, the RFID readers and their respective ID will not change.

1. Label the location of the RFID readers with tape or any removable markings. Alternatively, a clear arena can be used.
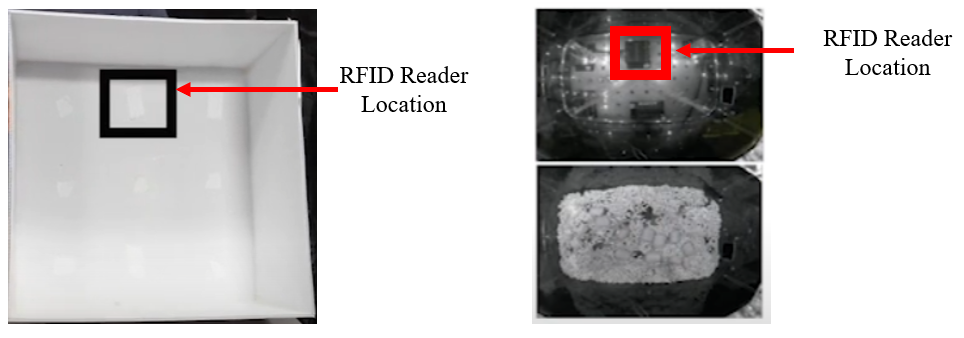

2. Start a recording session.
3. Hover an RFID tag at each reader location once. Ensure that the reader reads the tag by monitoring the terminal output. If a tag is read by a reader, it will be printed out.
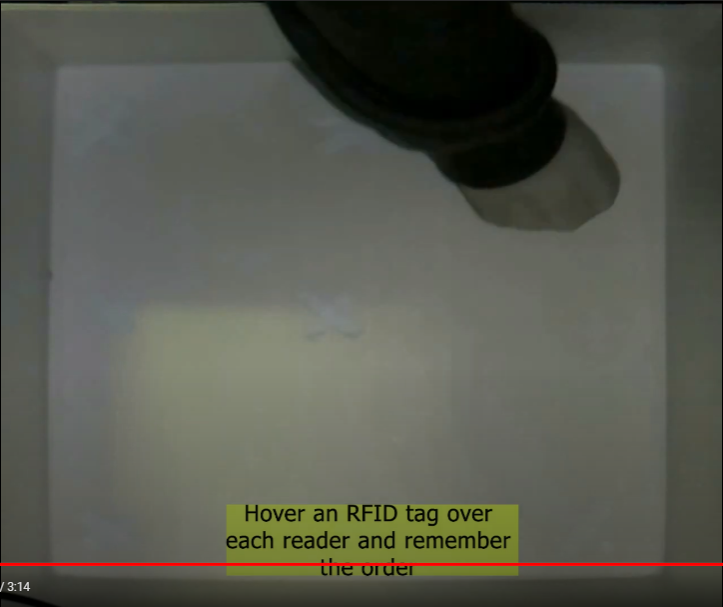

4. Stop the recording session. Data will be stored at the designated folder path defined in config.ini.
5. The respective ID of each RFID reader will be in the RFID_read.csv file:
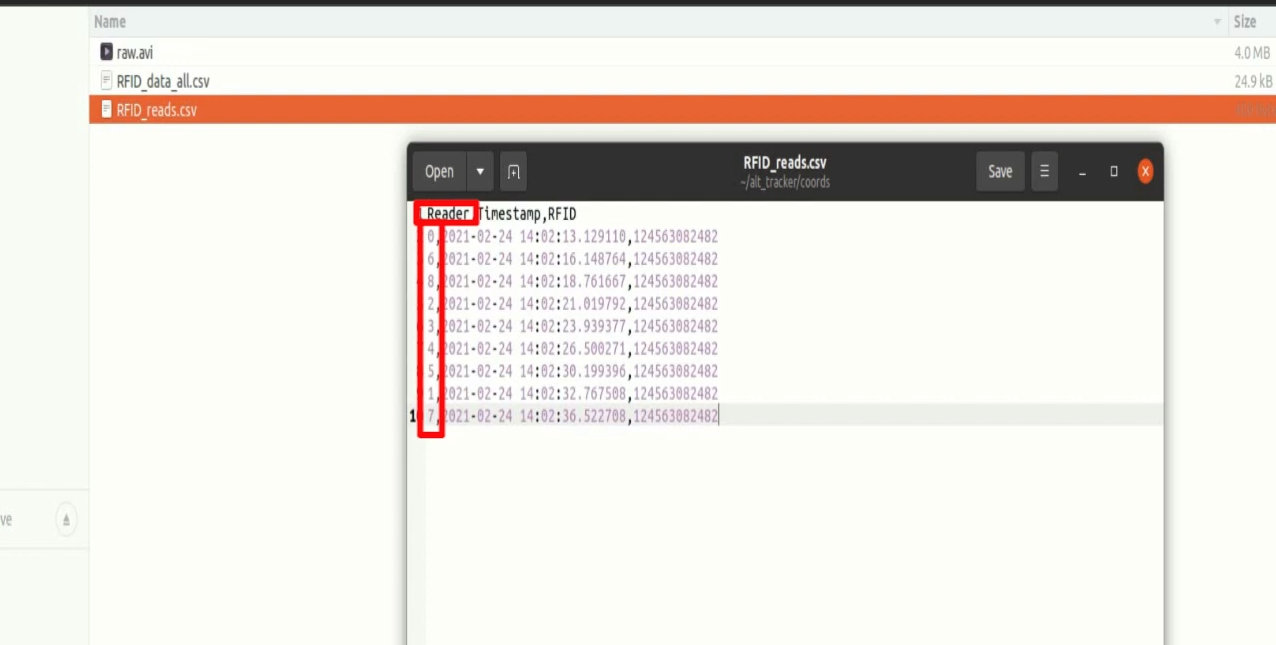


**2. Offline Data Analyst**

The offline data analysis can be performed on any CUDA-capable computer or in a google lab notebook (free GPU) which we provide with the code. The offline data analyst pipeline is installed and will run in Anaconda. The pipeline has been tested in OS Windows 10 and Ubuntu 20.04.

**2.1 Running the Analysis**

1. Download the offline analysis files. The files can be downloaded on the PyMouse(Rodent)Tracks Github page. Alternatively, files can be loaded with Git in the terminal(Linux)/anaconda prompt (windows). Clone the repository with the following command.

git clone <https://github.com/tf4ong/PMT>


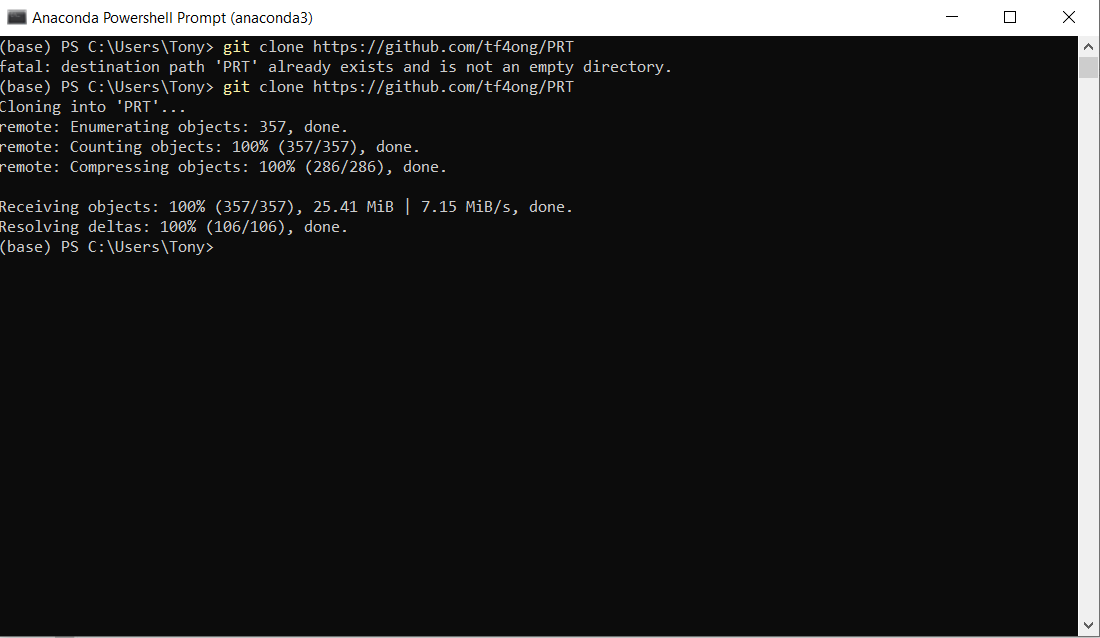


1. In terminal (Linux) or anaconda prompt (Windows), navigate to the offline analysis directory and enter the following command to create the environment.

Conda env create -f conda-env.yml

1. Install the dependencies

pip install -r requirements.txt

1. Download the required darknet weights list in our publication or on our GitHub. Alternatively, you can train your own weights using the original Darknet implementation of Yolov4. For further instructions, in the training section of this manual.
2. Convert the darknet weights to TensorFlow weights with the following command:

python save_model.py --weights <FULL PATH TO DARKNET WEIGHTS> --output <FULL PATH TO TENSORFLOW OUTPUT FOLDER> --input_size <INPUT RESOLUTION SIZE > --model yolov4

- Input size will be dependent on the training resolution of the original darknet weights
- Higher input size will increase accuracy but also inference time.
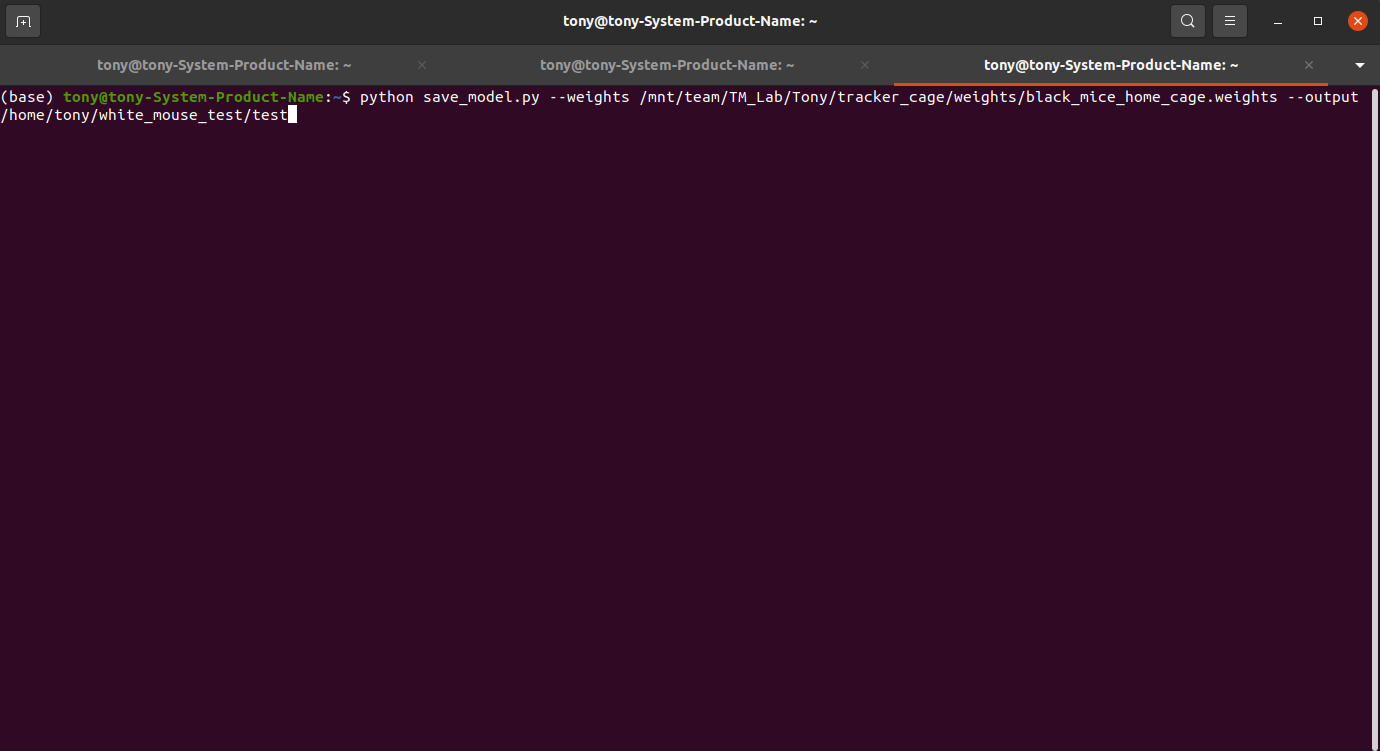


1. Configure the settings file for tracking. A template with default settings can be found in the configs folder. For further information please refer to section 2.1 on settings.
2. Acquire RFID label coordinates with the select_roi.py script

python select_roi.py --frame_count <REFENCE FRAME TO USE> --reader_count <NUMBER OF RFID READERS TO LABEL> --vid_path <PATH TO VIDEO WITH RFID READER LOCATION>

- Please refer to section 1.6 to generate RFID reader location video

1. Select ROIs of readers and enter their respective IDs in the terminal. Please refer to section 1.6 for reader IDs


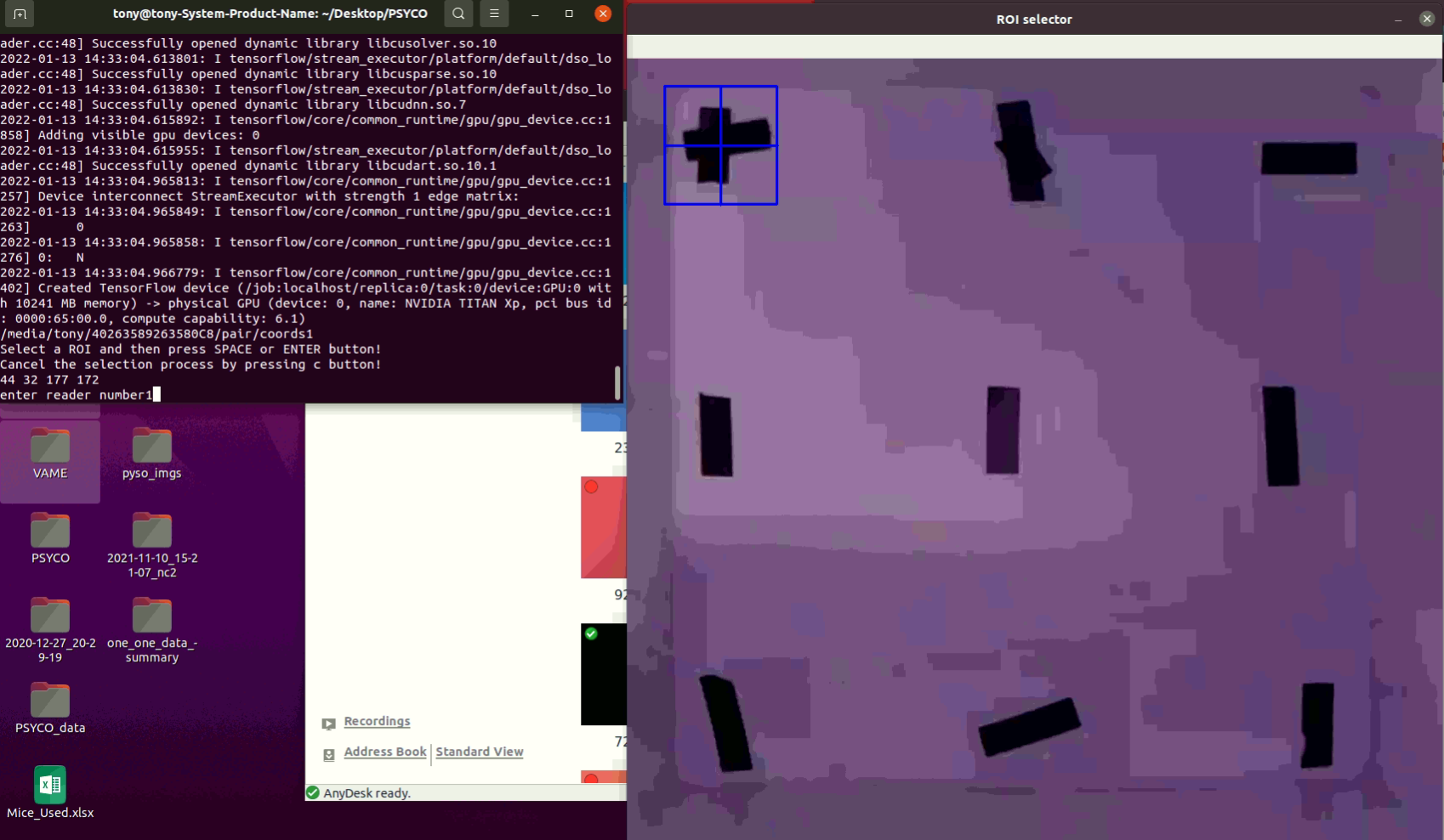


1. After labeling all IDs, a dictionary of reader IDs and their ROIs will be printed in the terminal. Copy and paste the dictionary in the RFID_reader line under the RFID Matching section in the config.ini file. For full details of each parameter, please refer to our Github.


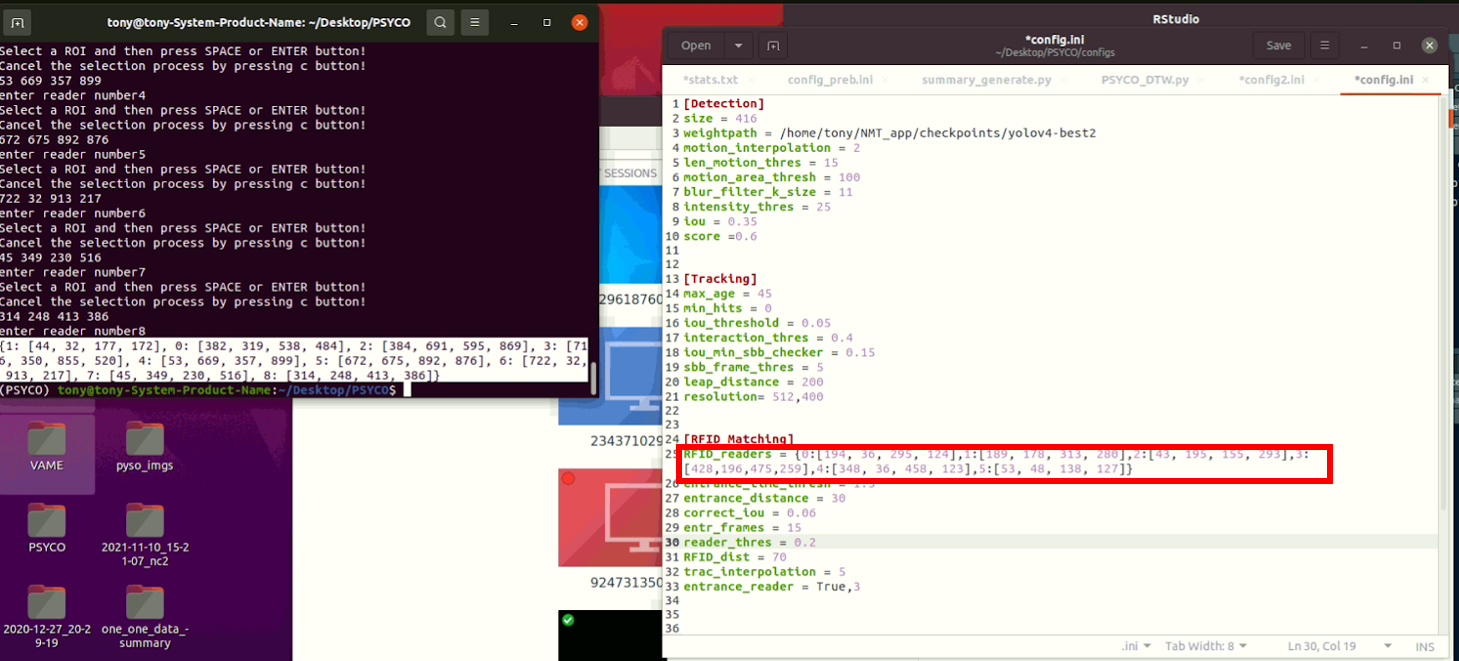


1. Edit the logs.txt file with a text editor. Input the maximum number of mice in the video and the RFID tag ID of each mouse separated by a comma.
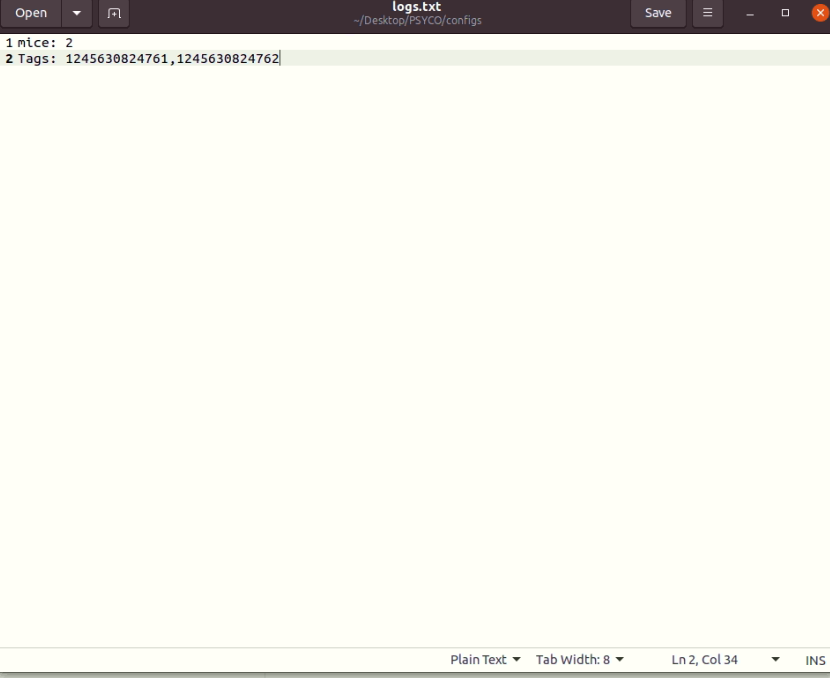

2. To simply analyze one video please run the following command:

python run_analysis.py --folders <path to folder containing data> --config_path <path to config file for running the analysis> –<path to log.txt file>

For further use of the PRT module please refer to the jupyter notebook posted on our Github. We demonstrate sample use of related functions.

**3. Offline Analysis Configurations**

All configurations are in the config.ini file. A template copy is located in the config colder in the PRT directory.

1. **PRT Detection**
   1. **size:** resolution of trained weights, determined at training
   2. **classes:** objects of interest to track. Currently, only supporting one
   3. **Weightpath:** path to trained weights to use for detection
   4. **motion_interpolation:** minimum number of frames where motion did not occur to consider animal as resting
   5. **Len_motion_thres:** minimum number of frames where motion occurred to consider animal as moving
   6. **motion_area_thresh:** minimal area of change in pixels to determine potential animal motion. For finer movements, a lower value would be more sensitive
   7. **blur_filter_k_size: size of Gaussian filter to remove background noise for motion detection**
   8. **intensity_thres:** minimal difference in pixel value between the current frame and moving average to be classified as a potential movement.
   9. **iou: non-max-suppression intersection over union (iou) value. Detections with iou values above this would be counted as one detection**
   10. **score:** minimal confidence of detection (in p-value) to be detected.
2. **Tracking**
   1. **Max_age**: number of frames to keep SORT ID after id disappeared
   2. **min_hits:** minimum number of frames of detection to start SORT tracking
   3. **iou_threshold:** minimum iou value of SORT prediction and current frame detection to be matched with SORT tracking
   4. **resolution:** the resolution of the video input
3. **PRT_RFID_Matching**
   1. **RFID_readers:** a dictionary of RFID reader ID and their respective bounding box on the image
   2. **Entrance_time_thresh:** the duration after video start to start looking at entrance RFID readings
   3. **entrance_distance:** distance to entrance RFID reader to not conduct RFID to SORT ID matching
   4. **correct_iou:** the point in which RFID mismatch correct until,i.e., when the SORT ID had an iou value with another SORT ID
   5. **entr_frames:** previous and future frames to look for to conduct potential RFID to SORT ID matching
   6. **reader_thres:** minimum iou of SORT ID to RFID reader at the time of RFID reading for match to occur
   7. **RFID_dist:** minimum distance of SORT ID center to RFID reader center at the time of RFID reading for match to occur
   8. **trac_interpolation:** interpolate missing tracks when they disappeared under n frames
   9. **entrance_reader:** list value [x,y]. x: True/False, whether an entrance reader is used. y: the entrance RFID reader ID
   10. **itc_slack**: fraction of bounding box enlargement to detect interaction between animals

Note: After running the analysis once, the user can run the following command:

python run_analysis.py --vid_path <path to video folder>

This will provide a folder in the video folder on RFID-SORT ID matching done in the analysis. Users can then adjust for the config file for optimal matches


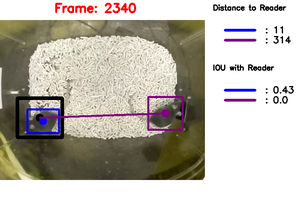


**4. When and How To Train Your Own Weights**

Weights were trained in the original darknet implementation. We understand that installing darknet is not an easy task even for some experienced users. Therefore, we provide a guideline on when to retrain your network and the process involved. In addition, we also provide a linux docker image and an online colab notebook for training. The PRT module is responsible for verifying the accuracy of a given weight, generating the training data, and corresponding configuration files for darknet.

**4.1 Label images for verification and Training**

Before starting, collect sample images of mice using the tracker_rpi as shown in section

1. Activate the conda environment in terminal/prompt:

Conda activate PyMouse(Rodent)Tracks

1. Navigate to the PRT folder and run python:

python

1. Import the following packages in python:

import PyRodentTracks as prt

import os

1. Run the following commands to generate a config folder:

temp = prt.PRT_train(<where you want to generate the folder>,creat_new=True)

1. Edit the config.ini [PRT_Train] in the folder as necessary:
   1. **frames2pic**: number of frames to pick from each video
   2. **size**: resolution of network to train for. Higher values tend to be more accurate at the cost of speed. Must be a multiple of 32.
   3. **classes**: objects to recognize separated by a comma. Currently, only works with one class.
   4. **trainfraction**: the proportion of data to be used as training.
   5. **batch**: batch size for training. Dependent on GPU memory to use.
   6. **random**: 0/1. Data augmentation for training. Dependent on GPU memory to use.
   7. **max_batches**: Maximum number of steps to train. Do not change if possible, the algorithm will automatically calculate the number required.
2. Load the config and add videos to extract for training:

temp.load_config()

temp.add_vids(<list of video paths>)

1. Enter the following command to extract frames to label:

temp.extractframes()

1. Enter the following command to start labeling;

temp.label_imgs()

1. Label the images in labelImg tool :
   1. Save the formate in yolo
   2. Change save dir to image location
   3. Turn on autosave in the view tab
   4. For further information on the gui, please refer to the creator’s Github: <https://github.com/tzutalin/labelImg>


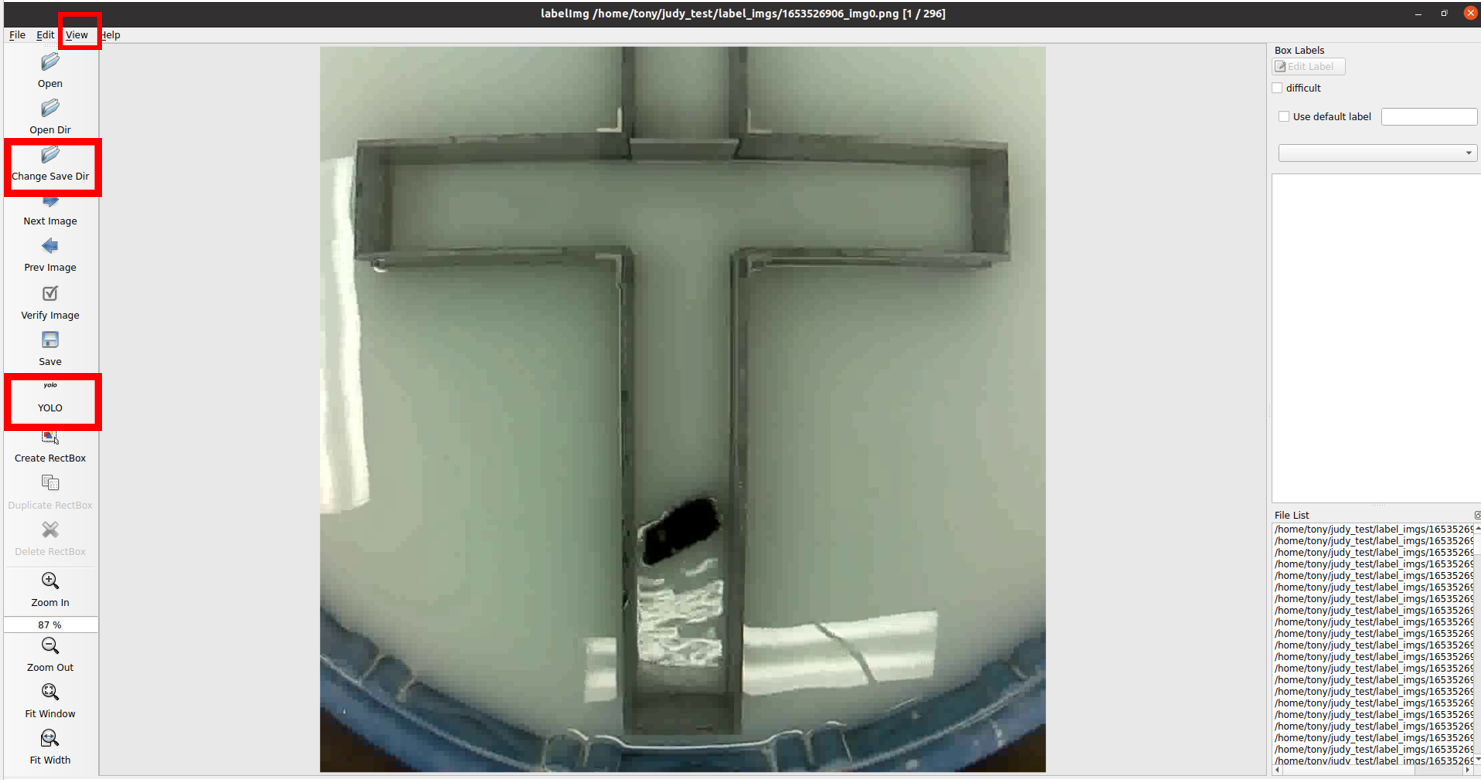


1. Check mAP, false negative, and false positives of a given weight:
   1. Change the weight path in config.ini file in the folder to desired weights
   2. Run the following command for evaluation:

temp.evaluate_weights()

This process might take a while as it will be running detections on all images labelled


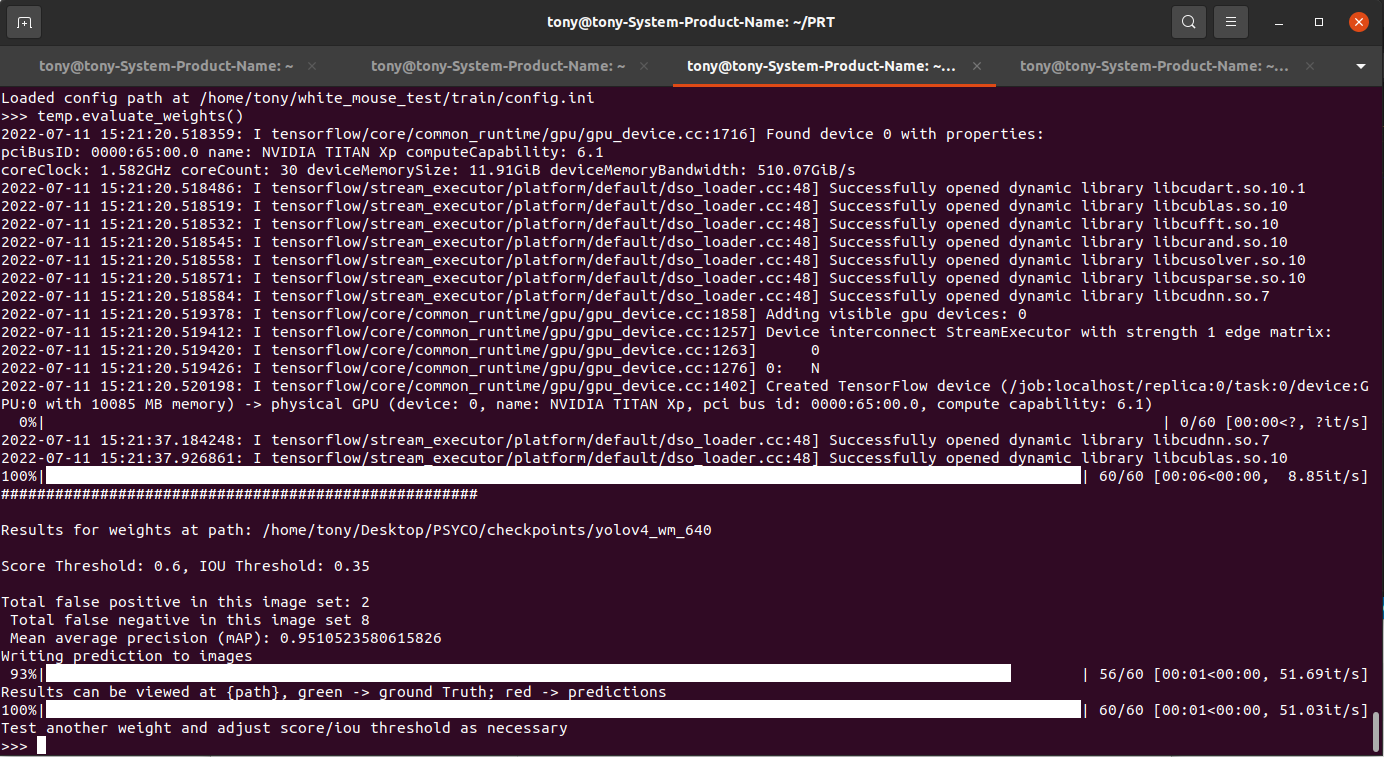


**If an mAP> 98% and has an acceptable incidence of False-negative and positives, you might not have to train new ones at all. Results will also be saved in the training folder**

1. If necessary, users can download the docker or provided notebook on our GitHub for retraining. Instructions are provided in the notebook for training in the cloud.

**4.2 Retraining in Docker (Linux)**

1. For information on docker installation, please refer to the official documentation.

<https://docs.docker.com/get-docker/>

<https://docs.docker.com/engine/install/ubuntu/>

<https://nvidia.github.io/nvidia-docker/>

Please confirm that nvidia-gpu drivers are working before proceeding.

1. Pull the docker image:

docker pull tf4ong/darknet:latest

1. Activate docker with the path of the training folder:

sudo docker run -it -v <path to your training folder>:/root/darknet/trainning/ --gpus all --rm darknet:latest

1. Navigate to darknet folder:

cd darknet

1. Run the following command for training:
2. Results of the training will be saved in a png file in the same folder, cp it to the training folder:

cp chart.png ./trainning/chart.png


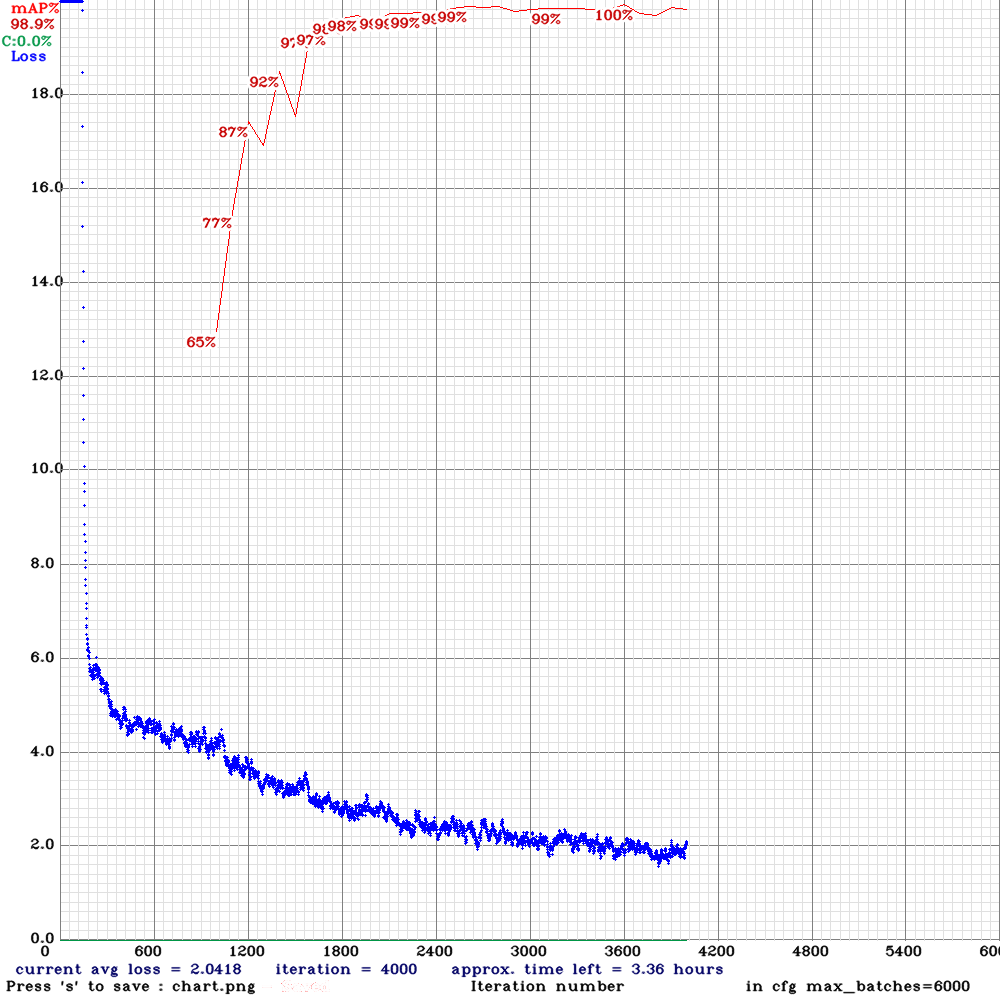


Demonstration Videos: <https://www.youtube.com/playlist?list=PLmcjDqLt_Xk6AAlll3ztvgNI9P3yQxPc2>

**References**

[1. Raspberry Pi Documentation - Camera. *Raspberry Pi* https://www.raspberrypi.com/documentation/accessories/camera.html.](https://www.zotero.org/google-docs/?Y52I1N)

[2. Raspberry Pi Documentation - Getting Started. *Raspberry Pi* https://www.raspberrypi.com/documentation/computers/getting-started.html.](https://www.zotero.org/google-docs/?Y52I1N)

[3. Camera Hardware — Picamera 1.13 Documentation. *Raspberry PI* https://picamera.readthedocs.io/en/release-1.13/fov.html.](https://www.zotero.org/google-docs/?Y52I1N)

[4. Bochkovskiy, A. *Yolo v4, v3 and v2 for Windows and Linux*. (2022).](https://www.zotero.org/google-docs/?Y52I1N)Github repository. https://github.com/AlexeyAB/darknet
